# Structure-guided development of selective M3 muscarinic acetylcholine receptor antagonists

**DOI:** 10.1073/pnas.1813988115

**Published:** 2018-11-07

**Authors:** Hongtao Liu, Josefa Hofmann, Inbar Fish, Benjamin Schaake, Katrin Eitel, Amelie Bartuschat, Jonas Kaindl, Hannelore Rampp, Ashutosh Banerjee, Harald Hübner, Mary J. Clark, Sandra G. Vincent, John T. Fisher, Markus R. Heinrich, Kunio Hirata, Xiangyu Liu, Roger K. Sunahara, Brian K. Shoichet, Brian K. Kobilka, Peter Gmeiner

**Affiliations:** ^a^Beijing Advanced Innovation Center for Structural Biology, School of Medicine, Tsinghua University, 100084 Beijing, China;; ^b^Department of Chemistry and Pharmacy, Medicinal Chemistry, Friedrich-Alexander-Universität Erlangen-Nürnberg, 91058 Erlangen, Germany;; ^c^Department of Pharmaceutical Chemistry, University of California, San Francisco, CA 94158;; ^d^Department of Biochemistry and Molecular Biology, George S. Wise Faculty of Life Sciences, Tel-Aviv University, 6997801 Ramat Aviv, Israel;; ^e^Department of Pharmacology, University of California San Diego School of Medicine, La Jolla, CA 92093;; ^f^Department of Biomedical & Molecular Sciences, Queen’s University, Kingston, ON, Canada K7L 3N6;; ^g^Division of Respirology, Department of Medicine, Queen’s University, Kingston, ON, Canada K7L 3N6;; ^h^Advanced Photon Technology Division, Research Infrastructure Group, SR Life Science Instrumentation Unit, RIKEN/SPring-8 Center, 1-1-1 Kouto, Sayo-cho, Sayo-gun, Hyogo 679-5148, Japan;; ^i^Department of Molecular and Cellular Physiology, Stanford University School of Medicine, Stanford, CA 94305

**Keywords:** muscarinic receptor, G protein-coupled receptor, drug design, subtype selectivity, crystal structure

## Abstract

Drugs that treat chronic obstructive pulmonary disease by antagonizing the M3 muscarinic acetylcholine receptor (M3R) have had a significant effect on health, but can suffer from their lack of selectivity against the M2R subtype, which modulates heart rate. Beginning with the crystal structures of M2R and M3R, we exploited a single amino acid difference in their orthosteric binding pockets using molecular docking and structure-based design. The resulting M3R antagonists had up to 100-fold selectivity over M2R in affinity and over 1,000-fold selectivity in vivo. The crystal structure of the M3R-selective antagonist in complex with M3R corresponded closely to the docking-predicted geometry, providing a template for further optimization.

## Significance

The development of selective antagonists for muscarinic acetylcholine receptors is challenging due to high homology in orthosteric binding sites among subtypes. Starting from a single amino acid difference in the orthosteric pockets in M2 muscarinic acetylcholine receptor (M2R) and M3R, we developed an M3R-selective antagonist using molecular docking and structure-based design. The resulting M3R antagonist showed up to 100-fold selectivity over the M2R in affinity and 1,000-fold selectivity in vivo. The docking-predicted geometry was further confirmed by a 3.1 Å crystal structure of M3R in complex with the selective antagonist. The potential of structure-based design to develop selective drugs with reduced off-target effects is supported by this study.

**Fig. 1.**
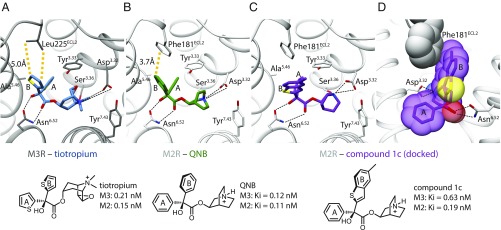
Comparison of the orthosteric binding sites of M2R and M3R. (*A* and *B*) Orthosteric binding pocket of M2R and M3R with conserved features of ligand recognition and binding affinities. The only nonconserved residue in the two binding pockets is located in the second extracellular loop (ECL2) (M2R: Phe181, M3R: Leu225). (*C* and *D*) Docking pose of compound 1c indicating that an enlarged upward-directed ring system can pass the nonconserved Phe181 of the M2 receptor.

While G protein-coupled receptors (GPCRs) are excellent therapeutic targets (1), many GPCR drugs lack selectivity (2). An example are drugs targeting the muscarinic acetylcholine receptor (MR) family, which comprises five subtypes (3–5). Although potent anticholinergics have been developed, most have little subtype selectivity (6) and only avoid debilitating side effects because they can be delivered locally to specific organs. Thus, the antagonist atropine is delivered directly to the eye, while the chronic obstructive pulmonary disease (COPD) drug tiotropium, which reduces bronchoconstriction via the M3 muscarinic acetylcholine receptor (M3R), is delivered by inhalation. This reduces exposure to M2Rs in the heart, averting tachycardia. Even so, antagonism of presynaptic M2Rs in the lung can have unwanted feedback effects on postsynaptic M3Rs by disinhibition of acetylcholine release (7). The recent determination of the crystal structures of four muscarinic receptor subtypes (8–11) allows one to consider a structure-guided approach to subtype-selective ligand design (12, 13). We explored the development of antagonists selective for M3R over M2R, exploiting the single Leu→Phe difference in their orthosteric pockets (Fig. 1 *A* and *B*).

## Results and Discussion

We began with the nonselective antagonists 3-quinuclidinyl-benzilate (QNB) (14) and tiotropium (15), related molecules that adopt similar binding modes in the X-ray structures of M2R and M3R, respectively (8, 9) (Fig. 1 *A* and *B*). Key interactions include a hydrogen bond between Asn^6.52^ and the hydroxyl and ester moieties. The cationic amines ion-pair with Asp^3.32^ in both receptors, and are both enclosed by an aromatic cage composed of Tyr^3.33^, Tyr^6.51^, Tyr^7.39^, and Tyr^7.43^. One aromatic ring (the A ring) stacks with Tyr^3.33^, Trp^4.57^, and Val^3.40^ (8), while the other (the B ring) points toward the extracellular vestibule and interacts with Thr^5.39^, Tyr^3.33^, and Trp^4.57^. The single differentiating interaction is that in M2R, the B ring interacts with Phe181^ECL2^, while in M3R, the residue at the same position is Leu225^ECL2^. Accordingly, we first investigated derivatives with an enlarged “upward”-directed B aryl moiety, synthesizing methylthienyl, benzothienyl, and methylbenzothienyl analogs (*SI Appendix*). Disappointingly, these compounds (Fig. 1*C* and *SI Appendix*, Fig. S1) had little preference for M3R over M2R.

**Fig. 2.**
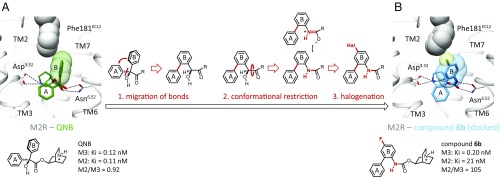
Structure-based ligand design toward a selective M3R antagonist. (*A*) Spatial orientation of QNB ring B and the nonconserved Phe181 in the second extracellular loop (ECL2) of M2R. (*B*) Structural modifications 1, 2, and 3 confer an up-righting and rotation of ring B, as well as steric interactions with Phe181 for compound 6b (105-fold selectivity for M3 over M2).

Docking studies suggested that the tolerance of both muscarinic subtypes toward enlargement of the upward-directed B substituent of QNB or tiotropium may be explained by flexibility of the link between the two arene moieties, allowing the upward-facing ring to avoid a clash with Phe181 (Fig. 1*D* and *SI Appendix*, Figs. S2 and S3). Consequently, a ligand scaffold was sought that enforced a clash with Phe181 of the M2R, and that scaffold was used for a structure-guided optimization of ring B. Three modeled modifications were pursued (Fig. 2). First, we directly linked the benzene ring A to the upward-directed phenyl group B. Because ring A is embedded into a tight hydrophobic pocket (*SI Appendix*, Fig. S4), we expected that directly connecting the rings (16) would enforce a more coplanar orientation and juxtapose the B ring more closely against Phe181^ECL2^ (Fig. 2). Second, we exchanged the CH(OH)C=O unit for a planar NHC=O (17), believing that the more rigid amide would bolster the more “upright” position. Docking supported this, while suggesting that the molecule could retain key hydrogen bonds with Asn^6.52^ via the NHC=O group (*SI Appendix*, Figs. S2 and S3). Third, the docking suggested that a small electronegative substituent would be tolerated at the para position of the B ring, further increasing repulsive interactions with the Phe181^ECL2^ of M2R, while still fitting well with the Leu225^ECL2^ of M3R (Fig. 2*B*). Exploiting our recent synthetic methodology for radical aniline arylation (18), several halogens were installed. Gratifyingly, introduction of a fluorine atom (19, 20) in compound 6b retained strong M3R affinity (*K*_i_ = 0.2 nM), while noticeably reducing M2R binding (*K*_i_ = 21 nM), a 105-fold selectivity for M3R over M2R (Fig. 2*B*). Chloro-, bromo-, and trifluoromethyl analogs also favored M3R over M2R binding, although with lower selectivity (*SI Appendix*, Fig. S5). Chemical modifications, including the synthesis of quinuclidine- and scopine-based quaternary ammonium salts and the bioisosteric exchange of the terminal benzene by a thiophene ring, led ultimately to the antagonist 6o (BS46) (*SI Appendix*, Fig. S5), with M3R *K*_i_ values in the low picomolar range. In functional assays, 6o (BS46) fully inhibits carbachol-induced inositol monophosphate (IP) accumulation as well as β-arrestin recruitment at M3Rs (*SI Appendix*, Fig. S6). Compound 6o (BS46) showed more than 100,000-fold selectivity for muscarinic receptors against 21 aminergic and peptidergic GPCRs (*SI Appendix*, Fig. S7 and Tables S4 and S5).

The accuracy of the 27 pM affinity of 6o (BS46) in equilibrium binding may be affected by ligand depletion. Consistent with this, association and dissociation rates suggested a 12 pM *K*_d_ for 6o (BS46) (*SI Appendix*, Fig. S8 and Table S1), representing a 33-fold preference for M3R over M2R. This selectivity is substantially higher than with the high-affinity antagonist tiotropium (M3/M2 selectivity ∼ 1.5) and similar to the clinically used M3R antagonist darifenacin (M3/M2 selectivity ∼ 25) (*SI Appendix*, Table S1). The dissociation half-life for 6o (BS46) (890 min) is comparable to that of tiotropium (1,300 min) and substantially higher than for darifenacin (140 min). We note that while 6o (BS46) was selective for M3R vs. M2R, the goal of this study, the molecule retained high affinity against the M1R, M4R, and M5R subtypes (*K*_i_ = 0.011 nM, *K*_i_ = 0.009 nM, and *K*_i_ = 0.047 nM, respectively) (*SI Appendix*, Table S2), likely reflecting their conservation of the M3R Leu225^ECL2^ equivalent.

To investigate whether the selectivity of the antagonists reflects the design for preferential binding to Leu225^ECL2^ and against Phe181^ECL2^, we explored the effects of residue substitutions in the M2R and M3R backgrounds (21, 22). In the M2R mutant F181L, the affinity of 6i, 6k, 6l, 6n, and 6o (BS46) improved four- to 29-fold, while their affinity dropped vs. the reciprocal construct M3R L225F (seven- to 48-fold) (*SI Appendix*, Fig. S9). These mutant studies thus support inferences from the modeling and the structure activity relationships, although the F181L mutation in M2R did not increase affinity as much as WT M3R did for all compounds.

**Fig. 3.**
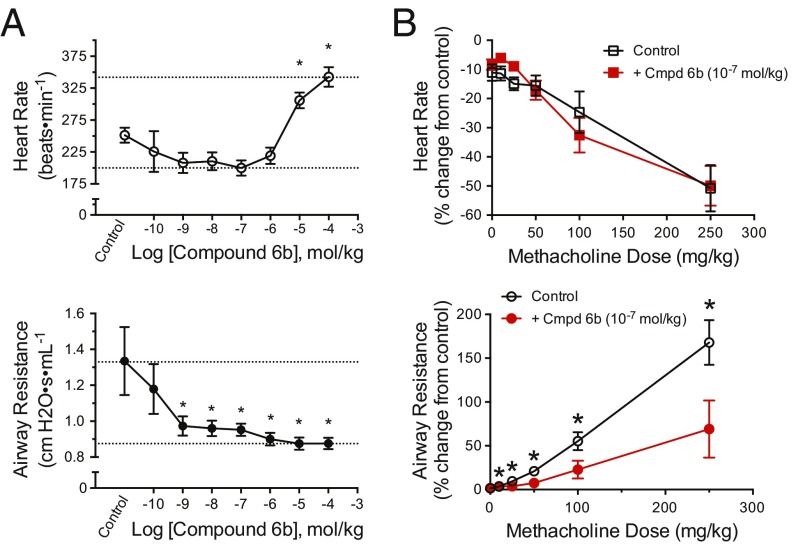
In vivo selectivity of compound 6b. (*A*) Average (±SEM) heart rate (*Top*) and airway resistance (*Bottom*) response to methacholine with a 6b cumulative dose–response curve. Airway resistance was significantly decreased from the control response to methacholine, suggesting inhibition of the M3R at a 6b dose of 10^9^ mol/kg. Heart rate was significantly higher than that of the control, indicating inhibition of the M2R at a 6b dose of 10^5^ mol/kg. The asterisk indicates a significant difference from control: *P < 0.05, one-way repeated-measures ANOVA. (*B*) Average (±SEM) percent change from control heart rate (*Top*) and airway resistance (*Bottom*) response to methacholine dose–response with pretreatment of either saline (black) or 6b 10^7^ mol/kg (red). The airway resistance response to methacholine was significantly different in mice treated with 6b (*P < 0.05) compared with saline. There was no significant difference in the mean heart rate response between treatments. Cmpd, Compound.

We examined the effect of compound 6b on airway resistance, an M3R-mediated response, and heart rate, an M2R-mediated response, following i.v. administration of the nonselective agonist methacholine in C57BL/6 mice (Fig. 3). As expected, methacholine increased airway resistance from 25.3 to 87.6% for individual mice, and reduced heart rate from −13.6 to −22.7% (*P* < 0.05, two-way ANOVA). On i.v. dosing, which ensures systemic exposure, compound 6b reduced the methacholine-induced airway resistance almost fully back to baseline at a dose of 1 * 10^−9^ mol/kg. Conversely, substantial tachycardia was not observed until a dose of 1 * 10^−5^ mol/kg. The order of addition of the agonist or the antagonist did not change the results qualitatively. Thus, pretreatment of the mice with 1 * 10^−7^ mol/kg of compound 6b, followed by injection of methacholine, continued to substantially reduce airway resistance, while no significant difference in mean heart rate was observed at this dose (Fig. 3*B*). Concerning overall in vivo selectivity in the methacholine-provoked effects, the average log IC_50_ values are −8.8 ± 0.6 M (or 1.6 nM) for airway response and −5.2 ± 0.1 M (or 6.3 μM) for heart rate response, suggesting an in vivo M3/M2 selectivity of about 4,000-fold.

**Fig. 4.**
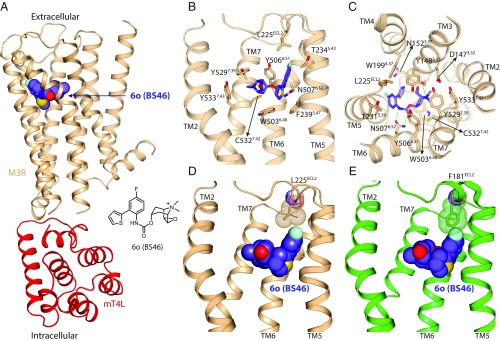
Comparison of the orthosteric binding sites of M2R and M3R. (*A* and *B*) Crystal structure of M3R in complex with the selective antagonist 6o (BS46). (*A*) Overall structure of the M3R/mT4L/6o (BS46) complex. (*B* and *C*) Binding-pocket residues of M3R interacting with 6o (BS46). (*D* and *E*) Interaction of 6o (BS46) with a nonconserved position in the second extracellular loop (ECL2) of M2R and M3R. (*D*) Crystal structure shows an interaction of the fluorine group of 6o (BS46) with Leu225 in the ECL2 of M3R. (*E*) Superimposed structure of M2R on the M3R/6o (BS46) structure indicates a steric clash between Phe181 of M2R and the fluorine of 6o (BS46).

To test the model upon which these molecules were based and to provide a template for future design, the structure of the M3R/6o (BS46) complex was determined by X-ray crystallography. An M3R-mT4L fusion protein (23) was expressed and purified in the presence of 6o (BS46), and crystals were grown in lipid cubic phase. We obtained a 3.1 Å dataset from 93 crystals and solved the structure by molecular replacement (Fig. 4*A* and *SI Appendix*, Table S3). The electron density for 6o (BS46) was unambiguous in a Fo-Fc–simulated annealing omit map (*SI Appendix*, Fig. S10). Compound 6o (BS46) binds to M3R in the predicted orientation, making all interactions predicted in the model, with the fluorine oriented toward Leu225^ECL^ (Fig. 4 *B* and *C*); overall, the crystallographic result superimposes on the docking prediction with an rmsd of 0.377 Å (*SI Appendix*, Fig. S11). When superimposed on the M3R/6o (BS46) complex, the fluorine would sterically clash with Phe181^ECL2^ of M2R (Fig. 4 *D* and *E*).

Several caveats bear mentioning. After this work was completed, we discovered that intermediate compounds 3 and 4, two weakly selective antagonists that we studied to validate our model, were part of a series of M3 antagonists developed by Yamanouchi pharmaceuticals and others (24, 25). The synthesis of compounds 6a and 6b had been described in a patent from Astellas (26); however, the activity of these two molecules was not defined. Along these lines, our compounds of type 6 are not the very first antagonists selective for the M3R vs. the M2R (although such molecules remain very rare). Indeed, the drug darifenacin, a scaffold unrelated to that explored here, shows remarkable selectivity (*K*_i_ = 0.25 and *K*_i_ = 19 nM for M3R and M2R, respectively). Our selectivity goal was narrow, improving activity for M3R at the expense of M2R, which is the most relevant antitarget in the periphery, as quaternary amines will not cross the blood–brain barrier. The selectivity over M2R is enabled by an L225→Phe substitution in the orthosteric site, while in the other three muscarinic subtypes, M1R, M4R, and M5R, Leu225 is conserved, and so the compounds show no selectivity against these receptors. Finally, 6o (BS46) is a lead and not a drug candidate; further structure-activity relationship and pharmacokinetics studies would be necessary to develop this compound family.

These caveats should not obscure the main conclusions from this work. Whereas some of the molecules described have been previously investigated, little is known about their activity, their structural recognition, or their pharmacology. While darifenacin is selective in vitro, it is a bladder-directed drug that is much less potent in respiratory disease (27), where it activity is complicated by its problematic metabolism and short half-life (28). Two observations from this study merit particular emphasis. First, compounds like 6o (BS46) can have important clinical applications for the treatment of COPD and asthma. The long-acting muscarinic antagonists currently approved for treatment of COPD are all nonselective for the M3R vs. M2R in binding assays. They achieve some in vivo selectivity by inhalation and by their slower dissociation from the M3R relative to the M2R (29). However, 6o (BS46) has a dissociation rate from the M3R that is comparable to that of tiotropium, but is also selective over the M2R. Thus, compounds like 6o (BS46) may have efficacy comparable to these approved M3R antagonists for asthma and COPD, without the off-target effects on the M2R in the heart or in parasympathetic neurons in the lung. Second, and more generally, the structure-based strategy used here may prove useful for other GPCR families that are highly related by subtype, such as the nine adrenaline, 13 serotonin, and five dopamine receptors, among others.

## Materials and Methods

### Ligand Design.

MR ligand design was guided by structures of the M2R inactive-state crystal structure [Protein Data Bank (PDB) ID code 3UON] bound to the antagonist QNB and the M3R inactive structure (PDB ID code 4DAJ) bound to the inverse agonist tiotropium. We used Dock 3.6 (30) to perform virtual docking against these structures. Further details of the ligand synthesis and docking are provided in *SI Appendix*.

### Characterization of Ligands.

Synthesized ligands were characterized by ligand binding, arrestin recruitment, and inositol monophosphate accumulation assays as described in *SI Appendix*. The effect of compound 6b on heart rate and airway resistance was determined in mice as described in *SI Appendix*.

### Structure Determination.

The structure of M3R bound to compound 6o was determined by crystallography in the lepidic cubic phase. Data collection was performed at beamline BL32XU at SPring-8. Diffraction data were processed from 93 crystals by XDS (31). The structure was solved by molecular replacement using the previously reported M3-mT4L (4U15) structure as the searching model. Structure refinement was performed with phenix.refine. The final model was validated with MolProbity. All structure figures were prepared with PyMOL. Further details are provided in *SI Appendix*.

## Supplementary Material

Supplementary File
