# Supplementary material for: Structure-guided development of selective M3 muscarinic acetylcholine receptor antagonists
Source: Proc Natl Acad Sci U S A. 2018 Nov 7;115(47):12046–50. doi: 10.1073/pnas.1813988115 (PMC6255194; doi:10.1073/pnas.1813988115)
Supplement: Supplementary File [file pnas.1813988115.sapp.pdf]

PNAS
www.pnas.org

Supplementary Information for

Structure-guided development of selective M3 muscarinic acetylcholine receptor antagonists

Hongtao Liu[a, 1], Josefa Hofmann[b, 1], Inbar Fish[c,d, 1], Benjamin Schaake[b], Katrin Eitel[b], Amelie Bartuschat[b], Jonas Kaindl[b], Hannelore Rampp[b], Ashutosh Banerjee[b], Harald Hübner[b], Mary J. Clark[e], Sandra G. Vincent [f,g], John T. Fisher[f,g], Markus R. Heinrich[b], Kunio Hirata[g], Xiangyu Liu[a, 2], Roger K. Sunahara[e], Brian K. Shoichet[c, 2], Brian K. Kobilka[a,h, 2], Peter Gmeiner[b, 2].

[a]Beijing Advanced Innovation Center for Structural Biology, School of Medicine, Tsinghua University, Beijing 100084, China.

[b]Department of Chemistry and Pharmacy, Medicinal Chemistry, Friedrich Alexander University, Nikolaus-Fiebiger-Straße 10, 91058 Erlangen, Germany.

[c]Department of Pharmaceutical Chemistry, University of California, San Francisco, California 94158, USA.

[d]Department of Biochemistry and Molecular Biology, George S. Wise Faculty of Life Sciences, Tel-Aviv University, Ramat Aviv, Israel.

[e]Department of Pharmacology, University of California San Diego School of Medicine, 9500 Gilman Drive, La Jolla, California 92093, USA.

[f]Department of Biomedical & Molecular Sciences and Division of Respirology, Department of Medicine, Queen's University, Kingston, Ontario K7L 3N6, Canada.

[g]Advanced Photon Technology Division, Research Infrastructure Group, SR Life Science Instrumentation Unit, RIKEN/SPring-8 Center, 1-1-1 Kouto Sayo-cho Sayo-gun, Hyogo 679-5148 Japan.

[h]Department of Molecular and Cellular Physiology, Stanford University School of Medicine, Stanford, California 94305, USA.

[1]H. L., J. H., and I. F. contributed equally to this work.

www.pnas.org/cgi/doi/10.1073/pnas.1813988115

[2]To whom correspondence may be addressed. Email: liu_xy@mail.tsinghua.edu.cn; bshoichet@gmail.com; kobilka@stanford.edu; or peter.gmeiner@fau.de

This PDF file includes:

Supporting Materials and Methods
Figs. S1 to S11
Tables S1 to S5

Supporting Materials and Methods

A structural model for binding & specificity

We docked the most specific antagonists into the M2R and M3R crystal structures (using DOCK3.6 (1)), relaxing the resulting complexes by molecular mechanics minimization (using AMBER (2, 3)). As expected, the docking scores, even against the relaxed structures, were unable to track the changes in affinity and specificity observed over this series, which after all amounted to changes of no more than four kcal/mol, substantially less than expected for docking accuracy (4). Nevertheless, the geometries that emerged illuminate the recognition of the more potent and specific antagonists. In the calculated complexes of **6a-6f,** the cationic nitrogen hydrogen-bonds with the key recognition Asp$^{3.32}$ and forms cation-quadrupolar interactions with tyrosines 148$^{3.33}$, 529$^{7.39}$ and 533$^{7.43}$. These resemble the ionic interactions made with tiotropium in its complex with the M3 receptor, except that in the new inhibitors the tertiary cationic nitrogen itself is modelled to hydrogen bond with the aspartate, while in the quaternary tiotropium this interaction is mediated with a methyl off the nitrogen. Meanwhile, the carbamate moiety, first introduced in compound **4** and prominent in the more specific **6** series of compounds, hydrogen bonds with the conserved Asn$^{6.52}$. In the modelled complexes of **6a-g**, the carbonyl oxygen of the carbamate interacts with the Nδ of the asparagine, typically with a distance of 2.8 Å, while the carbamate nitrogen does the same with the asparagine Oδ at the same distance. A related interaction is observed in tiotropium, but instead of an amide—amide pairing, the tiotropium engages its ester carbonyl and its

hydroxyl, and at distances that fall outside classical limits for hydrogen bonds (3.4 Å to each).

In both the M2R and the M3R modeled complexes, it is the combination of the ionic interactions with the tertiary amine, and the apparently favorable hydrogen-bonding with the carbamate, that essentially fix the position of specificity conferring halo-diaryl system. In the highest specificity compound **6b**, for instance, the compound's fluorine is juxtaposed with the one point of difference between the M3R and M2R orthosteric sites, the Phe181/Leu225, with which it forms a van der Waals interaction (distance 3.2-3.3 Å) (**Fig. S3**). The same may be said for the other **6** series compounds, which also make this interaction with similar groups, or in the case of **6g**, with a trifluoro-methyl group. It may be the combination of multiple polar interactions, engaging multiple receptor residues and occur at three different points, and the insertion of small steric perturbations right at the single point of difference between the two receptors, that has led to the unprecedented ability of compounds like **6b** to distinguish between the almost identical orthosteric sites of M2R and M3R.

Docking

We used Dock 3.6 (1) to perform virtual docking against the M2R inactive state crystal structure (PDB ID 3UON), bound to the antagonist QNB and the M3R inactive structure (PDB ID 4DAJ), bound to the inverse agonist tiotropium. Complementarity of each ligand pose is scored as the sum of the receptor-ligand electrostatic and van der Waals interaction energy and corrected for ligand desolvation. Partial charges from the united-atom AMBER force field were used for all receptor atoms except for $Asn^{6.52}$ and $Asp^{3.32}$ for which the dipole moment was increased as previously described (5) to boost electrostatic scores for poses in polar contact with these important residues.

Pose optimization

Using Amber software (2, 3) we performed a minimization of the docked complexes. The starting structures were taken from the docked pose. The structures were subjected to 10000 steps of conjugate gradient minimization. Following that we rescored the poses using DOCK.

Protein expression and purification

A M3-mT4L construct *(23)* was used in this study. Protein expression and purification was performed as previously described *(9, 11)*. The rat M3 receptor was expressed Sf9 cells (obtained from Expressions Systems, LLC) using the baculovirus system. Infection was performed at 4X 10^6 cells per ml and flasks were shaken at 27℃ for 60h following infection in the presence of 100 nM selective antagonist **6o (BS46)**. Cells were harvested by centrifugation, then lysed by osmotic shock in the presence of 100 nM **6o (BS46)**, which was present in all subsequent buffers. Receptor was extracted from cell membranes with solubilization buffer of 20 mM HEPES pH 7.5, 1% dodecyl maltoside (DDM), 0.03% cholesterol hemisuccinate (CHS), 750 mM NaCI, and 30% glycerol. Iodoacetamide (2 mg ml^{-1}) was added to block reactive cysteines at this stage. Nickel-NTA agarose was added to the solubilizaed receptor without prior centrifugation. After stirring for 2 h at 4℃, receptor bound nickel resin was washed in batch with 3000 rpm spins for 1

min each. Washed resin was poured into a glass column, and receptor was eluted in 20 mM HEPES pH 7.5, 0.1% DDM, 0.03% CHS, 750 mM NaCI and 250 mM imidazole. Nickel-NTA agarose resin-purified receptor was then loaded by gravity flow over M1-Flag affinity resin (Sigma). Following extensive washing, detergent was gradually exchanged over 1 h into a buffer in which DDM was replaced with 0.01% lauryl maltose neopentyl glycol (MNG). Receptor was eluted with 0.2 mg ml^{-1} Flag peptide and 5 mM EDTA. TEV protease (1:10 w/w) was added and incubated with receptor for 1.5 h at room temperature to remove the flexible N-terminal tail. Receptor was then separated from TEV by size exclusion chromatography (SEC) on a Sephadex S200 column (GE Healthcare) in a buffer of 20 mM HEPES pH 7.5, 0.01% MNG, 0.001% CHS and 100 mM NaCI. **6o (BS46)** was added to a final concentration of 10 μM following SEC. The purified M3 receptor was concentrated to ~40 mg ml^{-1} with a 50 kDa cutoff Amicon centrifugal filters (Millipore).

Crystallization

Concentrated M3 receptor was mixed with monoolein (Sigma) containing 10% (w/w) cholesterol (Sigma) to form lipidic cubic phase (LCP) using the two-syringe reconstitution method (6). The resulting lipidic cubic phase mix was dispensed in 15 nl drops onto 96-well glass sandwich plates and overlaid with 600 nl precipitant solution using a Gryphon LCP crystallization robot (Art Robbins Instruments). Crystals with best diffraction grew after 2-3 days in precipitant solution consisting of 40-45% PEG 300, 100 mM Tris-HCI pH 7.5, 300 mM Ammonium citrate, 0.01M Sodium Fluoride, 200 μM **6o (BS46)**.

Data collection and structure determination

Crystals were harvested in 50 um MicroMeso loops (MiTeGen) and flash-frozen in liquid nitrogen. Data collection was performed at beamline BL32XU at Spring-8, Japan. Typically, 6 to 18 degrees of data were collected for each crystal. Diffraction data were processed from 93 crystals by XDS (7). The structure was solved by molecular replacement using previous reported M3-mT4L (4U15) structure as searching model. Structure refinement was performed with phenix.refine. Final model was validated with Molprobity. All structure figures were prepared with PyMol.

Site-directed mutagenesis

Two mammalian expression plasmids coding for the human M2 muscarinic receptor (M2R-pcDNA3.1+) and human M3 muscarinic receptor (M3R-pcDNA3.1+) were obtained from the Missouri S&T cDNA Resource Center. Mutant M2 receptors (M2R_F181L) and M3 receptors (M3R_L225F) were generated by using the one-step site-directed mutagenesis according to instructions (8) and confirmed by DNA sequencing.

Radioligand binding analysis - Receptor binding

CHO cells stably transfected with hM1R, hM2R, hM3R-Δi3, hM4R, or hM5R were harvested for membrane preparation by rinsing the cells with PBS and lifting with harvesting buffer (0.68 mM EDTA, 150 mM NaCl, 20 mM HEPES, pH 7.4), and centrifuged at 200xg for 3 min. The cells were resuspended in ice cold homogenizing buffer (10 mM HEPES, pH 7.4, 10 mM NaCl, 0.5 mM MgCl2, 0.5 mM EGTA), homogenized using a Tissue Tearer (BioSpec) for 30 sec, and centrifuged at 20,000xg for 20 min. The

pellet was resuspended and homogenized for 10 s and centrifuged again before final resuspension in binding assay buffer (10 mM HEPES, pH 7.4, 10 mM NaCl, 0.5 mM $MgCl_2$), using a Dounce glass homogenizer and stored at -80°C until use.

For equilibrium binding assays, cell membranes (5-30 µg) were incubated for 3 h at room temperature with [^{3}H]-N-methyl-scopolamine, [^{3}H]-NMS (70 Ci/mmol from Perkin Elmer, Boston, MA). For saturation isotherms 0.02-2 nM [^{3}H]-NMS was used, whereas for competition binding, a concentration between 0.05-0.2 nM, depending on the mAChR subtype. Assays were performed in binding assay buffer (above) with 0-1 µM test compound (for competition) or 20 µM atropine (to determine non-specific binding). Sample membranes were harvested by vacuum filtration on GF/C filter plates, washed with ice cold binding buffer to remove unbound radioligand, and allowed to dry before adding Microscint (Perkin Elmer) for counting in a Top Count Scintillation Counter (Packard). Data were analyzed using GraphPad Prism to determine equilibrium binding constants. All data were fit using a least squares analysis to a single site to determine the Kd, or calculate IC_{50}. Ki's were determined from the IC_{50} values according to Cheng and Prusoff (9).

Radioligand binding to the mutants M2R_F181L and M3R_L225F were performed using membrane preparations from HEK293T cells which were transiently transfecting with cDNA of the appropriate mutant receptor using a solution of linear polyethyleneimine in PBS (10) at a final concentration of 4-8 µg/test tube, receptor densities of 1100 fmol/mg, and 1500 fmol/mg, Kd values of 0.25 nM, and 0.18 nM and [^{3}H]-NMS at 0.30 nM and 0.20 nM for M2R_F181L and M3R_L225F, respectively.

Association and dissociation rate constants for unlabeled ligands were measured using the competition association assay described in Guo *et al.(11)* with [^{3}H]-NMS and the unlabeled ligand. The dissociation rate constant for [^{3}H]-NMS, which was used for the analysis of the competition association assay, was determined by incubating cell membranes (10-30 µg) with 0.3 nM of [^{3}H]-NMS (with or without 20 µM atropine to determine non-specific binding) in binding assay buffer (M2R) or 100 mM NaCl and 20 mM HEPES, pH 7.4 (M3R) for 1 h at room temperature, followed by addition of an equal volume of atropine was (for a final concentration of 50 µM to prevent re-association of [^{3}H]-NMS. Membranes were harvested after 2-80 min (M2R) or 5-180 min (M3R) of dissociation and specific binding at the different time points was fit to a one phase exponential decay curve with GraphPad Prism to determine the dissociation rate constant. The association binding rate of [^{3}H]-NMS was determined by incubating cell membranes (10-30 µg) with 2 concentrations of [^{3}H]-NMS (i.e. 0.1 and 0.2 nM in the same assay) for 5-180 min at room temperature and analysing the specific binding using the association kinetics analysis in GraphPad Prism. The binding kinetics of unlabeled ligands was then determined by incubating cell membranes (10-30 µg) with 2-3 different concentrations of unlabeled ligand and 0.1 or 0.2 nM [^{3}H]-NMS (for M3R or M2R, respectively) for 5-180 min at room temperature and analysing the specific binding using the kinetics of competitive binding analysis in GraphPad Prism.

Determination of receptor specificity

Binding affinities for 21 GPCRs which are different from the muscarinic receptor family M1–M5 have been determined by radioligand binding experiments (18 receptors) or by measuring an inhibitory effect in an IP accumulation assay (three receptors). In radioligand binding experiments the ability of compound **6o (BS46)** to displace a

radioligand at a concentration of 10 µM was tested at membranes of cells transiently or stably expressing the appropriate receptor according to the conditions listed in Table S4 and Table S5 and according to protocols described above. Determination of an inhibitory effect at the PAR-2 receptor and orexin receptor subtypes 1 and 2 was performed applying the IP-One HTRF® assay (Cisbio) according to the manufacturer's protocol and as described below. In brief, HEK-293 cells were transiently transfected with the cDNAs of the human PAR-2 receptor (gift of Prof. Korbmacher, Institute of Cellular and Molecular Physiology, FAU), the orexin receptor OXR1 (cDNA Resource Center) and OXR-2 (Genscript, Piscataway, NJ) applying Mirus TransIT-293 transfection reagent (Peqlab). Inhibitory effects were determined by preincubating compound **6o (BS46)** (10 µM) for 30 min followed by addition of the EC_{80} concentration of the appropriate agonist and subsequently incubation for 90 min. Values representing the displacement of the reference were determined for the data derived from the radioligand binding experiment by normalizing to 0% (specific binding) and 100% (unspecific binding) and for data from the functional test by normalizing to 0% (full agonist effect) and 100 % (basal activity). If an inhibitory effect of **6o (BS46)** was greater than 15% complete dose-response curves were measured to get the corresponding Ki value.

Accumulation of inositol mono phosphate (IP) as functional assay for antagonist properties

Determination of inhibitory effect of **6b**, **6o (BS46)** and tiotripium at the M3 receptor was performed applying the IP-One HTRF® assay (Cisbio, Codolet, France) according to the manufacturer's protocol. In brief, HEK-293 cells were grown to a confluence of approx. 70% and transiently transfected with the cDNA of the human M3R (cDNA Rescourse Center, Bloomsberg, PA) applying Mirus TransIT-293 transfection reagent (Peqlab, Erlangen, Germany). After one day cells were detached from the culture dish with Versene (Life Technologies, Darmstadt, Germany), seeded into black 384-well plates (10000 cells/well) (Greiner Bio-One, Frickenhausen, Germany) and maintained for 24 h at 37 °C. Antagonist properties were determined by preincubating the test compounds (final range of concentration from 0.1 nM up to 10 µM) for 30 min at 37°C followed by addition of 100 nM of carbachol (final concentration) and further incubation for 1 h at 37°C. Incubation was stopped by adding detection reagents (IP1-d2 conjugate and Anti-IP1cryptate TB conjugate each dissolved in lysis buffer) for further 60 min at room temperature. Time resolved fluorescence resonance energy transfer (HTRF) was determined using the Clariostar plate reader (BMG, Ortenberg, Germany). Data analysis was performed by nonlinear regression using the algorithms for log(agonist) vs. response of PRISM 6.0 (GraphPad, San Diego, CA) and normalization of the raw data to basal (0%) and the maximum effect of carbachol (100%).

Recruitment of β-arrestin-2 as functional assay for antagonist properties.

Antagonist properties of **6b**, **6o (BS46)** and tiotripium mediated by the arrestin signaling pathway and the M3 receptor was determined applying the PathHunter® assay (DiscoverX, Birmingham, U.K.) as described in the manufacturer's protocol. In brief, HEK-293 cells stably expressing the enzyme acceptor (EA) tagged β-arrestin-2 fusion protein were transiently transfected with the ProLink tagged M3-PK1 construct employing Mirus TransIT-293 transfection reagent. After 24 h cells were transferred into white clear bottom 384-well plates (5000 cells/well) (Greiner Bio-One) and maintained for further 24

h at 37 °C, 5 % CO2. After preincubation with the test compounds in a range from 0.01 nM to 1 μM dissolved in PBS for 30 min at 37°C, carbachol (50 μM) was added and incubation was continued for 150 h at 37 °C. Stimulation was stopped by addition of detection mix for 60 min at room temperature. Chemiluminescence was determined using a Clariostar plate reader. Data analysis was done by nonlinear regression using the algorithms for log(agonist) vs. response of PRISM 6.0 (GraphPad, San Diego, CA) and normalization of the raw data to basal (0%) and the maximum effect of carbachol (100%).

In Vivo Physiology – airway resistance and heart rate studies in mice

Experimental procedures met the guidelines of the Canadian Council on Animal Care (CCAC) and were approved by the Queen's University Animal Care Committee. Adult, male C57BL/6J mice (The Jackson Laboratory, Bar Harbour, ME) were anaesthetized with an intraperitoneal injection of sodium pentobarbital (60 mg/kg) and subcutaneous needle electrodes were inserted to monitor heart rate. Mice were ventilated using a custom Mod RV small animal ventilator (Voltek Enterprises, Toronto, Canada (12) at a rate of 120 breaths per minute and a tidal volume of 7.5 ml/kg with a 40% oxygen/balance nitrogen gas mixture. The right jugular vein was cannulated for the intravenous administration of supplemental anaesthetic and/or paralyzing agent (Pancuronium Bromide 0.25 mg/kg 0.25 mg/ml). Paralyzing agent was given after depth of anaesthesia was confirmed and prior to experimental procedures. Breath-by-breath measurements were made of tidal volume, airway flow and airway pressure. Maximal airway resistance and compliance values were derived. Individual heart rate and airway resistance responses were plotted and individual half maximal inhibitory concentration (IC50) values for heart rate and airway resistance responses were calculated using statistical software (GraphPad Software Inc, La Jolla, CA, USA). Log IC50 values was calculated for each individual mouse. Average +/- SEM of Log IC50 were calculated, then converted to IC50.

Control response to repeat injections of Methacholine (MCh)

To quantify the consistency of the cardiac and bronchoconstriction response to Methacholine (MCh) a 50 μg/kg dose was injected intravenously every 10 minutes in a group of 6 adult, male, C57BL/6 mice (n=6, 23.4 ± 0.4 gm). Heart rate and airway resistance response were measured and responses between doses compared.

Determining IC50 values for M3R antagonists

To determine the IC50 of each M3R antagonist on MCh responses, two separate groups of mice received intravenous injections of **6b** (n=8, 20.2 ±0.2 gm) in a cumulative dose-response fashion (1 x 10^{-10} to 2 x 10^{-5} Mol/kg;) prior to repeat 50 μg/kg doses of MCh. Heart rate and airway resistance responses were measured and individual IC50 values for each parameter determined.

In vivo selectivity of M3R antagonists for airway resistance vs heart rate

To compare the selectivity for bronchoconstriction (M3) versus bradycardia (M2) in each antagonist (13), three separate groups of mice were pretreated with either saline

(control) or a single dose (1e-07 Mol/kg) of **6b** (n=7) prior to a MCh cumulative dose-response curve (10, 25, 50, 100 and 250 μg/kg). Rrs and HR responses were measured and expressed as a percentage change from control values.

General materials and methods for organic synthesis

Dry solvents and reagents were of commercial quality and were used as purchased. ESI-TOF high mass accuracy and resolution experiments were performed on a AB Sciex Triple TOF660 SCiex or on a Bruker maXis MS. EI high mass accuracy and resolution experiments were performed on a GC mate2 JEOL mass spectrometer. NMR spectra were obtained on a Bruker Avance 360 (^{1}H at 360 MHz, ^{13}C at 90 MHz), a Bruker Avance 400 (^{1}H at 400 MHz, ^{13}C at 100 MHz) or a Bruker Avance 600 (^{1}H at 600 MHz, ^{13}C at 150 MHz) spectrometer at 298 K using the solvents indicated. Chemical shifts are reported relative to TMS, acetone or to the residual solvent peak. IR spectra were performed on a Jasco FT/IR 4100 spectrometer (film on a NaCl crystal). Purification by flash chromatography was performed using Silica Gel 60 (40-63 μm mesh) from Merck as stationary phase; TLC analyses were performed using Merck 60 F254 aluminum sheets and the spots were visualized under UV light (254 nm) and with reagents such as $KMnO_4$ vapor. Purification by preparative RP-HPLC was performed on AGILENT 1100 Preparative Series equipped with a VWD detector (230 nm; 254 nm), column 1: MACHEREY-NAGEL Varioprep VP 250/32 NUCLEODUR C18 HTec 5 μm particles [C18], flow rate: 32 mL/min, employing solvent systems as specified below or column 2: MACHEREY-NAGEL Varioprep VP 250/10 NUCLEODUR C18 HTec (5 μm), flow rate: 4 mL/min, employing solvent systems as specified below or column 3: Zorbax Eclipse XDB-C8 30x150 mm 5 μm particles [C8], connected to a VDSpher PUR C8-E 30x20 mm 10 μm particles [C8], flow rate 30 mL/min, employing solvent systems as specified below. Analytical HPLC/MS was either performed on AGILENT 1100 series HPLC system employing a VWL detector (220 nm or 254 nm) using a ZORBAX ECLIPSE XDB-C8 (4.6 x 150 mm, 5 μm) HPLC column, using mass detection on a Bruker Esquire 2000 ion-trap mass spectrometer with ESI or APCI ionization source; or on a Thermo Scientific Dionex Ultimate 3000 HPLC system using DAD detection (230 nm; 254 nm) equipped with a Kinetex 2.6u mesh C8 100A (2.1 x 75 mm, 2.6 μm) HPLC column. Mass detection was performed with a BRUKER amaZon SL mass spectrometer using ESI or APCI ionization source. The purity of all test compounds and key intermediates was determined by reverse phase HPLC. HPLC analysis was performed on analytical systems (Agilent 1200 analytical series, DAD detector 230 nm, 254 nm or Varian 940-LC, PDA detector 254 nm, 280 nm); System A: Zorbax Eclipse XDB-C8 analytical column, 4.6 mm x 150 mm, 5 μm, flow rate: 0.5 mL/min (Agilent) or XRs C8 column, RP-8, 4.6 x 150 mm, 5 μm, 0.5 – 1.0 mL/min (Varian), Eluent: CH_3OH in H_2O+0.1% HCO_2H (0 - 3 min 10 %, 3 - 18 min 10 – 100%, 18 - 24 min 100%); System B: Zorbax Eclipse XDB-C8 analytical column, 4.6 mm x 150 mm, 5 μm, flow rate: 0.5 mL/min (Agilent) or XRs C8 column, RP-8, 4.6 x 150 mm, 5 μm, 0.5 – 1.0 mL/min (Varian), Eluent: CH_3OH in H_2O+0.1% F_3CCO_2H (0 - 3 min 5 %, 3 - 18 min 5 – 100%, 18 - 24 min 100%); System C: Zorbax Eclipse XDB-C8 analytical column, 4.6 mm x 150 mm, 5 μm, flow rate: 0.5 mL/min (Agilent) or XRs C8 column, RP-8, 4.6 x 150 mm, 5 μm, 0.5 – 1.0 mL/min (Varian), Eluent: CH_3CN in H_2O+0.1% F_3CCO_2H (0 - 3 min 5 %, 3 - 18 min 5 – 95%, 18 - 24 min 95%); System D: Zorbax Eclipse XDB-C8 analytical column, 4.6 mm x 150 mm, 5 μm, flow rate: 0.5 mL/min (Agilent) or XRs

C8 column, RP-8, 4.6 x 150 mm, 5 µm, 0.5 – 1.0 mL/min (Varian), Eluent: CH_3CN in H_2O+0.1% HCO_2H (0 - 3 min 5 %, 3 - 18 min 5 – 95%, 18 - 24 min 95%).

Synthetic procedures for the target compounds

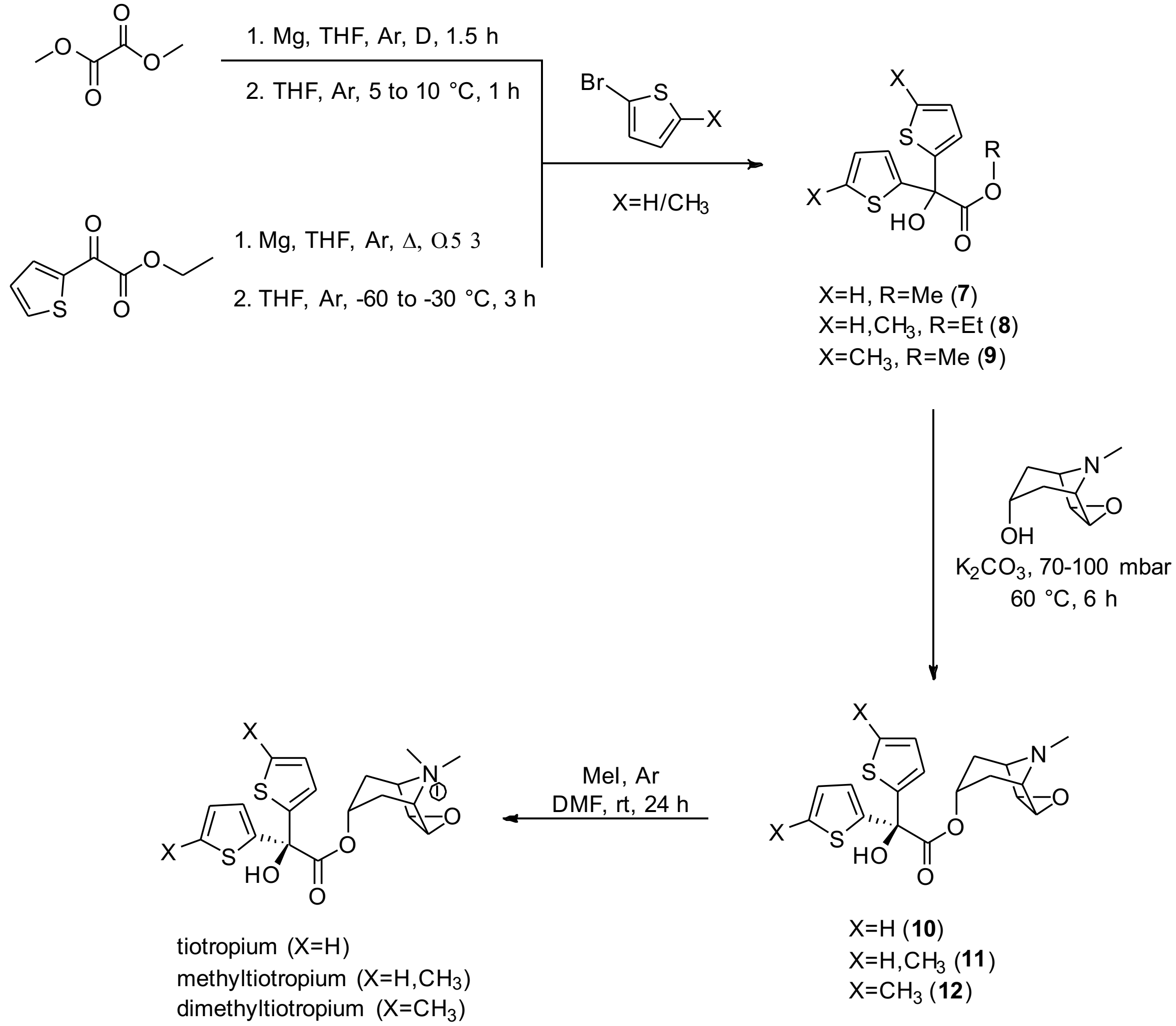

Methyl 2-hydroxy-2,2-di(thiophen-2-yl)acetate **(7)** (14)

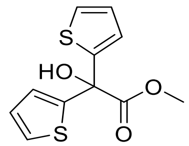

To a suspension of magnesium turnings (83.1 mg, 3.46 mmol) in dry THF (2 mL) were added 250 µl of a 2-bromothiophene solution (417 mg 2-bromothiophene (2.56 mmol) in dry THF (1 mL)) under Ar-atmosphere. The remaining 750 µl of the solution were added dropwise. The reaction mixture was stirred under reflux conditions for 1.5 h and then allowed to cool to room temperature. The solution was added dropwise to a solution of oxalic acid dimethylester (146 mg, 1.23 mmol) in dry THF (20 mL) under Ar-

atmosphere, while the reaction temperature was maintained at a maximum of 5 – 10 °C. The reaction was stirred at 5 – 10 °C for 1h, followed by the addition of cold saturated, aqueous NH_4Cl solution. The suspension was stirred at room temperature for additional 30 min and then extracted three times with toluene. The combined organic layers were washed twice with water and once with saturated, aqueous NaCl solution and dried (Na_2SO_4). After evaporation, the crude residue was purified by column chromatography on silica gel (*n*-hexane/ethyl acetate, 60:1) to give **7** (122 mg, 48%) as a pale yellow solid. Mp: 96 – 98.5 °C; IR (NaCl): 3478, 3106, 3092, 2961, 1722, 1434, 1266, 1233, 1129, 1090, 1044, 832 cm^{-1}; ^{1}H-NMR (600 MHz, $CDCl_3$): δ (ppm) = 7.29 (dd, *J* = 5.1, 1.2 Hz, 2H), 7.17 (dd, *J* = 3.6, 1.2 Hz, 2H), 6.98 (dd, *J* = 5.1, 3.6 Hz, 2H), 4.68 (s, 1H), 3.90 (s, 3H); ^{13}C-NMR (91 MHz, $CDCl_3$): δ (ppm) = 173.0, 145.8, 126.9, 126.2, 126.1, 76.7, 54.3; HPLC (254 nm, system A) : t_R= 18.8 min; APCI-MS: 236.9 $[(M+H)-H_20]^+$.

Ethyl 2-hydroxy-2-(5-methylthiophen-2-yl)-2-(thiophen-2-yl)acetate **(8)**

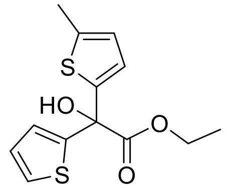

To a suspension of magnesium turnings (113 mg, 4.72 mmol) in dry THF (2 mL) were added 250 µl of a solution of the 5-methyl-2-bromothiophene (103 mg (582 µmol), dissolved in dry THF (1 mL)) under Ar-atmosphere. The remaining 750 µl of the solution were added dropwise. The reaction mixture was stirred under reflux conditions for 1.5 h and then allowed to cool to room temperature. The solution was added dropwise to a solution of ethyl 2-oxo-2-(thiophene-2-yl)acetate(15) (75.0 mg, 407 µmol) in dry THF (4 mL) under Ar-atmosphere, while the reaction temperature was maintained at a maximum of -55 °C. The reaction was stirred at -55 °C for 1h hour and then allowed to warm to -30 °C over two hours, followed by the addition of cold saturated, aqueous NH_4Cl solution. The suspension was stirred at room temperature for additional 30 min and then extracted three times with chloroform. The combined organic layers were washed twice with water and once with saturated, aqueous NaCl solution and dried (Na_2SO_4). After evaporation, the crude residue was purified by column chromatography on silica gel (*n*-hexane/ethyl acetate, 12:1) to give **8** (15.7 mg, 14%) as a pale yellow solid. Mp: 79 – 81 °C; IR (NaCl): 3481, 2983, 1730, 1643, 1444, 1254, 1227, 1128, 1043, 857 cm^{-1}; ^{1}H-NMR (360 MHz, $CDCl_3$): δ (ppm) = 7.27 (dd, *J* = 5.1, 1.3 Hz, 1H), 7.18 (dd, *J* = 3.6, 1.3 Hz, 1H), 6.97 (dd, *J* = 5.1, 3.6 Hz, 1H), 6.92 (d, *J* = 3.6 Hz, 1H), 6.61 (ddd, *J* = 3.3, 2.1, 1.0 Hz, 1H), 4.63 (s, 1H), 4.34 (q, *J* = 7.1 Hz, 2H), 2.44 (d, *J* = 1.0 Hz, 3H), 1.33 (t, *J* = 7.1 Hz, 3H); ^{13}C-NMR (151 MHz, $CDCl_3$): δ (ppm) = 172.4, 145.9, 143.1, 140.6, 126.7, 125.9, 125.8, 125.7, 124.8, 76.4, 63.6, 15.3, 14.0; HPLC (254 nm, system A): t_R= 20.0 min; APCI-MS: 265.0 $[(M+H)-H_20]^+$.

Methyl 2-hydroxy-2,2-bis(5-methylthiophen-2-yl)acetate **(9)** (16)

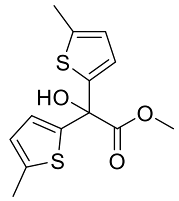

To a suspension of magnesium turnings (202 mg, 8.42 mmol) in dry THF (2 mL) 250 µl of a 2-bromo-5-methylthiophene-solution (145 mg 2-bromo-5-methylthiophene (819 µmol) in dry THF (1 mL)) were added under Ar-atmosphere. The remaining 750 µl of the solution were added dropwise. The reaction mixture was stirred under reflux conditions for 1.5 h and then allowed to cool to room temperature. The solution was added dropwise to a solution of oxalic acid dimethylester (43.2 mg (366 µmol) in dry THF (10 mL)) under Ar-atmosphere, while the reaction temperature was maintained at a maximum of 5 – 10 °C. The reaction was stirred at 5 – 10 °C for 1 h hour, followed by the addition of cold saturated, aqueous NH_4Cl solution. The suspension was stirred at room temperature for additional 30 min and then extracted three times with toluene. The combined organic layers were washed twice with water and once with saturated, aqueous NaCl solution and dried (Na_2SO_4). After evaporation, the crude residue was purified by column chromatography on silica gel (*n*-hexane/ethyl acetate, 60:1) to give **9** (9.5 mg, 9%) as a yellow semi solid substance. IR (NaCl): 3481, 2952, 2921, 1736, 1659, 1446, 1226, 1053, 993 cm^{-1}; ^{1}H-NMR (360 MHz, $CDCl_3$): δ (ppm) = 6.94 – 6.88 (m, 2H), 6.64 – 6.58 (m, 2H), 4.48 (s, 1H), 3.88 (s, 3H), 2.44 (d, J = 0.8 Hz, 6H); ^{13}C-NMR (151 MHz, $CDCl_3$): δ (ppm) = 173.2, 143.1, 140.8, 126.1, 125.0, 76.6, 54.2, 15.4; HPLC (254 nm system A): t_R= 20.2 min; APCI-MS: 265.0 $[(M+H)-H_20]^+$.

(2R,4S,7R)-9-Methyl-3-oxa-9-azatricyclo[3.3.1.0^{2,4}]nonan-7-yl 2-hydroxy-2,2-di(thiophen-2-yl)acetate ***(10)*** (14)

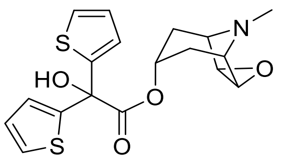

To a solution of scopine(14) (19.4 mg, 0.13 mmol) in dry DMF (4 mL) was added K_2CO_3 (17.9 mg, 131 µmol) under Ar-atmosphere and the suspension was stirred at room temperature for 1 h. Subsequently, a solution of compound **7** (20.6 mg, 81.0 µmol) in dry DMF (1 mL) and additional K_2CO_3 (9.0 mg, 65.7 µmol) were added, before the mixture was stirred under reduced pressure (70 – 100 mbar) at 65 °C for 8 h. The reaction was allowed to cool to room temperature before CH_2Cl_2 and water were added. The aqueous layer was extracted three times with CH_2Cl_2. The combined organic layers were washed twice with saturated, aqueous NaCl solution and dried (Na_2SO_4). After evaporation, the crude residue was purified by column chromatography on silica gel (CH_2Cl_2/MeOH, 60:1)

to give **10** (9.8 mg, 32%) as a pale yellow oil. IR (NaCl): 3487, 2924, 2850, 2251, 1731, 1432, 1232, 1128, 1044, 910, 854 cm^{-1}; ^{1}H-NMR (600 MHz, $CDCl_3$): δ 7.32 (dd, *J* = 5.1, 1.2 Hz, 2H), 7.13 (dd, *J* = 3.6, 1.2 Hz, 2H), 7.00 (dd, *J* = 5.1, 3.6 Hz, 2H), 5.15 – 5.12 (m, 1H), 4.76 (bs, 1H), 3.09 – 3.07 (m, *J* = 2.3 Hz, 2H), 3.01 – 2.98 (m, 2H), 2.48 (s, 3H), 2.18 – 2.12 (m, 2H), 1.64 – 1.58 (m, 2H); ^{13}C-NMR (151 MHz, $CDCl_3$): δ (ppm) = 171.6, 145.3, 127.0, 126.4, 126.3, 76.5, 70.7, 57.8, 56.4, 42.6, 31.1; HPLC (254 nm, system B): t_R= 15.0 min, purity: 98%; (254 nm, system C): t_R= 14.7 min, purity: 95%; HR-EI-MS: calculated 377.0756, found 377.0756 [M$^{\cdot}$]$^+$.

(2R,4S,7R)-9-Methyl-3-oxa-9-azatricyclo[3.3.1.0^{2,4}]nonan-7-yl 2-hydroxy-2-(5-methylthiophen-2-yl)-2-(thiophen-2-yl)acetate **(11)**

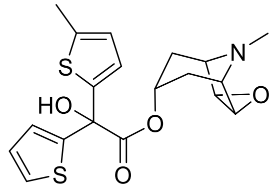

(*2R,4S,7R*)-9-Methyl-3-oxa-9-azatricyclo[3.3.1.0^{2,4}]nonan-7-yl 2-hydroxy-2-(5-methyl thiophen-2-yl)-2-(thiophen-2-yl)acetate **(11)** was prepared according to the protocol of compound **10**, using a solution of scopine(14) (14.4 mg, 92.8 µmol) in dry DMF (4 mL), K_2CO_3 (9.0 mg, 65.7 µmol) and a solution of compound **8** (8.0 mg, 29.8 mmol) in dry DMF (1 mL). Purification by column chromatography on silica gel (CH_2Cl_2/MeOH, 60:1) afforded **11** (5.7 mg, 54%) as a pale yellow oil. IR (NaCl): 3470, 1727, 1254, 1227, 1128, 1091, 1041, 1004, 800, 707 cm^{-1}; ^{1}H-NMR (600 MHz, Pyridine-d5): δ (ppm) = 7.48 – 7.44 (m, 2H), 7.23 – 7.19 (m, 1H), 7.07 (dd, *J* = 5.1, 3.6 Hz, 1H), 6.74 – 6.69 (m, 1H), 5.27 – 5.20 (m, 1H), 3.36 – 3.26 (m, 2H), 3.12 – 3.06 (m, 2H), 2.58 (s, 3H), 2.33 (d, *J* = 0.7 Hz, 3H), 2.26 – 2.15 (m, 2H), 1.75 – 1.66 (m, 2H); ^{13}C-NMR (151 MHz, Pyridine-d5): δ (ppm) = 171.5, 145.8, 140.3, 138.4, 127.1, 126.5, 126.3, 126.2, 125.2, 77.8, 69.4, 56.6, 44.1, 32.2, 30.4, 15.0; HPLC (254 nm, system A): t_R= 14.8 min, purity: 96%, (254 nm, system D): t_R= 13.7 min , purity: 98%; HR-EI-MS: calculated 391.0912, found 391.0911 [M$^{\cdot}$]$^+$.

(2R,4S,7R)-9-Methyl-3-oxa-9-azatricyclo[3.3.1.0^{2,4}]nonan-7-yl 2-hydroxy-2,2-bis(5-methyl thiophen-2-yl)acetate **(12)**

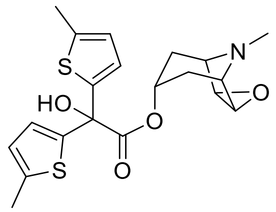

(*2R,4S,7R*)-9-Methyl-3-oxa-9-azatricyclo[3.3.1.0^{2,4}]nonan-7-yl 2-hydroxy-2,2-bis(5-methylthiophen-2-yl)acetate **(12)** was prepared according to the protocol of compound **10**, using a solution of scopine(14) (10.3 mg, 66.4 µmol) in dry DMF (4 mL), K_2CO_3 (12.0 mg, 87.6 µmol) and a solution of compound **9** (13.1 mg, 46.4 mmol) in dry DMF (1 mL). Purification by column chromatography on silica gel (CH_2Cl_2/MeOH, 60:1)

afforded **12** (3.0 mg, 18%) as pale yellow oil. IR (NaCl): 3468, 2972, 2912, 1719, 1654, 1225, 1040, 1003, 799, 704 cm^{-1}; ^{1}H-NMR (360 MHz, DMSO-d6): δ (ppm) = 6.81 (d, *J* = 3.5 Hz, 2H), 6.71 – 6.60 (m, 2H), 4.96 – 4.89 (m, 1H), 3.17 (s, 6H), 3.12 – 3.05 (m, 2H), 3.05 – 2.97 (m, 2H), 2.40 (m, 3H), 2.05 – 1.94 (m, 2H), 1.54 – 1.42 (m, 2H); ^{13}C-NMR (151 MHz, DMSO-d6): δ (ppm) = 170.1, 144.2, 139.2, 125.4, 124.7, 76.2, 68.5, 57.5, 56.0, 31.2, 14.9; HPLC (254 nm, system A): t_R= 15.4 min, purity: 96%, (254 nm system D): t_R= 13.9 min, purity: 95%; HR-EI-MS: calculated 405.1069, found 405.1065 $[M^{\cdot}]^+$.

(2R,4S,7R)-7-(2-Hydroxy-2,2-di(thiophen-2-yl)acetoxy)-9,9-dimethyl-3-oxa-9-azatricyclo[3.3.1.0^{2,4}]nonan-9-ium formate (tiotropium) **(14)**

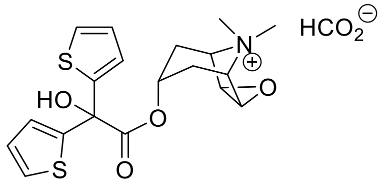

To a solution of compound **10** (4.0 mg, 11.0 µmol) in DMF (2 mL) was added under Ar-atmosphere methyliodide (5.00 µl, 80.3 µmol). The reaction was stirred at room temperature for 24 h, subsequently, additional methyliodide (5.00 µl, 80.3 µmol) was added and the reaction mixture was stirred at room temperature for additional 16 h. After evaporation, the crude residue was purified by preparative HPLC (column 2, eluent: CH_3OH in H_2O + 0.1% HCO_2H, (0 – 3 min 10%, 3 - 18 min, 10 – 60%) to afford the formate salt of **tiotropium** (2.0 mg, 42%) as a pale yellow solid. IR (NaCl): 2989, 1722, 1236, 1066, 944, 668 cm^{-1}; ^{1}H-NMR (600 MHz, D_2O): δ (ppm) = 8.75 (bs, 1H), 7.53 (d, *J* = 4.3 Hz, 2H), 7.22 (d, *J* = 2.7 Hz, 2H), 7.10 (dd, *J* = 4.9, 3.8 Hz, 2H), 5.31 – 5.25 (m, 1H), 4.09 – 4.04 (m, 2H), 3.44 – 3.40 (m, 2H), 3.32 (s, 3H), 3.07 (s, 3H), 2.84 – 2.76 (m, 2H), 2.11 – 2.04 (m, 2H); ^{13}C-NMR (91 MHz, D_2O): δ (ppm) = 180.5, 171.9, 144.8, 127.8, 127.7, 127.6, 79.5, 66.0, 65.2, 54.5, 28.8, 13.8; HPLC (254 nm, system A): t_R= 13.9 min, purity: 96%, (254 nm, system D): t_R= 13.9 min, purity: 95%; HR-ESI-MS: calculated 392.0985, found 392.0986 $[M]^+$.

(2R,4S,7R)-7-(2-Hydroxy-2-(5-methylthiophen-2-yl)-2-(thiophen-2-yl)acetoxy)-9,9-dimethyl-3-oxa-9-azatricyclo[3.3.1.0^{2,4}]nonan-9-ium formate **(methyltiotropium)**

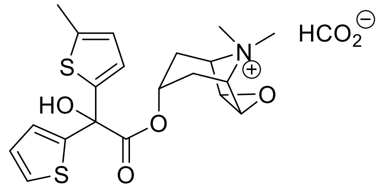

To a solution of compound **11** (3.5 mg, 8.94 µmol) in DMF (2 mL) was added methyliodide (10.0 µl, 161 µmol) under Ar-atmosphere. The reaction was stirred at room temperature for 24 h, subsequently, additional methyliodide (5.00 µl, 80.3 µmol) was added and the reaction mixture was stirred at room temperature for additional 16 h. After evaporation, the crude residue was purified by preparative HPLC (column 2, eluent: CH_3OH in H_2O + 0.1% HCO_2H, (0 – 3 min 10%, 3 - 18 min, 10 – 60%)) to afford the formate salt of **methyltiotropium** (3.2 mg, 78%) as a white solid. IR (NaCl): 3315, 3060,

2967, 1741, 1610, 1434, 1231, 1125, 1071, 999, 921, 859, 754, 709 cm^{-1}; ^{1}H-NMR (600 MHz, D_2O): δ (ppm) = 8.45 (s, 1H), 7.52 (dd, *J* = 5.1, 1.1 Hz, 1H), 7.21 (dd, *J* = 3.6, 1.1 Hz, 1H), 7.10 (dd, *J* = 5.0, 3.7 Hz, 1H), 6.98 (d, *J* = 3.6 Hz, 1H), 6.80 – 6.74 (m, 1H), 5.29 – 5.24 (m, 1H), 4.09 – 4.03 (m, 2H), 3.48 – 3.39 (m, 2H), 3.32 (s, 3H), 3.07 (s, 3H), 2.86 – 2.75 (m, 3H), 2.48 – 2.43 (m, 2H), 2.14 – 2.01 (m, 2H); ^{13}C-NMR (151 MHz, D_2O): δ (ppm) = 178.4, 171.6, 144.8, 142.0, 137.3, 127.8, 127.7, 127.7, 127.6, 125.7, 81.2, 65.9, 65.1, 54.6, 47.9, 28.8, 14.8; HPLC (254 nm, system A): t_R= 15.0 min, purity: 96%, (254 nm, system D): t_R= 14.1 min, purity: 95%; HR-ESI-MS: calculated 406.1141, found 406.1147 $[M]^+$.

(2R,4S,7R)-7-(2-Hydroxy-2,2-bis(5-methylthiophen-2-yl)acetoxy)-9,9-dimethyl-3-oxa-9-azatricyclo[3.3.1.0^{2,4}]nonan-9-ium formate **(dimethyltiotropium)**

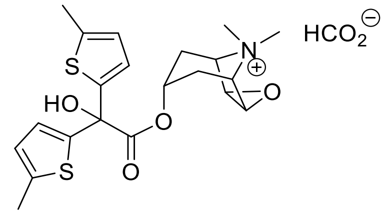

To a solution of compound **12** (5.0 mg, 12.0 μmol) in DMF (2 mL) was added methyliodide (10.0 μl, 161 μmol) under Ar-atmosphere. The reaction was stirred at room temperature for 16 h. After evaporation, the crude residue was purified by preparative HPLC (column 1, eluent: CH_3CN in H_2O + 0.1% HCO_2H (0 – 3 min 5%, 3 – 18 min 5 – 45 %)) to afford the formate salt of **dimethyltiotropium** (4.1 mg, 75%) as a white solid. IR (NaCl): 2828, 1653, 1595, 1457, 1354, 997, 772 cm^{-1}; ^{1}H-NMR (600 MHz, D_2O): δ (ppm) = 8.46 (bs, 1H), 6.97 (d, *J* = 3.5 Hz, 2H), 6.81 – 6.69 (m, 2H), 5.30 – 5.20 (m, 1H), 4.09 – 4.01 (m, 2H), 3.46 – 3.44 (m, 2H), 3.33 (s, 3H), 3.07 (s, 3H), 2.86 – 2.75 (m, 2H), 2.46 (s, 6H), 2.12 – 2.02 (m, 2H); ^{13}C-NMR (151 MHz, D_2O): δ (ppm) = 171.3, 142.7, 141.9, 127.6, 125.7, 77.4, 65.9, 65.0, 57.1, 54.6, 47.9, 28.8, 14.8 (H**C**O_2^- was not determined); HPLC (254 nm, system A) : t_R= 15.9 min, purity: 99%, (254 nm, system D): t_R= 14.5 min, purity: 98%; HR-ESI-MS: calculated 420.1297, found 420.1305 $[M]^+$.

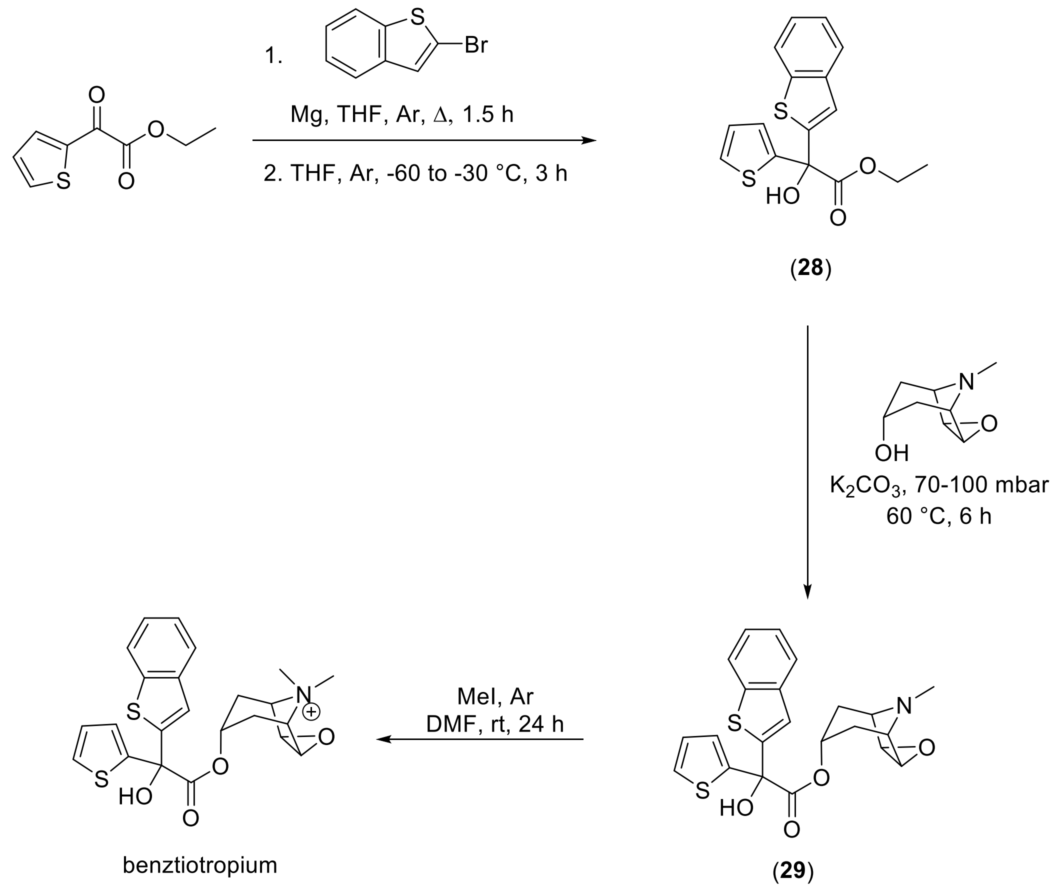

Ethyl-2-(benzo[b]thiophen-2-yl)-2-hydroxy-2-(thiophen-2-yl)acetat **(28)**

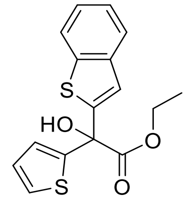

To a suspension of magnesium turnings (192 mg, 8.00 mmol) in dry THF (2 mL) were added 250 µl of a solution of the 2-bromobenzo[b]thiophene (200 mg, 0.94 mmol, dissolved in dry THF (1 mL)) under Ar-atmosphere. The remaining 750 µl of the solution were added dropwise. The reaction mixture was stirred under reflux conditions for 1.5 h and then allowed to cool to room temperature. The solution was added dropwise to a solution of ethyl 2-oxo-2-(thiophene-2-yl)acetate(15) (109 mg, 0.59 mmol) in dry THF (4 mL) under Ar-atmosphere, while the reaction temperature was maintained at a maximum of -55 °C. The reaction was stirred at -55 °C for 1h hour and then allowed to warm to -30 °C over two hours, followed by the addition of cold saturated, aqueous NH_4Cl solution. The suspension was stirred at room temperature for additional 30 min and then extracted three times with chloroform. The combined organic layers were washed twice with water and once with saturated, aqueous NaCl solution and dried (Na_2SO_4). After evaporation, the crude residue was purified by column chromatography on silica gel (*n*-

hexane/ethyl acetate, 12:1) to give **28** (143 mg, 76%) as a pale yellow solid. Mp: 90 – 91 °C; IR (NaCl): 3470, 2981, 1730, 1435, 1248, 1232, 1116, 1011, 855, 749, 707 cm^{-1}; ^{1}H-NMR (600 MHz, $CDCl_3$): δ (ppm) = 7.81 – 7.77 (m, 1H), 7.73 – 7.69 (m, 1H), 7.39 (s, 1H), 7.36 – 7.29 (m, 3H), 7.27 – 7.23 (m, 1H), 7.00 (dd, *J* = 5.0, 3.7 Hz, 1H), 4.80 (bs, 1H), 4.38 (qq, *J* = 10.7, 7.1 Hz, 2H), 1.35 (t, *J* = 7.1 Hz, 3H); ^{13}C-NMR (151 MHz, $CDCl_3$): δ (ppm) = 171.97, 146.39, 145.10, 139.92, 139.23, 126.81, 126.10, 126.04, 124.64, 124.34, 123.92, 122.72, 122.25, 76.73, 63.93, 14.00; HPLC (254 nm, system A): t_R= 21.0 min; ESI-MS: 341.3 $[M+Na]^+$.

9-Methyl-3-oxa-9-azatricyclo[3.3.1.0^{2,4}]nonan-7-yl-2-(benzo[b]thiophen-2-yl)-2-hydroxy-2-(thiophen-2-yl)acetat **(29)**

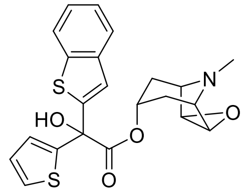

9-Methyl-3-oxa-9-azatricyclo[3.3.1.0^{2,4}]nonan-7-yl-2-(benzo[b]thiophen-2-yl)-2-hydroxy-2-(thiophen-2-yl) acetat **(29)** was prepared according to the protocol of compound **10**, using a solution of scopine(14) (22.0 mg, 0.14 mmol) in dry DMF (4 mL), K_2CO_3 (24.0 mg, 0.17 mmol) and a solution of compound 2**8** (22.8 mg, 0.07 mmol) in dry DMF (1 mL). Purification by column chromatography on silica gel (CH_2Cl_2/MeOH, 60:1) afforded **29** (6.6 mg, 21%) as a pale yellow oil. IR (NaCl): 2934, 1734, 1507, 1490, 1259, 1231, 1123, 1044, 972, 854, 704 cm^{-1}; ^{1}H-NMR (600 MHz, DMSO-d6): δ (ppm) = 7.96 – 7.89 (m, 1H), 7.86 – 7.80 (m, 1H), 7.53 (dd, *J* = 5.1, 1.2 Hz, 1H), 7.37 (s, 1H), 7.37 – 7.31 (m, 2H), 7.14 (dd, *J* = 3.6, 1.2 Hz, 1H), 7.02 (dd, *J* = 5.1, 3.6 Hz, 1H), 5.02 – 4.95 (m, 1H), 3.53 – 3.48 (m, 2H), 3.07 – 2.99 (m, 2H), 2.34 (s, 3H), 2.05 – 1.94 (m, 2H), 1.55 – 1.47 (m, 2H); ^{13}C-NMR (151 MHz, DMSO-d6): δ (ppm) = 169.76, 147.67, 146.22, 138.76, 126.69, 126.20, 125.85, 125.30, 124.60, 124.41, 124.37, 123.92, 122.28, 76.61, 68.92, 57.50, 55.89, 43.02, 31.16; HPLC (254 nm, system A): t_R= 16.1 min; ESI-MS: 428.4 $[M+H]^+$.

7-[2-(Benzo[b]thiophen-2-yl)-2-hydroxy-2-(thiophen-2-yl)acetoxy]-9,9-dimethyl-3-oxa-9 azoniatricyclo[3.3.1.0^{2,4}]nonan-formiat **(benztiotropium)**

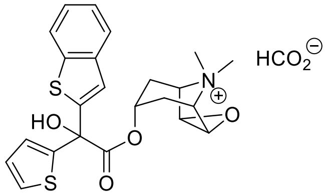

To a solution of compound **29** (6.0 mg, 14.0 µmol) in DMF (2 mL) was added methyliodide (10.0 µl, 0.16 mmol) under Ar-atmosphere. The reaction was stirred at room temperature for 16 h, subsequently, additional methyliodide (5.00 µl, 80.3 µmol) was added and the reaction mixture was stirred at room temperature for additional 16 h. After evaporation, the crude residue was purified by preparative HPLC (column 1, eluent:

CH_3CN in H_2O + 0.1% HCO_2H, (0 – 3 min 10%, 3 - 18 min, 10 – 50%)) to afford the formate salt of **benztiotropium** (5.0 mg, 74%) as a white solid. IR (NaCl): 3056, 2827, 1734, 1600, 1354, 1230, 1203, 1070, 921, 859, 753 cm^{-1}; ^{1}H-NMR (360 MHz, DMSO-d6): δ (ppm) = 7.97 – 7.88 (m, 1H), 7.87 – 7.80 (m, 1H), 7.55 (dd, *J* = 5.1, 1.2 Hz, 1H), 7.45 (s, 1H), 7.41 – 7.31 (m, 2H), 7.20 (dd, *J* = 3.6, 1.2 Hz, 1H), 7.03 (dd, *J* = 5.1, 3.6 Hz, 1H), 5.20 – 5.13 (m, 1H), 4.14 – 4.08 (m, 2H), 3.61 – 3.55 (m, 2H), 3.22 (s, 3H), 3.03 (s, 3H), 2.76 – 2.62 (m, 2H), 2.05 – 1.89 (m, 2H); ^{13}C-NMR (91 MHz, DMSO-d6): δ (ppm) = 169.55, 147.64, 146.30, 138.87, 138.79, 126.75, 126.25, 125.83, 124.62, 124.39, 123.93, 122.25, 122.19, 76.64, 64.54, 63.75, 55.61, 54.86, 53.60, 28.29; HPLC (254 nm, system A): t_R= 16.5 min, purity: 96%, (254 nm, system D): t_R= 15.0 min, purity: 97%; HR-ESI-MS: calculated 442.1147, found 442.1147 $[M]^+$.

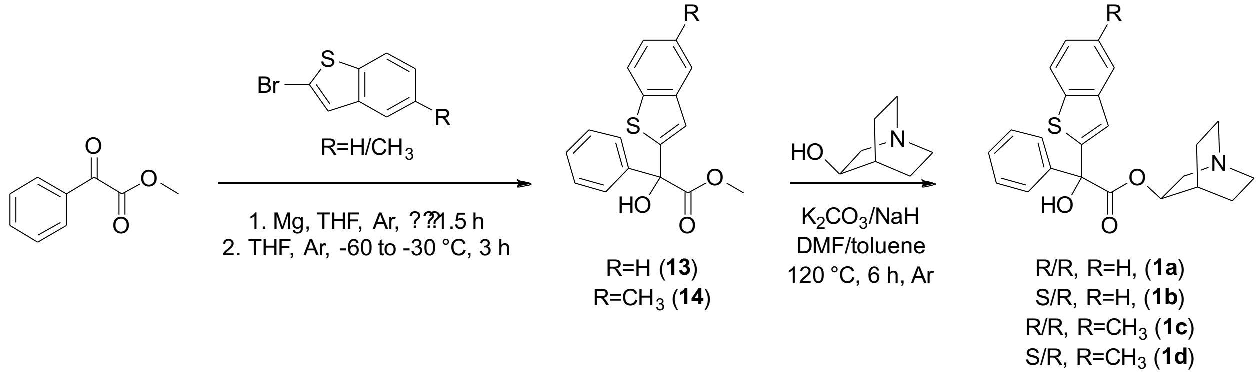

Methyl 2-(benzo[b]thiophen-2-yl)-2-hydroxy-2-phenylacetate **(13)**

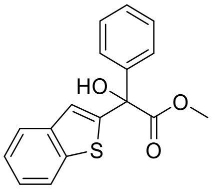

Methyl 2-(benzo[b]thiophen-2-yl)-2-hydroxy-2-phenylacetate **(13)** was prepared according to the protocol of compound **8**, using a suspension of magnesium turnings (211 mg, 8.80 mmol) in dry THF (2 mL), a solution of 2-bromobenzo[b]thiophene (220 mg, 1.03 mmol) in dry THF (1 mL) and a solution of phenylglyoxylic acid methyl ester (143 mg, 871 µmol). Purification by flash chromatography (*n*-hexane/ethyl acetate, 15:1) afforded **13** (171 mg, 66%) as a white solid. Mp: 133 – 147 °C; IR (NaCl): 3482, 3059, 2950, 1732, 1435, 1248, 1125, 1066, 977, 834, 747 cm^{-1}; ^{1}H-NMR (360 MHz, $CDCl_3$): δ (ppm) = 7.82 – 7.77 (m, 1H), 7.74 – 7.69 (m, 1H), 7.59 – 7.53 (m, 2H), 7.40 – 7.27 (m, 6H), 4.49 (s, 1H), 3.91 (s, 3H); ^{13}C-NMR (91 MHz, $CDCl_3$): δ (ppm) = 173.5, 146.6, 140.8, 140.0, 139.3, 128.6, 128.3, 126.7, 124.6, 124.4, 123.8, 123.1, 122.2, 79.1, 54.0; HPLC (254 nm, system A): t_R= 20.8 min; APCI-MS: 281.2 $[(M+H)-H_2O]^+$.

Methyl 2-hydroxy-2-(5-methylbenzo[b]thiophen-2-yl)-2-phenylacetate **(14)**

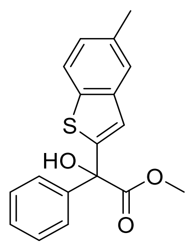

Methyl 2-hydroxy-2-(5-methylbenzo[b]thiophen-2-yl)-2-phenylacetate **(14)** was prepared according to the protocol of compound **8**, using a suspension of magnesium turnings (250 mg, 10.3 mmol) in dry THF (2 mL), a solution of 2-bromo-5-methylbenzo[b]thiophene(17) (242 mg, 1.07 mmol) in dry THF (1 mL) and a solution of phenylglyoxylic acid methyl ester (500 mg, 3.05 mmol) in dry THF (5 mL), allowing a reaction time of 2 h with an increase in temperature from -60 °C to -30 °C. Flash chromatography over silica gel (*n*-hexane/ethyl acetate, 6:1) afforded **14** (95.6 mg, 29%) as a pale yellow solid. Mp: 144 – 150 °C; IR (NaCl): 2920, 1742, 1451, 1259, 1111, 1067, 702 cm^{-1}. ^{1}H-NMR (600 MHz, $CDCl_3$): δ (ppm) = 7.70 (d, *J* = 8.2 Hz, 1H), 7.60 – 7.57 (m, 2H), 7.46 – 7.45 (m, 1H), 7.40 – 7.33 (m, 3H), 7.14 (dd, *J* = 8.3, 1.4 Hz, 1H), 7.11 (s, 1H), 4.22 (s, 1H), 3.85 (s, 3H), 2.37 (s, 3H); ^{13}C-NMR (151 MHz, $CDCl_3$): δ (ppm) = 174.7, 140.3, 138.0, 137.5, 136.1, 133.9, 128.3, 128.3, 126.8, 126.3, 126.2, 123.9, 122.3, 78.9, 53.7, 21.6; HPLC (254 nm, system A): t_R= 21.0 min; ESI-MS: 335.5 $[M+Na]^+$.

(1R,3S,4R)-Quinuclidin-3-yl 2-(benzo[b]thiophen-2-yl)-2-hydroxy-2-phenylacetate **(1a/1b)**

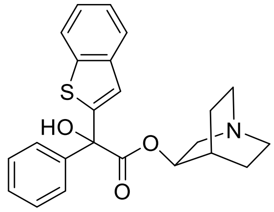

To a solution of (*R)*-(-)-3-quinuclidinol (86.3 mg, 678 µmol) in dry toluene (4 mL) was added NaH (60% suspension, 41.6 mg, 1.04 mmol) and a solution of compound **13** (100 mg, 335 mmol) in dry toluene under Ar-atmosphere. The mixture was stirred under reflux conditions for 2 h. After the mixture was allowed to cool to room temperature water was added. The aqueous phase was extracted three times with DCM and the combined organic layers were washed twice with saturated, aqueous NaCl-solution and dried (Na_2SO_4). After evaporation, the crude residue was purified by column chromatography on silica gel (CH_2Cl_2/MeOH/NH_3 aqu. 25%, 10:1:0.015) to obtain a mixture of the

diastereomers **1a**/**1b** (87.5 mg, 66%) as a pale yellow oil. Diastereomers were afterwards separated by flashchromatography on silica gel (chloroform/CH_3CN/triethylamine, 20:1:0.02). IR (NaCl): 3059, 2950, 1734, 1450, 1435, 1237, 1139, 1058, 1006, 728 cm^{-1}; ^1H-NMR **1a** (600 MHz, $CDCl_3$): δ (ppm) = 7.83 – 7.79 (m, 1H), 7.76 – 7.72 (m, 1H), 7.59 – 7.54 (m, 2H), 7.39 – 7.30 (m, 6H), 5.04 – 5.00 (m, 1H), 4.69 (s, 1H), 3.25 (ddd, J = 14.8, 8.1, 2.0 Hz, 1H), 2.83 – 2.66 (m, 5H), 2.05 – 2.01 (m, 1H), 1.72 – 1.64, 1.59 – 1.50, 1.46 – 1.40, 1.31 – 1.24 (4xm, each 1H); ^1H-NMR **1b** (600 MHz, $CDCl_3$): δ (ppm) = 7.82 – 7.79 (m, 1H), 7.73 (dd, J = 7.0, 1.5 Hz, 1H), 7.62 – 7.57 (m, 2H), 7.43 – 7.31 (m, 6H), 5.05 – 5.00 (m, 1H), 3.33 – 3.23 (m, 1H), 2.89 – 2.72 (m, 4H), 2.66 (s, 1H), 2.19 – 2.14 (m, 1H), 1.78 – 1.70, 1.69 – 1.62, 1.61 – 1.54, 1.39 – 1.34 (4xm, each 1H);); ^{13}C-NMR **1a** (91 MHz, $CDCl_3$): δ 171.0, 140.7, 139.9, 139.1, 139.0, 128.8, 128.4, 126.6, 124.9, 124.6, 124.0, 123.4, 122.3, 79.1, 71.9, 52.0, 46.6, 45.2, 24.5, 22.7, 18.0; ^{13}C-NMR **1b** (91 MHz, $CDCl_3$): δ (ppm) = 172.6, 146.3, 140.7, 140.0, 139.1, 128.8, 128.5, 126.4, 124.7, 124.5, 123.9, 123.3, 122.2, 79.0, 72.5, 53.9, 46.6, 45.7, 24.6, 22.5, 18.3; ; HPLC diastereomeric mixture (220 nm, system A): t_R= 17.0 min, purity > 99%, (220 nm, system D): t_R= 15.1 min, purity: 97%; **1a**: (254 nm, system A): t_R= 17.0 min, purity = 95%, (254 nm, system D): t_R= 14.7 min, purity = 95%; **1b**: (254 nm, system A): t_R= 16.1 min, purity = 95%, (254 nm, system D): t_R= 14.7 min, purity = 95%; HR-EI-MS: calculated 393.1399, found 393.1398 $[M^{\cdot}]^+$. The ratio of the diastereomers was determined via HPLC (column: EC 250/4 Nucleodur C18 HTec, 5µm, 1 mL/min, solvent: 5% CH_3CN for 3 min, 5% → 43% CH_3CN in 19 min, 43% → 95% CH_3CN in 3 min, 95% CH_3CN for 6 min); **1a**: *dr* = 90%; **1b**: *dr* = 88%. The assignement of the configuration of **1a** and **1b** was done by analytical investigation of the carboxylic acids, obtained after saponification of **1a** and **1b**. Those were investigated by CD spectroscopy and compared to calculated CD spectra using Gaussian 09. With respect to the fact that CD spectra are influenced by the solvent, we adopted solvent continuum reaction field.

(1R,3S,4R)-Quinuclidin-3-yl 2-hydroxy-2-(5-methylbenzo[b]thiophen-2-yl)-2-phenylacetate **(1c/1d)**

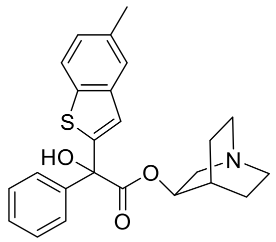

To a solution of (*R*)-(-)-3-quinuclidinol (59.3 mg, 466 µmol) in dry DMF (4 mL) was added K_2CO_3 (69.7 mg, 509 µmol) under Ar-atmosphere and the suspension was stirred at room temperature for 1 h. Subsequently, a solution of **14** (52.3 mg, 167 µmol), in dry DMF (1 mL) was added, before the reaction mixture was stirred at 110 °C for 8 h. After the mixture was allowed to cool to room temperature, CH_2Cl_2 and water were added and

the aqueous phase was extracted three times with CH_2Cl_2. The combined organic layers were washed twice with saturated, aqueous NaCl solution and dried (Na_2SO_4). After evaporation, the crude residue was purified by flash chromatography on silica gel (CH_2Cl_2/MeOH/NH_3 aqu. 25%, 10:1:0.01) to obtain a mixture of the diastereomers **1c**/**1d** (23.9 mg, 37%) as a pale yellow solid. Mp: 165 – 166.5 °C; IR (NaCl): 2947, 1732, 1668, 1447, 1236, 1085, 1066, 1012, 913, 793, 734 cm^{-1}; ^{1}H-NMR **1c** (600 MHz, $CDCl_3$): δ (ppm) = 7.71 (d, J = 8.2 Hz, 1H), 7.60 – 7.57 (m, 2H), 7.45 (dd, J = 1.7, 1.0 Hz, 1H), 7.42 – 7.34 (m, 3H), 7.25 (s, 1H), 7.14 (dd, J = 8.3, 1.6 Hz, 1H), 5.03 – 4.98 (m, 1H), 4.34 (s, 1H), 3.18 (ddd, J = 14.8, 8.2, 2.2 Hz, 1H), 2.76 – 2.61 (m, 3H), 2.52 – 2.47 (m, 1H), 2.45 – 2.39 (m, 1H), 2.35 (s, 3H), 1.95 – 1.89 (m, 1H), 1.67 – 1.60 (m, 1H), 1.54 – 1.47 (m, 1H), 1.21 – 1.08 (m, 2H); ^{1}H-NMR **1d** (600 MHz, $CDCl_3$): δ (ppm) = 7.71 (d, J = 8.3 Hz, 1H), 7.62 – 7.58 (m, 2H), 7.52 (s, 1H), 7.42 – 7.34 (m, 3H), 7.17 – 7.14 (m, 2H), 4.98 – 4.93 (m, 1H), 4.27 (s, 1H), 3.18 (ddd, J = 14.8, 8.1, 2.2 Hz, 1H), 2.75 – 2.64 (m, 2H), 2.60 (ddd, J = 22.8, 10.5, 4.9 Hz, 2H), 2.38 (s, 3H), 2.23 – 2.15 (m, 1H), 2.00 – 1.94 (m, 1H), 1.67 – 1.60 (m, 1H), 1.54 – 1.45 (m, 1H), 1.20 – 1.10 (m, 2H); ^{13}C-NMR (91 MHz, $CDCl_3$): δ (ppm) 173.9/173.8, 140.5/140.4, 138.0/138.0, 137.5/137.4, 136.2/135.9, 133.9/133.7, 128.4/128.4, 128.3/128.3, 126.8/126.8, 126.3/126.3, 126.2/126.1, 124.2/124.0, 122.4/122.3, 78.9/78.9, 74.1/74.0, 54.7/54.5, 47.0/46.8, 46.2, 30.9, 25.1/25.0, 24.0/23.9, 21.6/21.6, 19.0/18.9; HPLC **1c:** (220 nm, system A): t_R= 17.3 min, purity: 97%, (220 nm, system D): t_R= 15.0 min, purity: 97%; **1d**: (220 nm, system A): t_R= 17.1 min, purity: 98%, (220 nm, system D): t_R= 14.9 min, purity: 97%, HR-ESI-MS **1c**: calculated 408.1628, found 408.1627 $[M]^+$, HR-ESI-MS **1d**: calculated 408.1628, found 408.1631 $[M]^+$. The diastereomers were separated by preparative chiral HPLC (column: Chiralpak AS-H (1 cm x 25 cm, 5 µm), solvent: isocratic CH_3CN + 0.1% diethylamin, 3 mL/min). The ratio of the diastereomers was determined via NMR, **1c** *dr* = 60%**d**; **1d:** *dr* = 82%. The assignement of the configuration of **1c** and **1d** was done by NMR spectroscopy comparing diagnostic signals with those of **1a** and **1b**.

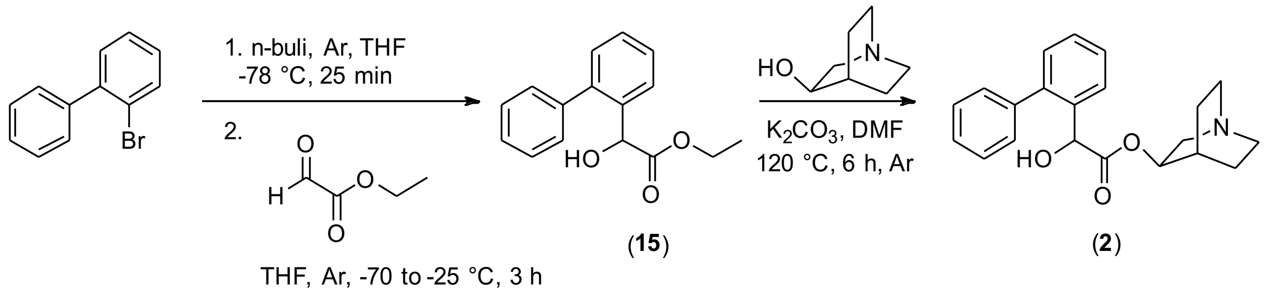

Ethyl 2-([1,1'-biphenyl]-2-yl)-2-hydroxyacetate **(15)** *(18)*

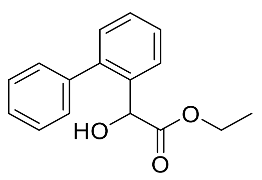

To a suspension of magnesium turnings (205 mg, 8.54 mmol) in dry THF (2 mL) were added 250 µl of a solution 2-bromobiphenyl (150 µl (0.86 mmol), dissolved in dry

THF (1 mL)) under Ar-atmosphere. The remaining 750 µl of the solution were added dropwise. The reaction mixture was stirred under reflux conditions for 1.5 h and then allowed to cool to room temperature. The solution was added dropwise to a solution glyoxylic acid ethyl ester (50% in toluene, 200 µl (98.0 µmol) in dry THF (4 mL)) under Ar-atmosphere, while the reaction temperature was maintained at a maximum of -55 °C. The reaction was stirred at -60 °C for 1h hour and then allowed to warm to -20 °C over three hours, followed by the addition of cold saturated, aqueous NH_4Cl solution. The suspension was stirred at room temperature for additional 30 min and then extracted three times with chloroform. The combined organic layers were washed twice with water and once with saturated, aqueous NaCl solution and dried (Na_2SO_4). After evaporation, the crude residue was purified by column chromatography on silica gel (*n*-hexane/ethyl acetate, 5:1) to give **15** (171 mg, 78%) as a pale yellow oil. IR (NaCl): 3489, 2982, 1735, 1481, 1217, 1184, 1071, 747, 704 cm^{-1}; ^{1}H-NMR (360 MHz, $CDCl_3$): δ (ppm) = 7.48 – 7.34 (m, 8H), 7.33 – 7.26 (m, 1H), 5.22 (d, *J* = 4.5 Hz, 1H), 4.28 – 3.99 (m, 2H), 3.42 (d, *J* = 4.9 Hz, 1H), 1.18 (t, *J* = 7.1 Hz, 3H); ^{13}C-NMR (91 MHz, $CDCl_3$): δ (ppm) = 174.4, 142.4, 140.5, 136.4, 130.6, 129.8, 128.5, 128.3, 128.1, 127.5, 127.0, 69.9, 62.2, 14.1; HPLC (254 nm, system A): t_R= 20.2 min; ESI-MS: 279.3 $[M+Na]^+$.

(1R,3S,4R)-Quinuclidin-3-yl 2-([1,1'-biphenyl]-2-yl)-2-hydroxyacetate **(2)**

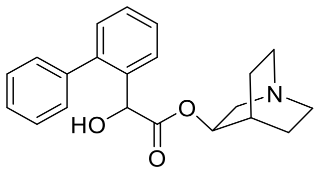

To a solution of (*R)*-(-)-3-quinuclidinol (36.8 mg, 289 µmol) in dry DMF (4 mL) was added K_2CO_3 (37.3 mg, 272 µmol) under Ar-atmosphere and the suspension was stirred at room temperature for 1 h. Subsequently, a solution of **15** (22.7 mg, 88.6 µmol), in dry DMF (1 mL) was added, before the reaction mixture was stirred at 110 °C for 8 h. After the mixture was allowed to cool to room temperature, CH_2Cl_2 and water were added and the aqueous phase was extracted three times with CH_2Cl_2. The combined organic layers were washed twice with saturated, aqueous NaCl solution and dried (Na_2SO_4). After evaporation, the crude residue was purified by preparative HPLC (column 1, eluent: CH_3CN in H_2O + 0.1% HCO_2H (0 – 3 min 5%, 3 – 15 min 5 – 45 %)) to give the formate salt of **2** (mixture of diastereomers, 3.0 mg, 10%) as a white solid. IR (NaCl): 2932, 2814, 1610, 1351, 1176, 1119, 989, 765 cm^{-1}; ^{1}H-NMR (600 MHz, DMSO): δ (ppm) = 8.32 (bs, 1H), 7.58 – 7.35 (m, 8H), 7.26 (t, *J* = 7.8 Hz, 1H), 5.13 (d, *J* = 6.3 Hz, 1H), 4.63 (s, 1H), 3.10 – 2.93 (m, 1H), 2.68 – 2.26 (m, 5H), 2.07 – 1.95 (m, 1H), 1.84 – 1.76 (m, 1H), 1.62 – 1.49 (m, 1H), 1.46 – 1.37 (m, 2H); ^{13}C-NMR (151 MHz, $CDCl_3$): δ (ppm) = 186.1, 174.1, 142.1/142.1, 140.3/140.3, 136.3/136.0, 130.5/130.5, 129.6/129.6, 128.5/128.4, 128.3/128.3, 128.1/128.0, 127.5/127.4, 126.6/126.5, 72.99, 69.53, 54.72, 46.91, 46.16, 29.74, 24.94, 23.97; HPLC (254 nm, system A): t_R= 15.4 min, purity: 95%, (254 nm, System D): t_R= 13.3 min, purity: 97%; HR-ESI-MS: calculated 338.1751, found 338.1754 $[M^{\cdot}]^+$.

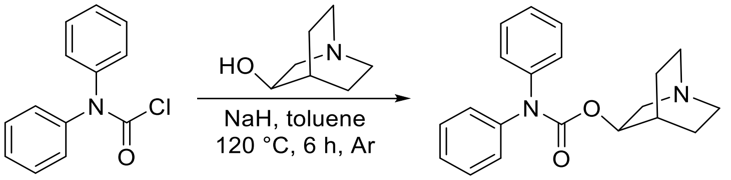

(3)

(1R,3S,4R)-Quinuclidin-3-yl diphenylcarbamate **(3)** *(25)*

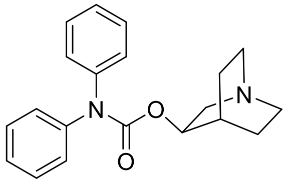

To a solution of *(R)*-(-)-3-quinuclidinol (96.0 mg, 75.6 µmol) in dry toluene (4 mL) was added under Ar-atmosphere at 0 °C NaH (60% suspension, 44.3 mg, 1.11 mmol). The mixture was stirred for 1 h at room temperature, before diphenyle carbamoylchloride (121 mg, 522 µmol) was added. The reaction was stirred under reflux conditions for 2 h. The mixture was allowed to cool to room temperature before chloroform and water were added and the aqueous phase was extracted three times with chloroform. The combined organic layers were washed twice with saturated, aqueous NaCl-solution and dried (Na_2SO_4). After evaporation, the crude residue was purified by column chromatography on silica gel (CH_2Cl_2/MeOH, 10:1) to give **3** (21.4 mg, 13%) as a pale brown oil. IR (NaCl): 3402, 2943, 1674, 1591, 1492, 1351, 1302, 1216, 1051, 765, 695 cm^{-1}; ^{1}H-NMR (360 MHz, $CDCl_3$): δ (ppm) = 7.39 – 7.30 (m, 6H), 7.29 – 7.17 (m, 4H), 4.90 – 4.83 (m, 1H), 3.34 – 3.16 (m, 1H), 2.86 – 2.61 (m, 5H), 2.07 – 1.99 (m, 1H), 1.74 – 1.62, 1.62 – 1.50, 1.50 – 1.39, 1.35 – 1.22 (4xm, each 1H); ^{13}C-NMR: (91 MHz, $CDCl_3$): δ (ppm) = 162.5, 154.2, 142.4, 129.2, 128.9, 126.8, 126.2, 72.1, 55.4, 47.1, 46.2, 25.1, 23.8, 19.3; HPLC (254 nm, System A): t_R= 15.9 min, (254 nm, system D): t_R= 14.1 min ; HR-ESI-MS: calculated 323.1754, found 323.1747 $[M^{\cdot}]^+$

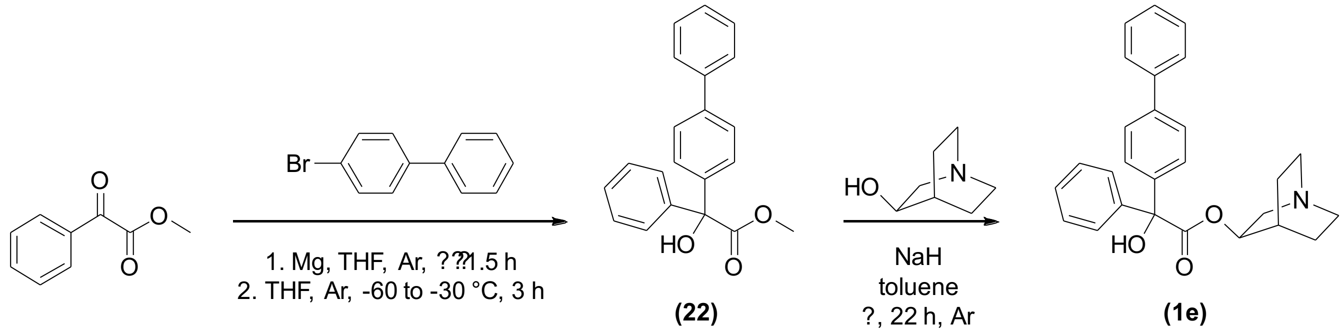

Methyl 2-([1,1'-biphenyl]-4-yl)-2-hydroxy-2-phenylacetate **(22)**

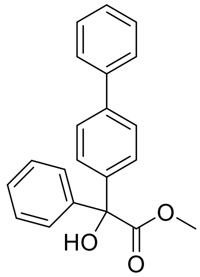

To a suspension of magnesium turnings (209 mg, 8.71 mmol) in dry THF (2 mL) were added 250 µl of a 4-bromobiphenyl solution (232 mg 4-bromobiphenyl (1.00 mmol) in dry THF (1 mL)) under Ar-atmosphere. The remaining 750 µl of the solution were added dropwise. The reaction mixture was stirred under reflux conditions for 1.5 h and then allowed to cool to room temperature. The solution was added dropwise to a solution of phenylglyoxalic acid methylester (138 mg, 0.84 mmol) in dry THF at -60 °C under Ar-atmosphere, while the reaction temperature was maintained at a maximum of -55 °C. The reaction was stirred at -60 °C for 1h, and was allowed to warm to -30 °C over 2h. Subsequently cold saturated, aqueous NH_4Cl solution was added and the suspension was extracted three times with DCM. The combined organic layers were washed twice with water and once with saturated, aqueous NaCl solution and dried (Na_2SO_4). After evaporation, the crude residue was purified by column chromatography on silica gel (*n*-hexane/ethyl acetate, 30:1 -> 15:1) to give **22** (136 mg, 51%) as a colorless solid. Mp: 112 – 116 °C; IR (NaCl): 3489, 3031, 2954, 1730, 1486, 1448, 1252, 1166, 1064, 1008, 840, 764 cm^{-1}; ^{1}H-NMR (600 MHz, $CDCl_3$): δ (ppm) = 7.61 – 7.58 (m, 2H), 7.58 – 7.55 (m, 2H), 7.50 – 7.48 (m, 2H), 7.47 – 7.41 (m, 4H), 7.40 – 7.32 (m, 4H), 4.21 (s, 1H), 3.89 (s, 3H); ^{13}C-NMR (151 MHz, $CDCl_3$): δ (ppm) = 174.93, 141.87, 140.95, 140.87, 140.55, 128.78, 128.19, 128.15, 127.81, 127.45, 127.34, 127.13, 126.85, 80.99, 53.64; HPLC (254 nm, system A) : t_R= 22.0 min; ESI-MS: 341.5 $[M+Na]^+$.

(R)-Quinuclidin-3-yl-2-([1,1'-biphenyl]-4-yl)-2-hydroxy-2-phenylacetate **(1e)**

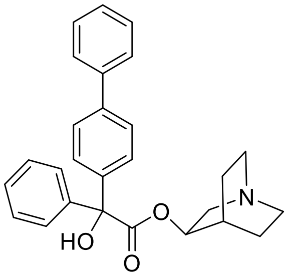

*(R)-*Quinuclidin-3-yl-2-([1,1'-biphenyl]-4-yl)-2-hydroxy-2-phenylacetat **(1e)** was prepared according to the protocol of compound **1a/1b**, using a solution of (*R)*-(-)-3-

quinuclidinol (72.2 mg, 0.57 mmol) in dry toluene (2 mL), NaH (60% solution (28.2 mg, 0.71 mmol) in dry toluene (1 mL)) and a solution of compound **22** (90.1 mg, 0.28 mmol) in dry toluene (1 mL) stirring the reaction mixture for 22 h under reflux conditions. Purification by preparative HPLC (column 1, CH_3CN /H_2O + 0.1% HCO_2H, gradient: 5% → 5% CH_3CN in 3 min, 5% → 50% CH_3CN in 18 min) obtained the formate salt of compound **1e** (mixture of distereomers) as a white solid (5.6 mg, 4 %). IR: (NaCl): 3341, 2954, 2930, 2567, 1735, 1660, 1627, 1488, 1450, 1235, 1015, 767 (cm^{-1}); ^{1}H-NMR (600 MHz, DMSO-d6): δ (ppm) = 7.69 – 7.63 (m, 4H), 7.46 (dd, J = 16.1, 8.0 Hz, 4H), 7.42 – 7.33 (m, 5H), 7.33 – 7.28 (m, 1H), 6.65 (s, 1H), 4.88 – 4.81 (m, 1H), 3.16 – 3.06 (m, 1H), 2.70 – 2.54 (m, 3H), 2.48 – 2.37 (m, 2H), 1.91 – 1.87 (m, 1H), 1.60 – 1.53, 1.51 – 1.44, 1.43 – 1.35, 1.23 – 1.14 (4xm, 1H each); ^{13}C-NMR (151 MHz, DMSO-d6): δ (ppm) = 172.46, 143.16/143.09, 142.39/142.32, 139.52, 139.09, 128.70, 127.59, 127,57, 127.53/127.48, 127.27, 126.87/126.83, 126.42, 125.80, 80.38, 72.54, 54.51/54.50, 46.50/46.49, 45.61, 24.75, 23.72/23.70, 18.84; HPLC: (220 nm, system A): t_R= 17.6 min, purity: >99%, (220 nm, system D): t_R= 15.7 min, purity: >99%; HR-ESI-MS: calculated 413.1991, found 413.19920 $[M]^+$.

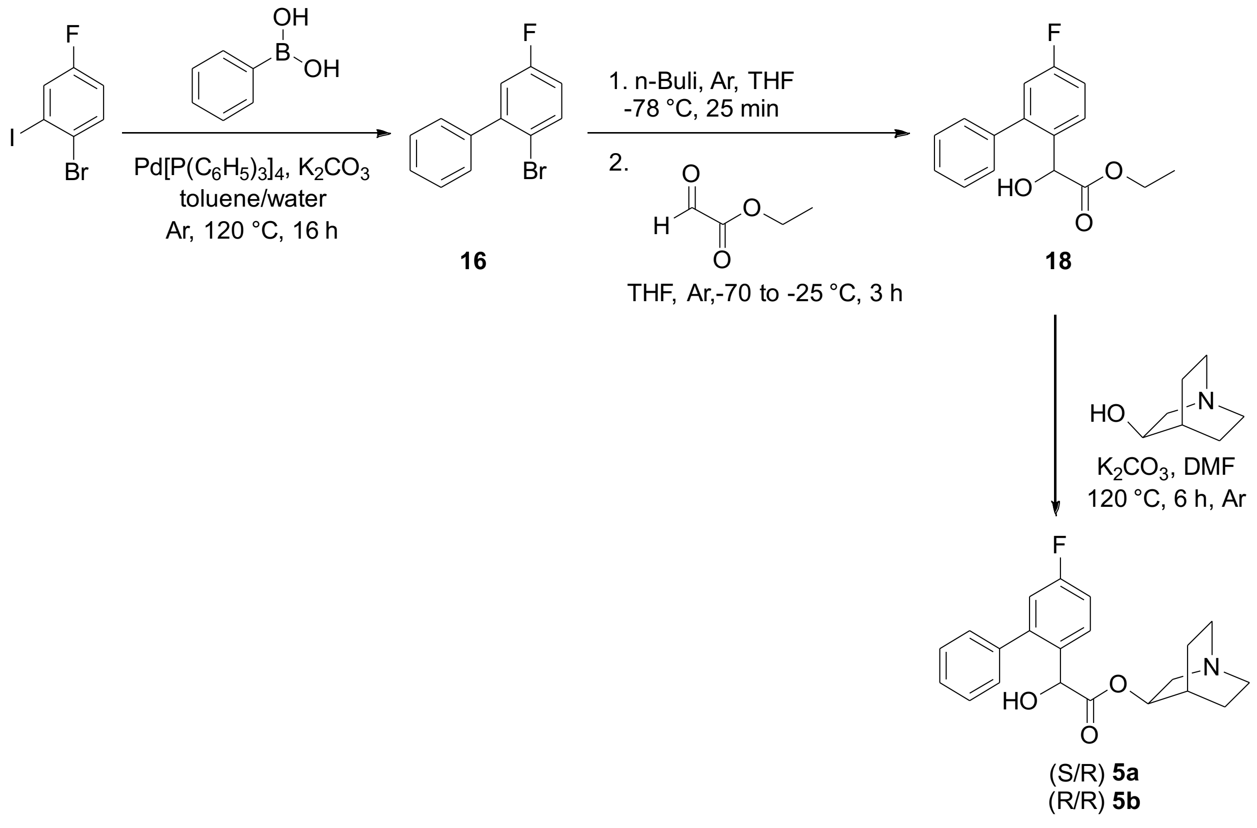

2-Bromo-5-fluoro-1,1'-biphenyl **(16)**

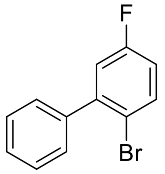

To a solution of 1-bromo-4-fluoro-2-iodobenzene (2.50 mL, 21.0 mmol) in dry toluene (20 mL) were added phenylboronic acid (810 mg, 6.64 mmol), tetrakistriphenylphosphin-palladium(0) (690 mg, 597 µmol) and an aqueous K_2CO_3 solution (3 M, 2 mL) under Ar-atmosphere. The reaction was stirred at 120 °C for 16 h. The mixture was allowed to cool to room temperature, followed by the addition of saturated, aqueous $NaHCO_3$ solution and the aqueous layer was extracted three times with ethyl acetate. The combined organic layers were washed once with saturated, aqueous NaCl solution and dried (Na_2SO_4). After evaporation, the crude residue was purified by column chromatography on silica gel (*n*-hexane) to give **16** (1.48 mg, 89%) as a colorless oil. IR (NaCl): 3085, 1581, 1456, 1253, 1210, 1185, 1014, 809, 699 cm^{-1}; ^{1}H-NMR (600 MHz, $CDCl_3$): δ (ppm) = 7.61 (dd, *J* = 8.8, 5.4 Hz, 1H), 7.46 – 7.37 (m, 5H), 7.06 (dd, *J* = 9.1, 3.1 Hz, 1H), 6.94 (ddd, *J* = 8.8, 7.9, 3.1 Hz, 1H); ^{13}C-NMR (151 MHz, $CDCl_3$): δ (ppm) = 161.9 (d, *J* = 249 Hz), 144.5 (d, *J* = 8.0 Hz), 140.3, 134.4 (d, *J* = 8.8 Hz), 129.3, 128.3, 128.2, 118.4 (d, *J* = 23 Hz), 117.0 (d, *J* = 3.6 Hz), 116.0 (d, *J* = 22.1 Hz); HPLC (254 nm, system A): t_R= 22.2 min; EI-MS: 252 $[M^{\cdot}]^{+}$.

Ethyl 2-(5-fluoro-[1,1'-biphenyl]-2-yl)-2-hydroxyacetate **(18)**

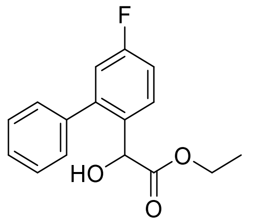

A solution of **16** (600 mg, 2.39 mmol) in dry THF (20 mL) was cooled to -78 °C. Subsequently, *n*-buthyllithium (2.5 M solution in hexane, 1.90 mL, 4.75 mmol) was added while the reaction temperature was maintained at a maximum of -75 °C. The mixture was stirred at -78 °C for 25 min, followed by the addition of glyoxylic acid ethyl ester solution (50% in toluene, 2.00 mL, 9.72 mmol, dissolved in dry THF (5 mL)), while the reaction temperature was maintained at a maximum of -70 °C. The reaction was allowed to warm to -25 °C over three hours, followed by the addition of cold saturated, aqueous NH_4Cl solution. The suspension was stirred at room temperature for additional 30 min and then extracted three times with chloroform. The combined organic layers were washed twice with water and once with saturated, aqueous NaCl solution and dried (Na_2SO_4). After evaporation, the crude residue was purified by column chromatography on silica gel (*n*-hexane/ethyl acetate, 10:1) to give **18** (124 mg, 19%) as a pale yellow oil. IR (NaCl): 3447, 2959, 1743, 1586, 1487, 1296, 1188, 1074, 1022, 764, 704 cm^{-1}; ^{1}H-NMR (600 MHz, $CDCl_3$): δ (ppm) = 7.47 – 7.39 (m, 5H), 7.37 (dd, *J* = 8.7, 5.7 Hz, 1H), 7.08 (td, *J* = 8.4, 2.7 Hz, 1H), 7.01 (dd, *J* = 9.4, 2.7 Hz, 1H), 5.17 (d, *J* = 4.7 Hz, 1H), 4.23 – 4.10 (m, 2H), 3.40 (d, *J* = 4.7 Hz, 1H), 1.19 (t, *J* = 7.1 Hz, 3H); ^{13}C-NMR (91 MHz, $CDCl_3$): δ (ppm) = 174.2, 162.3 (d, J = 249 Hz), 144.6 (d, J = 8.2 Hz), 139.4, 132.4 (d, J = 3.6 Hz), 129.6, 129.0 (d, J = 9.1 Hz), 128.4, 128.0, 117.2 (d, J = 21.8 Hz), 115.1 (d, J = 21.8 Hz), 69.4, 62.4, 14.1; HPLC (254 nm, system A): t_R= 20.7 min; ESI-MS: 297.3 $[M+Na]^{+}$.

(1R,3S,4R)-Quinuclidin-3-yl 2-(5-fluoro-[1,1'-biphenyl]-2-yl)-2-hydroxyacetate **(5a/5b)**

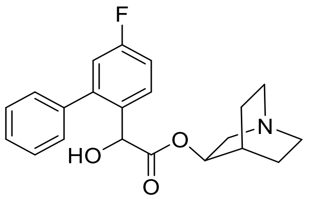

(1*R*,3*S*,4*R*)-Quinuclidin-3-yl 2-(5-fluoro-[1,1'-biphenyl]-2-yl)-2-hydroxyacetate **(5a/5b)** were prepared according to the protocol of compound **2**, using a solution of (*R)*-(-)-3-quinuclidinol (153 mg, 1.20 mmol) in dry DMF (4 mL), K_2CO_3 (179 mg, 1.30 mmol) and a solution of compound **18** (120 mg, 437 µmol) in dry DMF (1 mL). Purification by flash chromatography on silica gel (CH_2Cl_2/MeOH/NH_3 aqu. 25%, 10:1:0.01) gave a mixture of the diastereomers **5a**/**5b** (35.7 mg, 23%) as a a pale yellow oil. IR (NaCl): 3059, 2947, 1742, 1586, 1486, 1188, 1117, 1092, 1014, 756, 703 cm^{-1}; ^{1}H-NMR **5a** (600 MHz, $CDCl_3$): δ (ppm) = 7.48 – 7.40 (m, 5H), 7.38 (dd, *J* = 8.7, 5.7 Hz, 1H), 7.10 – 7.05 (m, 1H), 7.04 – 7.00 (m, 1H), 5.25 (s, 1H), 4.86 – 4.82 (m, 1H), 3.49 (s, 1H), 3.18 (ddd, *J* = 14.8, 8.4, 2.3 Hz, 1H), 2.81 – 2.66 (m, 4H), 2.66 – 2.61 (m, 1H), 1.91 – 1.86 (m, 1H), 1.66 – 1.60 (2H), 1.55 – 1.46 (1H), 1.24 – 1.15 (1H) (3xm); ^{1}H-NMR **5b** (600 MHz, $CDCl_3$): δ (ppm) = 7.47 – 7.38 (m, 6H), 7.12 – 7.08 (m, 1H), 7.03 (dd, *J* = 9.4, 2.7 Hz, 1H), 5.25 (s, 1H), 4.85 – 4.81 (m, 1H), 3.17 (ddd, *J* = 14.8, 8.2, 2.2 Hz, 1H), 2.83 – 2.69 (m, 3H), 2.59 – 2.53 (m, 1H), 2.48 – 2.43 (m, 1H), 2.00 – 1.96 (m, 1H), 1.71 – 1.65 (1H), 1.56 – 1.48 (2H), 1.38 – 1.28 (1H) (3xm); ^{13}C-NMR (600 MHz, $CDCl_3$): δ (ppm) = 173.6/173.4, 162.3 (d, J = 249 Hz)/162.2 (d, J = 251 Hz), 144.3 (d, J = 8.4 Hz)/144.2 (d, J = 8.4 Hz), 139.2/139.0, 131.9/131.7, 129.3/129.2, 128.8 (d, J = 8.4 Hz)/128.8 (d, J = 8.4 Hz), 128.6/128.5, 128.1/128.0, 117.3 (d, J = 11.2 Hz)/117.2 (d, J = 11.2 Hz), 115.3 (d, J = 22.4 Hz)/115.1 (d, J = 22.4 Hz), 70.6/70.4, 69.2/69.0, 53.6/53.4, 46.5/46.4, 45.6, 26.5/26.4, 22.2/22.0, 18.0/17.7; HPLC **5a**: (254 nm, system A): t_R= 16.1 min, purity: > 99%, (254 nm, system D): t_R= 13.9 min, purity: 95%; **5b**: (254 nm, system A): t_R= 15.8 min, purity: 97%, (254 nm, system D): t_R= 13.7 min, purity: 95%; HR-ESI-MS **5a**: calculated 356.1657, found 356.1657 $[M]^+$; HR-ESI-MS **5b**: calculated 356.1657, found 356.1659 $[M]^+$. The diastereomers were separated by preparative TLC (CH_2Cl_2/MeOH/NH_3 aqu. 25%, 20:1:0.02). The ratio of the diastereomers was determined via NMR. **5a:** *dr* = 92%; **5b:** *dr* = 98%

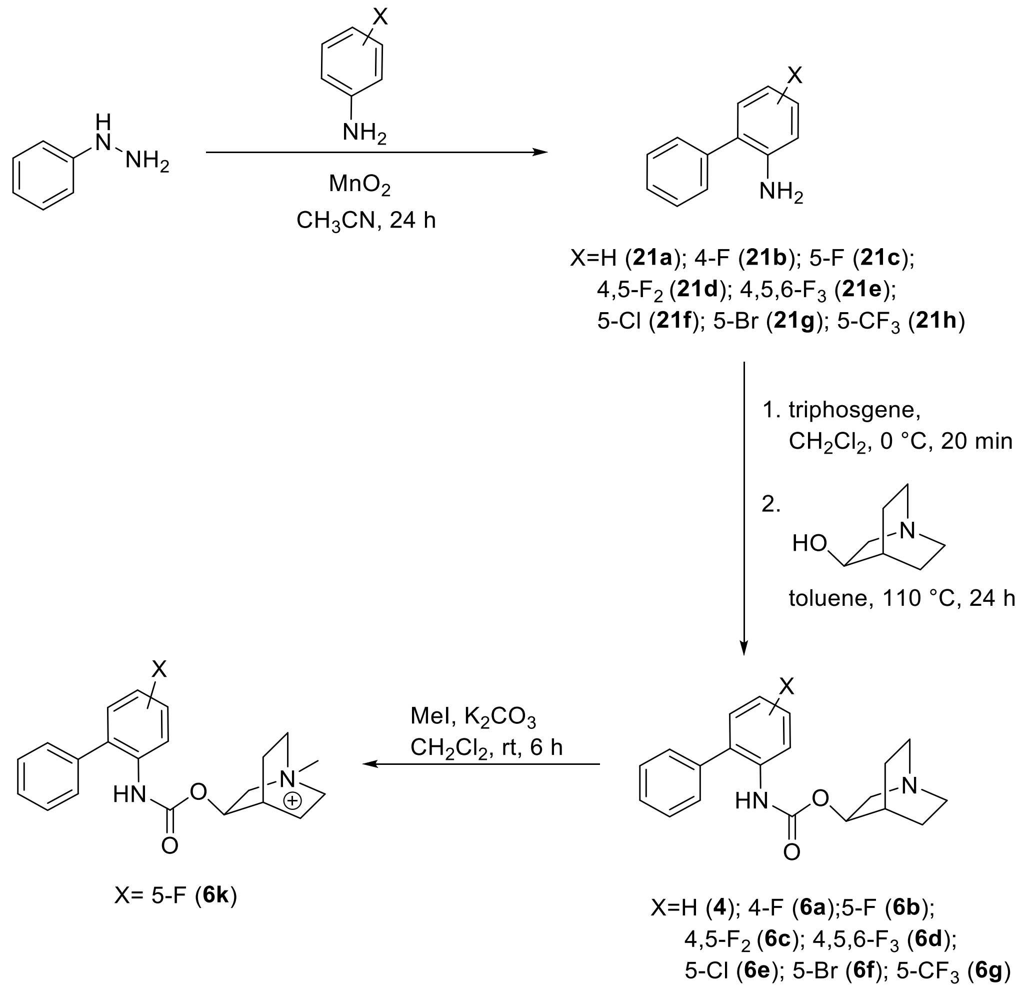

Biaryl synthesis from phenylhydrazine with MnO_2 (general procedure 1)

To a stirred suspension of the aniline derivative (20.0 mmol, 20 eq.) and MnO_2 (5 mmol, 5 eq.) in acetonitrile (5.0 mL), a solution of phenylhydrazine (108 mg, 0.098 mL, 1.00 mmol, 1.0 eq.) in acetonitrile (2.0 mL) was added over a course of 1 h. After termination of the reaction, the reaction mixture was filtered over celite and washed with ethyl acetate. Afterwards, the solvent was removed under reduced pressure and further purification of the biphenyl-2-amines was achieved using Kugelrohr distillation and subsequent flash column chromatography.

Synthesis of the biaryl carbamates using (R)-quinuclidin-3-ol (general procedure 2)

A solution of the biphenyl-2-amine (1.0 mmol, 1.0 eq.) in dry DCM (1.5 mL) under argon atmosphere was cooled to 0 °C. Over a course of 20 min, a solution of triphosgene (118 mg, 0.40 mmol, 0.40 eq.) in dry DCM (3.0 mL) was added slowly. The reaction mixture was stirred for another 15 min at 0 °C, the reaction progress was monitored using TLC. Afterwards, the solvent was removed under reduced pressure and the residue was redissolved in toluene (3.0 mL). A solution of the (*R*)-quinuclidin-3-ol (153 mg, 1.2 mmol, 1.2 eq.) in toluene (1.5 mL) was added and the reaction mixture was stirred at 110 °C for 24 h. After cooling to room temperature, the reaction mixture was diluted with ethyl acetate

(60 mL) and the organic phase was extracted with 1 n HCl (3 × 45 mL). The combined aqueous phases were adjusted to a pH of 9, using K_2CO_3 and subsequently extracted with chloroform (3 x 60 mL). The combined organic phases were then dried over Na_2SO_4. The crude product was purified by flash column chromatography.

Quaternization using methyl iodide (general procedure 3)

The respective quinuclidinyl carbamate (1 mmol, 1 eq.) was taken up in dry DCM (4.00 mL) at room temperature. Methyl iodide (710 mg, 0.31 mL, 5 mmol, 5.0 eq.) and K_2CO_3 (415 mg, 3 mmol, 3.0 eq.) were added and the reaction mixture was stirred for 6 h. Afterwards, the reaction mixture was filtered and the solvent was removed under reduced pressure. The quaternary ammonium compound was transformed into the trifluoroacetate salt using trifluoroacetic acid.

Biphenyl-2-amine **(21a)**

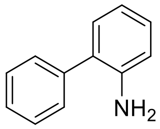

Biphenyl-2-amine (**21a**) was prepared analogously to the general procedure 1 from phenyl hydrazine (0.098 mL, 1.00 mmol), MnO_2 (453 mg, 5.00 mmol) and aniline (1.83 mL, 20.0 mmol). The excess of aniline was removed using Kugelrohr distillation under reduced pressure at 85 °C. The desired product **21a** was obtained by flash column chromatography (hexane / EtOAc = 8 : 1) as a brown oil (105 mg, 0.62 mmol, 62 %). R_f: 0.6 (hexane / EtOAc = 4 : 1) [UV]; ^{1}H-NMR (600 MHz, $CDCl_3$): δ (ppm) = 7.37 - 7.43 (m, 4 H), 7.28 - 7.32 (m, 1 H), 7.08 - 7.15 (m, 2 H), 6.82 (dt, *J* = 1.2 Hz, *J* = 7.5 Hz, 1 H), 6.70 (d, *J* = 8.0 Hz, 1 H).

4-Fluoro-[1,1'-biphenyl]-2-amine **(21b)**

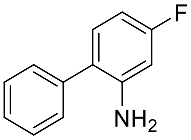

4-Fluoro-[1,1'-biphenyl]-2-amine (**21b**) was prepared analogously to the general procedure 1 from phenyl hydrazine (0.19 mL, 2.00 mmol), MnO_2 (870 mg, 10.0 mmol) and 3-fluoroaniline (4.44 g, 40.0 mmol). The excess of 3-fluoroaniline was removed using Kugelrohr distillation under reduced pressure at 90 °C. The desired product **21b** was obtained by flash column chromatography (hexane / EtOAc = 8 : 1) as a brown oil (47.0 mg, 250 µmol, 13 %). $\boldsymbol{R_f}$: 0.6 (hexane / EtOAc = 4 : 1) [UV]. ^{1}H-NMR (360 MHz, $CDCl_3$): δ (ppm) = 7.37 - 7.45 (m, 4 H), 7.34 (m, 1 H), 7.04 (dd, *J* = 6.5 Hz, *J* = 8.4 Hz, 1 H), 6.50 (dt, *J* = 2.5 Hz, *J* = 8.4 Hz, *J* = 8.4 Hz, 1 H), 6.45 (dd, *J* = 2.5 Hz, *J* = 10.7 Hz, 1 H), 3.72 (bs, 2 H).

5-Fluoro-[1,1'-biphenyl]-2-amine **(21c)**

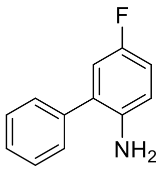

5-Fluoro-[1,1'-biphenyl]-2-amine (**21c**) was prepared analogously to the general procedure 1 from phenyl hydrazine (0.10 mL, 1.00 mmol), MnO_2 (453 mg, 5.00 mmol) and 4-fluoroaniline (1.92 g, 20.0 mmol). The excess of 4-fluoroaniline was removed using Kugelrohr distillation under reduced pressure at 85 °C. The desired product **21c** was obtained by flash column chromatography (hexane / EtOAc = 10 : 1) as a brown oil (138 mg, 559 µmol, 56 %). R_f: 0.6 (Hexan / EtOAc = 4 : 1) [UV], ^{1}H-NMR (600 MHz, $CDCl_3$): δ (ppm) = 7.42 - 7.47 (m, 5 H), 7.34 - 7.47 (m, 1 H), 6.86 - 6.90 (m, 1 H), 6.73 - 6.75 (m, 1 H), 3.54 (bs, 2 H); ^{13}C-NMR (91 MHz, $CDCl_3$): δ (ppm) = 156.4 (d, J = 236.9 Hz, C_q), 139.4 (d, J = 2.2 Hz, C_q), 133.6 (C_q), 129.0 (CH), 128.9 (2 × CH), 128.8 (d, J = 7.2 Hz, C_q), 127.6 (2 × CH), 116.6 (d, J = 26.7 Hz, CH), 116.5 (d, J = 3.6 Hz, CH), 114.8 (d, J = 22.3 Hz, CH); ^{19}F-NMR (235 MHz, $CDCl_3$): δ (ppm) = −129.3.

4,5-Difluoro-[1,1'-biphenyl]-2-amine **(21d)**

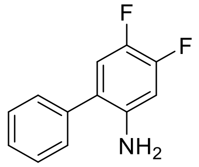

4,5-Difluoro-[1,1'-biphenyl]-2-amine (**21d**) was prepared analogously to the general procedure 1 from phenyl hydrazine (0.10 mL, 1.00 mmol), MnO_2 (453 mg, 5.00 mmol) and 3,4-difluoroaniline (1.98 ml, 20.0 mmol). The excess of 3,4-difluoroaniline was removed using Kugelrohr distillation under reduced pressure at 85 °C. The desired product **21d** was obtained by flash column chromatography (hexane / EtOAc = 8 : 1) as dark-red crystals (38.0 mg, 184 µmol, 18 %). $\boldsymbol{R_f}$: 0.6 (hexane / EtOAc = 4 : 1) [UV]; ^{1}H-NMR (360 MHz, $CDCl_3$): δ (ppm) = 7.32 - 7.46 (m, 5 H), 6.92 (dd, J = 8.7 Hz, J_{HF} = 11.0 Hz, 1 H), 6.53 (dd, J = 7.0 Hz, J_{HF} = 12.0 Hz, 1 H), 3.66 (bs, 2 H).

4,5,6-Trifluoro-[1,1'-biphenyl]-2-amine **(21e)**

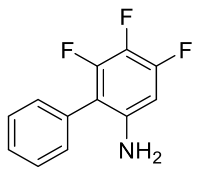

4,5,6-Trifluoro-[1,1'-biphenyl]-2-amine (**21e**) was prepared analogously to the general procedure 1 from phenyl hydrazine (0.10 mL, 1.00 mmol), MnO_2 (453 mg, 5.00

mmol) and 3,4,5-trifluoroaniline (735 mg, 5.00 mmol). The excess of 3,4,5-trifluoroaniline was removed using Kugelrohr distillation under reduced pressure at 100 °C. The desired product **21e** was obtained by flash column chromatography (hexane / EtOAc = 10 : 1) as brown crystals (40.0 mg, 179 µmol, 18 %). R_f: 0.4 (hexane / EtOAc = 4 : 1) [UV]; ^{1}H-NMR (360 MHz, $CDCl_3$): δ (ppm) = 7.49 (m, 2 H), 7.42 (m, 1 H), 7.35 (d, *J* = 6.0 Hz, 2 H), 6.34 (ddd, *J* = 2.2 Hz, *J* = 6.3 Hz, *J* = 11.8 Hz, 1 H), 3.60 (bs, 2 H); EI-MS: *m/z* (%): 344 (13), 341 (11), 332 (13), 330 (19), 324 (14), 319 (64), 294 (12), 254 (14), 252 (16), 244 (11), 241 (17), 235 (13), 232 (18), 225 (17), 223 (100), 222 (47), 221 (31), 217 (22), 212 (30), 202 (15), 201 (12), 182 (26), 174 (15), 169 (22), 163 (28), 162 (35); HR-EI-MS: calculated 223.0609, found 223.0609 $[M+H]^+$.

5-Chloro-[1,1'-biphenyl]-2-amine **(21f)**

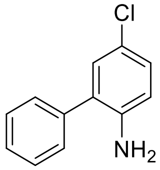

5-Chloro-[1,1'-biphenyl]-2-amine (**21f**) was prepared analogously to the general procedure 1 from phenyl hydrazine (0.10 mL, 1.00 mmol), MnO_2 (453 mg, 5.00 mmol) and 4-chloroaniline (2.54 g, 20.0 mmol). The excess of 4-chloroaniline was removed using Kugelrohr distillation under reduced pressure at 90 °C. The desired product **21f** was obtained by flash column chromatography (hexane / EtOAc = 10 : 1) as brown crystals (89.0 mg, 440 µmol, 44 %). R_f: 0.4 (hexane / EtOAc = 4 : 1) [UV]; ^{1}H-NMR (600 MHz, $CDCl_3$): δ (ppm) = 7.40 - 7.46 (m, 4 H), 7.34 - 7.37 (m, 1 H), 7.09 - 7.13 (m, 2 H), 6.73 (d, *J* = 9.0 Hz, 1 H); ^{13}C-NMR (91 MHz, $CDCl_3$): δ (ppm) = 141.2 (C_q), 138.1 (C_q), 130.0 (C_q), 129.5 (C_q), 129.0 (2 × CH), 128.9 (2 × CH), 127.7 (CH), 128.2 (CH), 117.4 (CH), 123.8 (CH); EI-MS *m/z* (%): 206 (4), 205 (26), 204 (20), 203 (100), 202 (27), 168 (10), 167 (44), 166 (7), 140 (3), 139 (7), 101 (3); HR-EI-MS: calculated 203.0501, found 203.0502.

5-Bromo-[1,1'-biphenyl]-2-amine **(21g)**

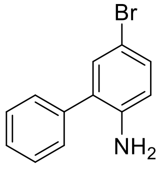

5-Bromo-[1,1'-biphenyl]-2-amine (**21g**) was prepared analogously to the general procedure 1 from phenyl hydrazine (0.10 mL, 1.00 mmol), MnO_2 (453 mg, 5.00 mmol) and 4-bromoaniline (3.44 g, 20.0 mmol). The excess of 4-bromoaniline was removed using Kugelrohr distillation under reduced pressure at 105 °C. The desired product **21g** was obtained by flash column chromatography (hexane / EtOAc = 8 : 1) as brown crystals (138 mg, 559 µmol, 56 %). R_f: 0.4 (hexane / EtOAc = 4 : 1) [UV]; ^{1}H-NMR (600 MHz, $CDCl_3$): δ (ppm) = 7.40 - 7.46 (m, 4 H), 7.34 - 7.37 (m, 1 H), 7.26 (d, *J* = 8.0 Hz, 2 H), 6.63 - 7.66 (m, 1 H); ^{13}C-NMR (91 MHz, $CDCl_3$): δ (ppm) = 141.6 (C_q), 137.9 (C_q), 132.8

(CH), 131.1 (C_q), 130.0 (C_q), 129.0 (2 × CH), 128.9 (2 × CH), 127.8 (CH), 117.6 (CH), 110.0 (CH); EI-MS *m/z* (%): 250 (11), 249 (84), 248 (27), 247 (100), 246 (17), 182 (7), 169 (10), 168 (17), 167 (75), 166 (16), 142 (8), 141 (17), 115 (8), 98 (6); HR-EI-MS: calculated 246.9996, found 246.9997 [M].

5-(Trifluoromethyl)-[1,1'-biphenyl]-2-amine **(21h)**

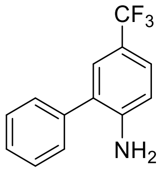

5-(Trifluoromethyl)-[1,1'-biphenyl]-2-amine (**21h**) was prepared analogously to the general procedure 1 from phenyl hydrazine (0.10 mL, 1.00 mmol), MnO_2 (453 mg, 5.00 mmol) and 4-(trifluoromethyl)aniline (0.63 ml, 5.00 mmol). The excess of 4-(trifluoromethyl)aniline was removed using Kugelrohr distillation under reduced pressure at 90 °C. The desired product **21h** was obtained by flash column chromatography (hexane / EtOAc = 8 : 1) as red crystals (44.0 mg, 186 µmol, 19 %). R_f: 0.5 (hexane / EtOAc = 4 : 1) [UV]; ^{1}H-NMR (600 MHz, $CDCl_3$): δ (ppm) = 7.32 - 7.50 (m, 7 H), 6.77 (d, *J* = 8.1 Hz, 1 H), 4.00 (bs, 2 H); ^{13}C-NMR (91 MHz, $CDCl_3$): δ (ppm) = 138.1 (C_q), 129.1 (C_q), 128.9 (C_q), 127.8 (2 × CH), 127.6 (q, *J* = 3.8 Hz, CH), 127.0 (CH), 125.6 (q, *J* = 3.8 Hz, CH), 124.8 (q, *J* = 270.0 Hz, C_q), 120.4 (q, *J* = 32.7 Hz, C_q), 114.8 (2 × CH), 112.8 (CH); EI-MS *m/z* (%): 238 (16), 237 (100), 236 (52), 235 (7), 218 (7), 216 (10), 168 (12), 167 (37), 166 (5), 139 (4), 108 (4), 83 (5), 28 (7); HR-EI-MS: calculated 237.0766, found 237.0765 $[M]^+$.

(1S,3R,4S)-Quinuclidin-3-yl-biphen-2-ylcarbamate **(4)** *(24)*

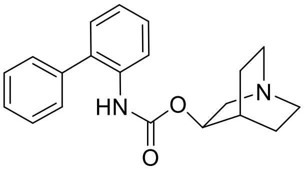

(1*S*,3*R*,4*S*)-Quinuclidin-3-yl-biphen-2-ylcarbamate (**4**) was prepared analogously to the general procedure 2 from triphosgene (35.6 mg, 120 µmol), biphenyl-2-amine (50.0 mg, 300 µmol) and (*R*)-quinuclidin-3-ol (45.7 mg, 360 µmol). The carbamate **4** was obtained by flash column chromatography (DCM / methanol = 15 : 1) as a beige oil (29 mg, 90.0 µmol, 30 %). $\boldsymbol{R_f}$: 0.3 (CH_2Cl_2 / EtOAc = 15 : 1) [UV]; ^{1}H-NMR (600 MHz, $CDCl_3$): δ (ppm) = 8.05 (bs, 1 H), 7.49 (t, *J* = 7.4 Hz, 2 H), 7.42 (t, *J* =7.4 Hz, 1 H), 7.34 – 7.39 (m, 3 H), 7.23 (dd, *J* = 1.6 Hz, *J* = 7.6 Hz, 1 H), 7.15 (dt, *J* = 1.2 Hz, *J* = 7.5 Hz, 1 H), 6.68 (s, 1 H), 4.79 – 4.83 (m, 1 H), 3.27 (dd, *J* = 8.5 Hz, *J* = 14.6 Hz, 1 H), 2.72 – 2.92 (m, 5 H), 2.07 – 2.11 (m, 1 H), 1.69 – 1.81 (m, 2 H), 1.57 – 1.64 (m, 1 H), 1.38 – 1.44 (m, 1 H); ^{13}C-NMR (91 MHz, $CDCl_3$): δ (ppm) = 153.2 (C_q).138.1 (C_q), 134.6 (C_q), 130.2 (CH), 129.2 (CH), 129.1 (2 x CH), 129.1 (CH), 128.4 (C_q), 127.9 (2 x CH), 125.6 (CH), 123.7 (CH), 71.5, 55.0, 47.1, 46.3, 25.2, 24.0, 19.1; HPLC (254 nm, system A): t_R = 15.2 min, purity: 98%, (254 nm, system D): t_R = 14.3 min, purity: 97%; EI-MS: *m/z* (%): 322 (51)

[M$^+$], 195 (30), 178 (12), 167 (27), 127 (20), 126 (100), 110 (58), 109 (55), 98 (11), 82 (24), 55 (12), 42 (28); HR-EI-MS: calculated 322.1681, found 322.1682 [M+H]$^+$.

(1S,3R,4S)-Quinuclidin-3-yl (4-fluoro-[1,1'-biphenyl]-2-yl)carbamate **(6a)** *(26)*

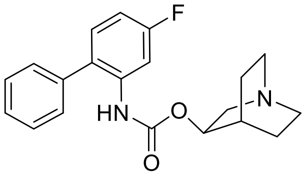

(1*S*,3*R*,4*S*)-Quinuclidin-3-yl (4-fluoro-[1,1'-biphenyl]-2-yl)carbamate (**6a**) was prepared analogously to the general procedure 2 from triphosgene (59.0 mg, 200 µmol), 4-fluoro-[1,1'-biphenyl]-2-amine (93.0 mg, 500 µmol) and (*R*)-quinuclidin-3-ol (76.0 mg, 144 µmol). The carbamate **6a** was obtained by flash column chromatography (DCM / methanol = 15 : 1) as a beige oil (38.0 mg, 112.0 µmol, 22 %). *R*$_f$: 0.3 (CH_2Cl_2 / EtOAc = 15 : 1) [UV], ^{1}H-NMR (600 MHz, $CDCl_3$): δ (ppm) = 7.95 (bs, 1 H), 7.50 (t, *J* = 7.4 Hz, 1 H), 7.45 (t, *J* = 7.5 Hz, 1 H), 7.33 – 7.36 (m, 2 H), 7.16 (dd, *J* = 6.4 Hz, *J* = 8.5 Hz, 1 H), 6.81 – 6.85 (m, 1 H), 6.75 (s, 1 H), 4.78 – 4.83 (m, 1 H), 3.26 (dd, *J* = 8.5 Hz, *J* = 14.6 Hz, 1 H), 2.73 – 2.87 (m, 5 H), 2.04 – 2.10 (m, 1 H), 1.71 – 1.74 (m, 2 H), 1.55 – 1.65 (m, 1 H), 1.39 - 1.40 (m, 1 H); ^{13}C-NMR (91 MHz, $CDCl_3$): δ (ppm) = 162.5 (d, *J* = 248.2 Hz, C_q), 152.9 (C_q), 137.2 (CH), 136.2 (C_q), 136.1 (C_q), 131.2 (d, *J* = 9.4 Hz, CH), 129.3 (CH), 129.3 (CH), 129.2 (2 x CH), 128.1 (CH), 110.0 (d, *J* = 21.6 Hz, CH), 72.2, 55.1, 47.2, 46.3, 25.2, 24.3, 19.2; HPLC (254 nm, system A): t_R = 16.4 min, purity: 99%, (254 nm, system D): t_R = 14.5 min, purity: 96%; EI-MS *m/z* (%): 322 (51) [M$^+$], 195 (30), 178 (12), 167 (27), 127 (20), 126 (100), 110 (58), 109 (55), 98 (11), 82 (24), 55 (12), 42 (28); HR-EI-MS calculated 341.1659, found: 341.1660 [M+H]$^+$.

(1S,3R,4S)-Quinuclidin-3-yl (5-fluoro-[1,1'-biphenyl]-2-yl)carbamate **(6b)** *(26)*

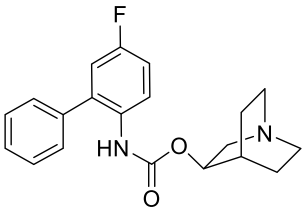

(1*S*,3*R*,4*S*)-Quinuclidin-3-yl (5-fluoro-[1,1'-biphenyl]-2-yl)carbamate (**6b**) was prepared analogously to the general procedure 2 from triphosgene (120 mg, 400 µmol), 5-fluoro-[1,1'-biphenyl]-2-amine (189 mg, 1.00 mmol) and (*R*)-quinuclidin-3-ol (155 mg, 1.20 mmol). The carbamate **6b** was obtained by flash column chromatography (DCM / methanol = 15 : 1) as a beige oil (206 mg, 600 µmol, 60 %). ***R*$_f$**: 0.3 (CH_2Cl_2 / EtOAc = 15 : 1) [UV]. ^{1}H-NMR (600 MHz, $CDCl_3$, trifluoracetate salt): δ (ppm) = 7.93 (bs, 1 H), 7.44 – 7.52 (m, 3 H), 7.34 (d, *J* = 7.0 Hz, 2 H), 7.08 (dd, *J* = 2.9 Hz, *J* = 8.9 Hz, 1 H), 6.98 – 7.05 (m, 1 H), 6.58 (bs, 1 H), 5.06 (m, 1 H), 3.23 (dd, *J* = 8.4 Hz, *J* = 14.6 Hz, 1 H), 2.66 – 2.88 (m, 5 H), 2.01 – 2.05 (m, 1 H), 1.64 – 1.74 (m, 2 H), 1.52 – 1.61 (m, 1 H), 1.32 – 1.40 (m, 1 H); ^{13}C-NMR (151 MHz, $CDCl_3$): δ (ppm) = 159.1 (d, *J* = 244.1 Hz, C_q), 153.3 (C_q), 137.1 (d, *J* = 2.8 Hz, C_q), 130.4

(d, J = 2.8 Hz, C_q), 129.1 (2 x CH), 128.9 (2 x CH), 128.3 (CH), 116.7 (d, J = 23.0 Hz, CH), 114.7 (d, J = 22.1 Hz, CH), 70.8, 54.5, 46.8, 45.9, 24.9, 23.2, 18.6; HPLC (254 nm, system A): t_R = 16.8 min, purity: 98%, (254 nm, system D): t_R = 14.4 min, purity: 96%; HR-EI-MS: calculated 341.1660, found: 341.1656 $[M+H]^+$.

(1S,3R,4S)-Quinuclidin-3-yl (4,5-difluoro-[1,1'-biphenyl]-2-yl)carbamate **(6c)**

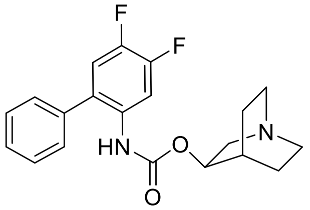

(1*S*,3*R*,4*S*)-Quinuclidin-3-yl (4,5-difluoro-[1,1'-biphenyl]-2-yl)carbamate (**6c**) was prepared analogously to the general procedure 2 from triphosgene (22.0 mg, 74.0 µmol), 4,5-difluoro-[1,1'-biphenyl]-2-amine (38.0 mg, 180 µmol) and (*R*)-quinuclidin-3-ol (28.0 mg, 89.0 µmol). The carbamate **6c** was obtained by flash column chromatography (DCM / methanol = 15 : 1) as a beige oil (206 mg, 600 µmol, 60 %). R_f: 0.3 (CH_2Cl_2 / EtOAc = 15 : 1) [UV]; ^{1}H-NMR (600 MHz, $CDCl_3$): δ (ppm) = 8.01 (bs, 1 H), 7.47 – 7.54 (m, 2 H), 7.42 – 7.48 (m, 1H), 7.30 – 7.35 (m, 2 H), 7.03 (dd, J = 8.5 Hz, J = 10.5 Hz, 1 H), 6.59 (s, 1 H), 4.82 – 4.84 (m, 1 H), 3.25 – 3.35 (m, 1 H), 2.75 – 2.91 (m, 5 H), 2.08 (dd, J = 3.09 Hz, 6.25 Hz, 1 H), 1.74 – 1.80 (m, 2 H), 1.60 – 1.65 (m, 1 H), 1.43 – 1.48 (m, 1 H); ^{13}C-NMR (91 MHz, $CDCl_3$): δ (ppm) = 150.9 (d, J = 12.9 Hz, CH), 135.9 (C_q), 129.5 (2 x CH), 129.4 (2 x CH), 129.0 (CH), 128.8 (CH), 118.7 (d, J = 18.4 Hz, CH), 118.6 (C_q), 67.9, 53.0, 46.2, 45.4, 24.3, 20.6, 17.0; HPLC (254 nm, system A): t_R = 16.7 min, purity: 98%, (254 nm, system D): t_R = 14.3 min, purity: 99%; HR-EI-MS: calculated 359.1563, found 359.1565 $[M+H]^+$.

(1S,3R,4S)-Quinuclidin-3-yl (4,5,6-trifluoro-[1,1'-biphenyl]-2-yl)carbamate **(6d)**

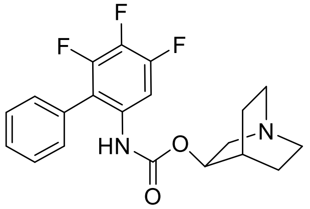

(1*S*,3*R*,4*S*)-Quinuclidin-3-yl (4,5,6-trifluoro-[1,1'-biphenyl]-2-yl)carbamate (**6d**) was prepared analogously to the general procedure 2 from triphosgene (20.5 mg, 69.0 µmol), 4,5,6-trifluoro-[1,1'-biphenyl]-2-amine (38.0 mg, 173 µmol) and (*R*)-quinuclidin-3-ol (26.3 mg, 207 µmol). The carbamate **6d** was obtained by flash column chromatography (DCM / methanol = 10 : 1) as a beige oil (30 mg, 80 µmol, 46 %). R_f: 0.3 (CH_2Cl_2 / EtOAc = 15 : 1) [UV]; ^{1}H-NMR (600 MHz, $CDCl_3$): δ (ppm) = 7.92 (bs, 1 H), 7.53 – 7.58 (m, 2 H), 7.48 – 7.53 (m, 1 H), 7.32 (d, J = 8.3 Hz, 2 H), 6.47 (s, 1 H), 4.76 (dt, J = 2.8 Hz, 7.0 Hz 1 H), 3.23 (dd, J = 8.5 Hz, J = 14.6 Hz, 1 H), 2.63 – 2.89 (m, 5 H), 2.00 – 2.04 (m, 1 H), 1.62 – 1.74 (m, 2 H), 1.52 - 1.60 (m, 1 H), 1.32 – 1.40 (m, 1 H); ^{13}C-NMR (151 MHz, $CDCl_3$): δ (ppm) = 151.7 (C_q), 150.3 (ddd, J = 5.2 Hz, 10.0 Hz, 247.2 Hz, C_q),

148.6 (ddd, J = 5.0 Hz, 10.5 Hz, 246.0 Hz, C_q), 136.5 (ddd, J = 15.8 Hz, 15.8 Hz, 248.1 Hz, C_q), 130.1 (2 x CH), 129.6 (CH), 129.6 (2 x CH), 128.7 (C_q), 103.5 – 103.9 (m), 68.0, 53.0, 46.2, 45.4, 24.1, 20.5, 16.8; HPLC (254 nm, system A): t_R = 17.1 min, purity: 98%, (254 nm, system D): t_R = 14.9 min, purity: 98%; HR-EI-MS: calculated 377.1475, found 377.1472 $[M+H]^+$.

(1S,3R,4S)-Quinuclidin-3-yl (5-chloro-[1,1'-biphenyl]-2-yl)carbamate **(6e)**

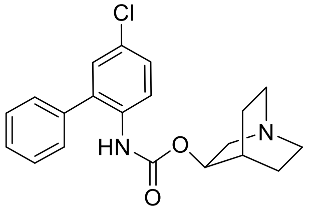

(1*S*,3*R*,4*S*)-Quinuclidin-3-yl (5-chloro-[1,1'-biphenyl]-2-yl)carbamate (**6e**) was prepared analogously to the general procedure 2 from triphosgene (52.2 mg, 176 µmol), 5-chloro-[1,1'-biphenyl]-2-amine (89.0 mg, 440 µmol) and (*R*)-quinuclidin-3-ol (673 mg, 528 µmol). The carbamate **6e** was obtained by flash column chromatography (DCM / methanol = 12 : 1) as a beige oil (126 mg, 353 µmol, 80 %). R_f: 0.3 (CH_2Cl_2 / EtOAc = 15 : 1) [UV]; ^{1}H-NMR (600 MHz, $CDCl_3$): δ (ppm) = 7.49 (t, J = 7.4 Hz, 2 H), 7.41 – 7.47 (m, 1 H), 7.33 – 7.36 (m, 2 H), 7.31 (dd, J = 2.6 Hz, J = 8.8 Hz, 1 H), 7.21 (d, J = 2.5 Hz, 1 H), 6.58 (s, 1 H), 4.74 – 4.80 (m, 1 H), 3.23 (dd, J = 8.4 Hz, J = 14.6 Hz, 1 H), 2.66 – 2.88 (m, 5 H), 2.01 – 2.05 (m, 1 H), 1.64 – 1.74 (m, 2 H), 1.52 – 1.61 (m, 1 H), 1.32 – 1.40 (m, 1 H); ^{13}C-NMR (151 MHz, $CDCl_3$): δ (ppm) = 136.8 (C_q), 133.4 (C_q), 129.9 (CH), 129.2 (2 x CH), 129.0 (2 x CH), 128.6 (CH), 128.3 (CH), 71.3, 55.2, 47.3, 46.4, 25.3, 24.4, 19.3; HPLC (254 nm, system A): t_R = 17.0 min, purity: 99%, (254 nm, system D): t_R = 14.8 min, purity: 98%; EI-MS *m/z* (%): 419 (17), 358 (32), 357 (31), 356 (100), 295 (43), 267 (38), 260 (14), 256 (13), 231 (36), 230 (13), 229 (100), 203 (16), 201 (26), 167 (12), 166 (43), 140 (13), 139 (11), 126 (100), 110 (79), 109 (69), 100 (13), 85 (26), 83 (19), 82 (38), 81 (19), 82 (38), 81 (18), 69 (14), 55 (12), 42 (27), 41 (21), 28 (45); HR-EI-MS: calculated 356.1292, found 356.1292 $[M+H]^+$.

(1S,3R,4S)-Quinuclidin-3-yl (5-bromo-[1,1'-biphenyl]-2-yl)carbamate **(6f)**

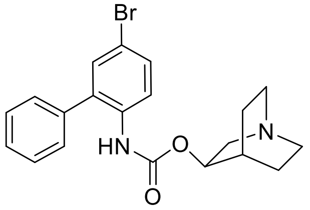

(1*S*,3*R*,4*S*)-Quinuclidin-3-yl (5-bromo-[1,1'-biphenyl]-2-yl)carbamate (**6f**) was prepared analogously to the general procedure 2 from triphosgene (52.2 mg, 176 µmol), 5-bromo-[1,1'-biphenyl]-2-amine (111 mg, 440 µmol) and (*R*)-quinuclidin-3-ol (673 mg, 528 µmol). The carbamate **6f** was obtained by flash column chromatography (DCM / methanol = 12 : 1) as a beige oil (69.0 mg, 172 µmol, 39 %). R_f 0.1 (CH_2Cl_2 / EtOAc = 10 : 1) [UV]; ^{1}H-NMR (600 MHz, $CDCl_3$): δ (ppm) = 7.97 (bs, 1 H), 7.32 – 7.53 (m, 4 H), 7.3 – 7.38

(m, 3 H), 6.58 (s, 1 H), 4.83 – 4.88 (m, 1 H), 3.34 (dd, J = 8.5 Hz, J = 14.6 Hz, 1 H), 2.82 – 2.98 (m, 5 H), 2.15 – 2.20 (m, 1 H), 1.76 – 1.86 (m, 2 H), 1.63 – 1.71 (m, 1H), 1.45 – 1.53 (m, 1 H); ^{13}C-NMR (91 MHz, $CDCl_3$): δ (ppm) = 136.6 (C_q), 133.6 (C_q), 132.9 (CH), 131.3 (CH), 129.3 (2 x CH), 129.1 (2 x CH), 128.6 (CH), 120.1 (C_q), 116.5 (CH), 77.2, 54.6, 47.0, 46.1, 25.0, 23.2, 18.6; HPLC (254 nm, system A): t_R = 17.8 min, purity: 97%, (254 nm, system D): t_R = 15.5 min, purity: 97%; EI-MS *m/z* (%): 402 (12), 400 (15), 276 (14), 275 (99), 274 (20), 273 (100), 247 (20), 167 (26), 166 (87), 164 (14), 140 (16), 139 (28), 127 (15), 126 (42), 110 (36), 109 (30), 83 (23), 82 (17), 82 (23), 69 (23), 69 (15), 63 (15), 51 (11), 44 (12), 43 (12), 42 (40), 36 (34), 32 (12), 28 (22); HR-EI-MS: calculated 400.0786, found 400.0786 $[M]^+$.

(1S,3R,4S)-Quinuclidin-3-yl (5-{trifluoromethyl}-[1,1'-biphenyl]-2-yl)carbamate **(6g)**

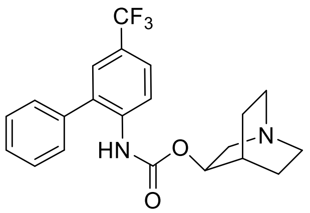

(1*S*,3*R*,4*S*)-Quinuclidin-3-yl (5-{trifluoromethyl}-[1,1'-biphenyl]-2-yl)carbamate (**6g**) was prepared analogously to the general procedure 2 from triphosgene (17.8 mg, 60.0 µmol), 5-(trifluoromethyl)-[1,1'-biphenyl]-2-amine (40.0 mg, 150 µmol) and (*R*)-quinuclidin-3-ol (22.9 mg, 180 µmol).The carbamate **6g** was obtained by flash column chromatography (DCM / methanol = 10 : 1) as a beige oil (12.0 mg, 31.0 µmol, 20 %). R_f: 0.2 (CH_2Cl_2 / EtOAc = 12 : 1) [UV]; ^{1}H-NMR (360 MHz, $CDCl_3$): δ (ppm) = 8.29 (d, J = 8.7 Hz, 2 H), 7.62 (dd, J = 2.0 Hz, J = 8.7 Hz, 1 H), 7.45 – 7.57 (m, 4 H), 7.38 (d, J = 6.7 Hz, 2 H), 6.81 (s, 1 H), 4.82 – 4.90 (m, 1 H), 3.26 – 3.38 (m, 1 H), 2.76 – 2.98 (m, 5 H), 2.10 – 2.16 (m, 1 H), 1.71 – 1.86 (m, 2H), 1.58 – 1.70 (m, 1 H), 1.39 – 1.52 (m, 1 H); ^{13}C-NMR (91 MHz, $CDCl_3$): δ (ppm) = 152.7 (C_q), 137.7 (C_q), 136.5 (C_q), 131.2 (C_q), 129.4 (2 x CH), 129.1 (2 x CH), 128.7 (CH), 127.2 (q, J = 3.7 Hz, CH), 125.5 (q, J = 3.9 Hz, CH), 123.8 (q, J = 232.4 Hz, C_q), 119.1 (CH), 71.2, 54.7, 47.0, 46.2, 30.8, 25.1, 23.8, 18.9; HPLC (254 nm, system A): t_R = 18.2 min, purity: 99%, (254 nm, system D): t_R = 15.6 min, purity: 96%; EI-MS *m/z* (%): 391 (11), 390 (45), 390 (65), 378 (26), 368 (14), 344 (19), 282 (13), 279 (12), 263 (20), 263 (32), 262 (100), 235 (18), 127 (21), 126 (100), 110 (26), 109 (49), 98 (12), 82 (30), 69 (10), 57 (10), 42 (22)41 (13); HR-EI-MS: calculated 390.1555, found 390.1555 $[M]^+$.

(1S,3R,4S)-3-[({5-Fluoro-(1,1'-biphenyl)-2-yl}carbamoyl)oxy]-1-methylquinuclidin-1-ium trifluoroacetate **(6k)**

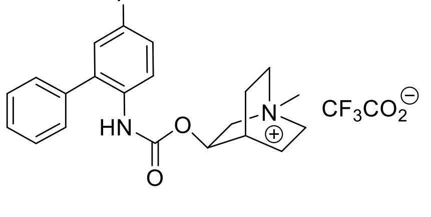

(1*S*,3*R*,4*S*)-3-[({5-Fluoro-(1,1'-biphenyl)-2-yl}carbamoyl)oxy]-1-methylquinuclidin-1-ium trifluoroacetate (**6k**) was prepared analogously to the general procedure 3 from **6b** (182 mg, 536 µmol), methyl iodide (168 µL, 2.68 mmol) and K_2CO_3 (222 mg, 1.61 mmol). The quaternary ammonium trifluoroacetate **6k** was purified by preparative HPLC(column 1, eluent: CH_3CN / H_2O + 0.1% HCO_2H (0 - 3 min, 5 - 5 % CH_3CN, 3 - 15 min, 5 – 95 % CH_3CN, 15 – 18 min, 95 – 95 % CH_3CN)) and was obtained as a beige oil. ^{1}H-NMR (600 MHz, $CDCl_3$): δ (ppm) = 7.50 (bs, 1 H), 7.45 – 7.48 (m, 2 H), 7.34 – 7.43 (m, 3 H), 7.05 – 7.12 (m, 2 H), 4.88 – 5.00 (m, 1 H), 3.71 – 3.82 (m, 1 H), 3.41 – 3.51 (m, 2 H), 3.30 – 3.36 (m, 1 H), 3.12 – 3.25 (m, 1 H), 2.95 (s, 3 H), 2.30 (m, 1 H), 2.12 – 2.21 (m, 1H), 2.02 – 2.10 (m, 1 H), 1.83 – 1.98 (m, 2 H); ^{13}C-NMR (151 MHz, $CDCl_3$): δ (ppm) = 139.7 (C_q), 131.6 (d, J_{CF} = 2.9 Hz, C_q), 130.0 (2 × CH), 129.7 (2 x CH), 129.0 (d, CH), 117.9 (d, J_{CF} = 22.8 Hz, CH), 115.5 (d, J_{CF} = 22.0 Hz, CH), 69.3, 64.2, 58.0, 57.1, 52.3, 25.2, 22.1, 19.3; HPLC (254 nm, system A): t_R = 16.2 min, purity: 98%, (254 nm, system D): t_R = 14.8 min, purity: 97%; HR-EI-MS: calculated 355.1809, found 355.1805 $[M]^+$.

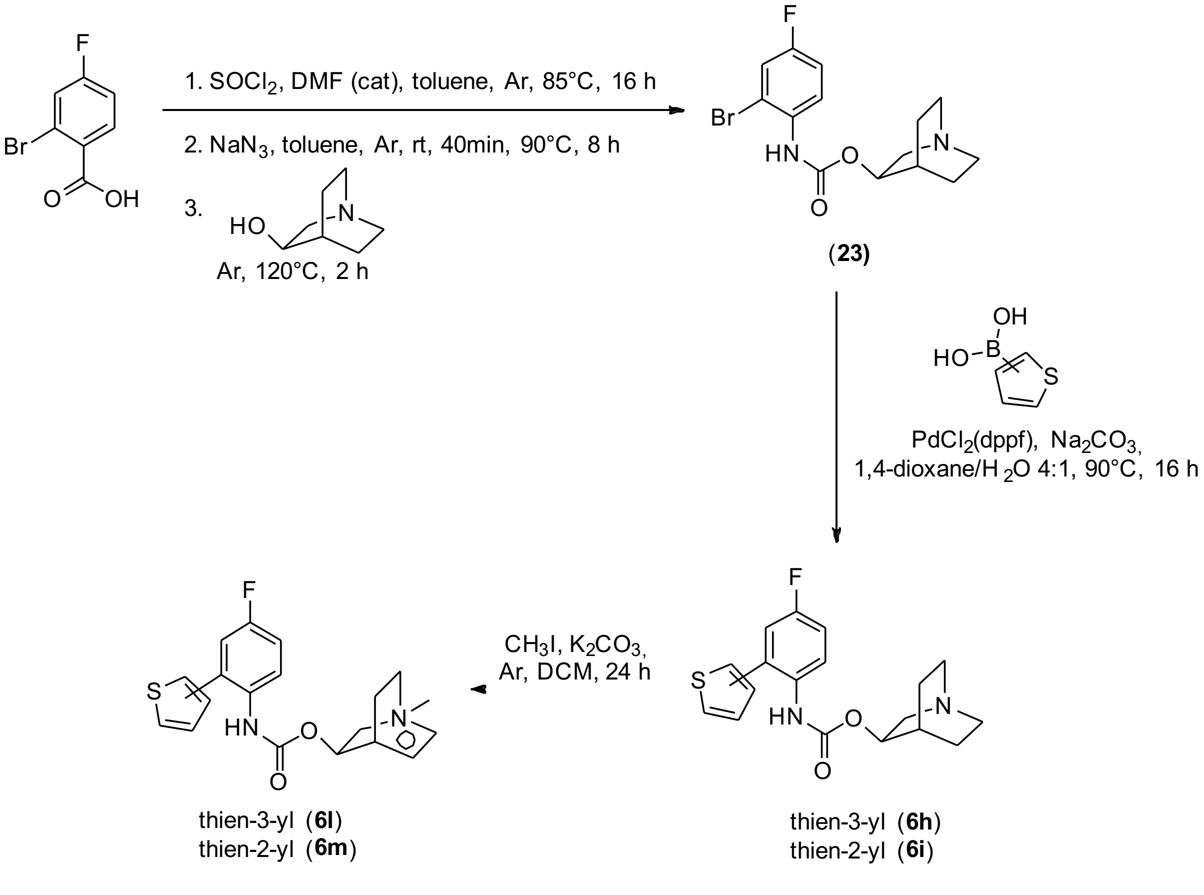

(1S,3R,4S)-Quinuclidin-3-yl (2-bromo-4-fluorophenyl)carbamate **(23)**

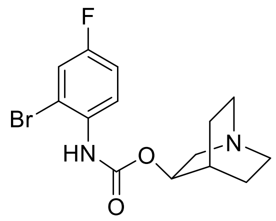

To a solution of 2-bromo-4-fluorobenzoic acid (1000 mg, 4.57 mmol) in dry toluene (8 mL) was added dry DMF (10 µL) and $SOCl_2$ (0.5 mL, 6.85 mmol) under argon atmosphere and the mixture was stirred under reflux conditions for 16 h. After the reaction was allowed to cool to room temperature, $SOCl_2$ was removed under reduced pressure. NaN_3 (742 mg, 11.40 mmol) was added under argon atmosphere and the mixture was stirred at room temperature for 40 minutes and subsequently heated to 90°C for 16 h. After cooling to room temperature *R*-(-)-3-quinuclidinol (871 mg, 6.85 mmol) was added under argon atmosphere and the reaction was stirred again at 120°C for 2 h. After cooling to room temperature saturated aqueous $NaHCO_3$ solution was added and the aqueous layer was extracted three times with $CHCl_3$. The combined organic layer was washed with saturated aqueous NaCl solution and dried (Na_2SO_4). The solvent was removed under reduced pressure and the residue was purified by preparative HPLC (column 1, eluent: CH_3CN / H_2O + 0.1% HCO_2H (0 - 10 min, 10 - 25 % CH_3CN, 10 - 11 min, 25 – 90 % CH_3CN, 11 – 14 min, 90 – 90 % CH_3CN)) to obtain the formate salt of **23** (1340 mg, 85 %) as a brown oil. IR (NaCl): 3410, 3357, 3309, 2925, 2872, 2849, 1717, 1595, 1524, 1489, 1237, 1189, 1055, 1029, 860, 792 cm^{-1}; ^{1}H NMR (600 MHz, DMSO-d6): δ (ppm) = 9.03 (s, 1H), 7.62 (dd, J = 8.3, 2.9 Hz, 1H), 7.49 (dd, J = 8.8, 5.8 Hz, 1H), 7.26 (td, J = 8.6, 2.9 Hz, 1H), 4.70 – 4.65 (m, 1H), 3.19 (dd, J = 14.2, 8.3 Hz, 1H), 2.83 – 2.75 (m, 2H), 2.74 – 2.64 (m, 3H), 2.02 – 1.96 (m, 1H), 1.88 – 1.68 (m, 1H), 1.67 – 1.61 (m, 1H), 1.57 – 1.50 (m, 1H), 1.42 – 1.35 (m, 1H); ^{13}C NMR (151 MHz, DMSO-d6): δ (ppm) = 159.26 (d, J = 246.7 Hz), 154.01, 133.15 (d, J = 3.2 Hz), 129.00, 119.47 (d, J = 25.5 Hz), 115.05 (d, J = 22.0 Hz), 71.05, 54.88, 46.70, 45.77, 25.02, 23.60, 18.86; HPLC (254 nm, System A): t_R = 13.99 min, HPLC (254 nm, System C): t_R = 13.31 min; ESI-MS: 345.04 $[M+H]^+$; HR-ESI-MS: calculated 343.0452, found 343.0450 $[M+H]^+$.

(1S,3R,4S)-Quinuclidin-3-yl [4-fluoro-2-(thiophen-3-yl)phenyl]carbamate **(6h)**

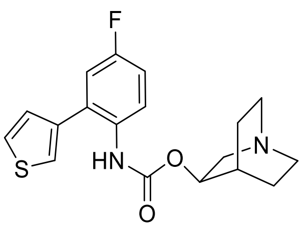

To a solution of compound **23** (200 mg, 584 µmol) in 1,4-dioxane (4 mL) was added thiophene-3-boronic acid (149 mg, 1170 µmol), 1´1[bis(diphenylphosphino)ferrocene] dichloropalladium(II) (85 mg, 117 µmol) and Na_2CO_3 (494 mg, 4680 µmol) in a microwave tube. After addition of H_2O (1 mL) the tube was sealed and the reaction mixture was stirred at 90°C for 16 h. The mixture was allowed to cool to room temperature, was filtered through celite and $MgSO_4$ and the filter was

repeatedly washed with ethyl acetate. After removing the solvent under reduced pressure, the crude product was dissolved in saturated aqueous Na_2CO_3 solution. The aqueous layer was extracted three times with $CHCl_3$ and the combined organic layer was washed with saturated aqueous NaCl solution and dried (Na_2SO_4). The organic solvent was removed under reduced pressure and the residue was purified by preparative HPLC (column 1, eluent: CH_3CN / H_2O + 0.1% HCO_2H (0 - 10 min, 10 - 25 % CH_3CN, 10 - 11 min, 25 – 90 % CH_3CN, 11 – 14 min, 90 – 90 % CH_3CN)) to obtain the formate salt of **6h** as a colorless oil (99 mg, 49 %). ^{1}H NMR (600 MHz, Methanol-d4): δ (ppm) = 7.54 – 7.46 (m, 2H), 7.46 – 7.38 (m, 1H), 7.38 – 7.23 (m, 2H), 7.23 – 7.10 (m, 1H), 7.10 – 7.06 (m, 1H), 4.97 (s, 1H), 3.74 – 3.54 (m, 1H), 3.43 – 3.35 (m, 1H), 3.30 – 3.02 (m, 4H), 2.39 – 2.17 (m, 1H), 2.16 – 1.82 (m, 4H); ^{13}C-NMR (151 MHz, DMSO-d6): δ (ppm) = 163.73, 160.07 (d, J = 242.3 Hz), 154.15, 138.14, 130.77 (d, J = 2.6 Hz), 127.91, 126.22, 124.00, 119.86 (d, J = 5.1 Hz), 116.00 (d, J = 23.1 Hz), 114.25 (d, J = 22.2 Hz), 69.10, 53.74, 46.04, 45.17, 24.44, 21.95, 17.84; HPLC (254 nm, System A): t_R = 15.39 min, purity: 97 %, HPLC (254 nm, System C): t_R = 14.67 min, purity: 98 %; ESI-MS: 347.11 $[M+H]^+$; HR-ESI-MS: calculated 347.1224, found 347.1221 $[M+H]^+$.

(1S,3R,4S)-Quinuclidin-3-yl [4-fluoro-2-(thiophen-2-yl)phenyl]carbamate **(6i)**

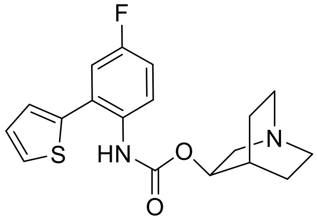

(1*S*,3*R*,4*S*)-Quinuclidin-3-yl [4-fluoro-2-(thiophen-2-yl)phenyl]carbamate **(6i)** was prepared according to the protocol of **6h**, using a solution of compound **23** (200 mg, 583 µmol) in 1,4-dioxane (8 mL), thiophene-2-boronic acid (149 mg, 1170 µmol), 1′1[bis(diphenylphosphino) ferrocene] dichloropalladium(II) (85.28 mg, 116.56 µmol), Na_2CO_3 (494.12 mg, 4660 µmol) and H_2O (2 mL). Purification by preparative HPLC (column 1, eluent: CH_3CN / H_2O + 0.1% HCO_2H (0 - 10 min, 10 - 25 % CH_3CN, 10 - 11 min, 25 – 90 % CH_3CN, 11 – 14 min, 90 – 90 % CH_3CN)) obtained the formate salt of **6i** as a yellow brown oil (90 mg, 45 %). IR (NaCl): 3360, 3193, 3106, 2923, 2850, 2831, 2716, 1717, 1612, 1532, 1356, 1236, 1175, 1045, 772, 702 cm^{-1}; ^{1}H-NMR (400 MHz, DMSO-d6): δ (ppm) = 9.05 (s, 1H), 8.29 (s, 1H), 7.65 (dd, J = 5.1, 1.2 Hz, 1H), 7.56 – 7.48 (m, 2H), 7.33 (dd, J = 8.7, 5.7 Hz, 1H), 7.18 – 7.12 (m, 2H), 4.63 (s, 1H), 3.29 – 3.04 (m, 1H), 3.04 – 2.52 (m, 5H), 2.06 – 1.71 (m, 2H), 1.69 – 1.31 (m, 3H); ^{13}C NMR (151 MHz, DMSO-d6): δ (ppm) = 160.25 (d, J = 243.1 Hz), 154.55, 138.46, 133.13 (d, J = 5.2 Hz), 130.96 (d, J = 7.3 Hz), 130.16 (d, J = 2.6 Hz), 127.58, 127.23, 126.95, 114.83 (d, J = 23.6 Hz), 114.47 (d, J = 22.3 Hz), 70.32, 54.43, 46.32, 45.37, 24.91, 23.16, 18.60; HPLC (254 nm, System A): t_R = 15.48 min, purity: 98 %, HPLC (254 nm, System C): t_R = 14.33 min, purity: 99 %; ESI-MS: 347.12 $[M+H]^+$; HR-ESI-MS: calculated 347.1224, found 347.1223 $[M+H]^+$.

(1S,3R,4S)-3-[({4-Fluoro-2-(thiophen-3-yl)phenyl}carbamoyl)oxy]-1-methylquinuclidin-1-ium formate **(6l)**

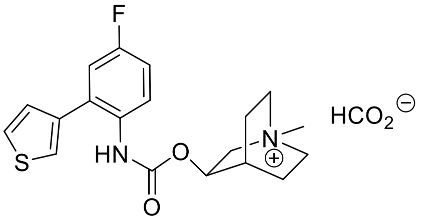

To a solution of compound **6h** (25 mg, 72 µmol) in dry CH_2Cl_2 (2 mL) was added K_2CO_3 (30 mg, 217 µmol) and methyliodide (11 µL, 180 µmol) under argon atmosphere. The reaction was stirred at room temperature for 1 h. The solvent was removed under reduced pressure and the residue was purified by preparative HPLC (column 1, eluent: CH_3CN / H_2O + 0.1% HCO_2H (0 - 10 min, 20 - 25 % CH_3CN, 10 - 11 min, 25 – 90 % CH_3CN, 11 – 14 min, 90 – 90 % CH_3CN)) to afford **6l** as a yellow oil (14 mg, 47 %). IR (NaCl): 3360, 3190, 3000, 2921, 2851, 2831, 2715, 1717, 1593, 1538, 1356, 1244, 1170, 1037, 772, 702, 652 cm^{-1}; ^{1}NMR (400 MHz, DMSO-d6): δ (ppm) = 9.56 (s, 1H), 8.56 (s, 1H), 7.82 – 7.75 (m, 1H), 7.68 – 7.62 (m, 1H), 7.44 – 7.36 (m, 2H), 7.35 – 7.29 (m, 1H), 7.18 (td, J = 8.4, 2.9 Hz, 1H), 4.84 (s, 1H), 3.88 – 3.74 (m, 1H), 3.60 – 3.10 (m, 5H), 2.96 (s, 3H), 2.27 – 2.10 (m, 1H), 2.03 – 1.46 (m, 4H); ^{13}C NMR (101 MHz, DMSO-d6): δ (ppm) = 160.09 (d, J = 242.9 Hz), 153.79, 138.04, 134.48, 130.48 (d, J = 2.7 Hz), 130.20, 128.02, 126.39, 124.21, 116.10 (d, J = 23.1 Hz), 114.34 (d, J = 22.3 Hz), 67.45, 62.13, 55.72, 54.98, 50.88, 23.47, 20.63, 17.89; HPLC (254 nm, System A): t_R = 15.38 min, purity: 96 %, HPLC (254 nm, System C): t_R = 14.80 min, purity: 96 %; ESI-MS: 361.09 $[M]^+$; HR-ESI-MS: calculated 361.1381, found 361.1383 $[M]^+$.

(1S,3R,4S)-3-[({4-Fluoro-2-(thiophen-2-yl)phenyl}carbamoyl)oxy]-1-methylquinuclidin-1-ium formate **(6m)**

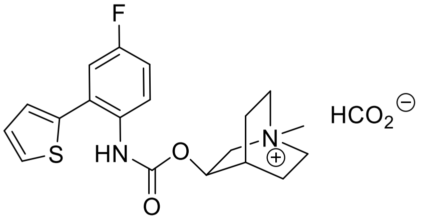

(1*S*,3*R*,4*S*)-3-[({4-Fluoro-2-(thiophen-2-yl)phenyl}carbamoyl)oxy]-1-methylquinuclidin-1-ium formate **(6m)** was prepared according to the protocol of **6l**, using a solution of compound **6i** (15 mg, 43 µmol) in dry CH_2Cl_2 (2 mL), K_2CO_3 (18 mg, 130 µmol) and iodomethane (10 µL, 160 µmol). Purification by preparative HPLC (column 1, eluent: CH_3CN / H_2O + 0.1% HCO_2H (0 - 10 min, 20 - 35 % CH_3CN, 10 - 11 min, 25 – 90 % CH_3CN, 11 – 14 min, 90 – 90 % CH_3CN)) obtained **6m** as a red brown oil (11 mg, 68 %). ^{1}H NMR (400 MHz, Methanol-d4): δ (ppm) = 8.97 (s, 1H), 7.56 – 7.51 (m, 1H), 7.51 – 7.42 (m, 1H), 7.42 – 7.24 (m, 2H), 7.15 (dd, J = 5.2, 3.6 Hz, 1H), 7.09 (ddd, J = 8.8, 7.9, 2.9 Hz, 1H), 5.05 (s, 1H), 3.92 – 3.76 (m, 1H), 3.61 – 3.32 (m, 5H), 3.00 (s, 3H), 2.60 – 2.18 (m, 2H), 2.18 – 1.84 (m, 3H). ^{13}C NMR (151 MHz, DMSO-d6): δ (ppm) = 165.36, 160.32 (d, J = 244.4 Hz), 154.04, 138.36 , 132.94 (d, J = 7.4 Hz), 129.81, 127.71, 127.41, 127.17, 119.85 (d, J = 5.1 Hz), 115.05 (d, J = 22.8 Hz), 114.62 (d, J = 22.3 Hz), 67.53, 62.14, 55.76, 55.01, 50.93, 23.59, 20.64, 17.90. HPLC (254 nm, System A): t_R = 15.66

min, purity: 98 %, HPLC (254 nm, System C): t_R = 14.57 min, purity: 95 %; ESI-MS: 361.12 $[M]^+$, HR-ESI-MS: calculated 361.1381, found 361.1378 $[M]^+$.

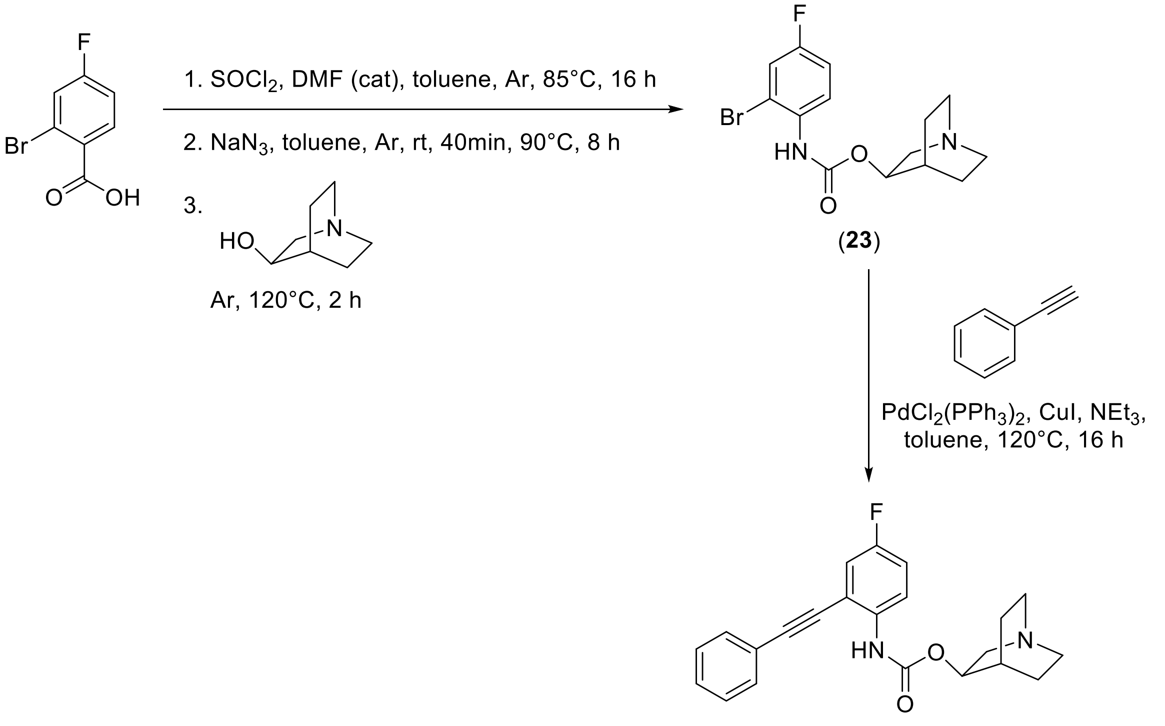

(**6j**)

(1S,3R,4S)-Quinuclidin-3-yl [4-fluoro-2-(phenylethynyl)phenyl]carbamate **(6j)**

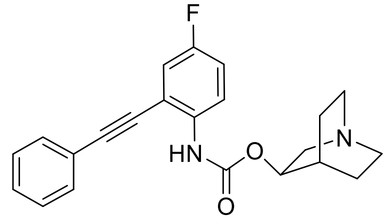

To a solution of compound **23** (50 mg, 146 µmol) in dry toluene (4 mL) was added phenylacetylene (45 mg, 437 µmol), copper iodide (2.8 mg, 15 µmol), bis(triphenylphosphine)palladium(II) dichloride (21 mg, 29 µmol) and triethylamine (44 mg, 437 µmol) under argon atmosphere in a microwave tube. The tube was sealed and the reaction mixture was stirred for 16 h at 120°C. After allowing the reaction to cool to room temperature, saturated, aqueous $NaHCO_3$ solution was added and the aqueous layer was extracted three times with $CHCl_3$. The combined organic layer was washed with saturated, aqueous NaCl solution and dried over Na_2SO_4. After removing the organic solvent under reduced pressure the crude product was purified by preparative HPLC (column 1, eluent: CH_3CN / H_2O + 0.1% TFA (0 - 10 min, 10 - 100 % CH_3CN, 10 - 12 min, 100 – 100 % CH_3CN)) to obtain the trifluoroacetate salt of **6j** as a white lyophilisate (28 mg, 53 %); ^1H NMR (600 MHz, Methanol-d4): δ (ppm) = 7.80 – 7.61 (m, 1H), 7.59 – 7.55 (m, 2H), 7.44

– 7.38 (m, 3H), 7.28 (dd, J = 8.8, 3.0 Hz, 1H), 7.14 (ddd, J = 9.0, 8.1, 3.0 Hz, 1H), 5.15 – 5.06 (m, 1H), 3.80 – 3.71 (m, 1H), 3.36 (d, J = 14.0 Hz, 3H), 3.30 – 3.18 (m, 2H), 2.48 – 2.36 (m, 1H), 2.36 – 2.10 (m, 1H), 2.09 – 2.02 (m, 1H), 2.00 – 1.84 (m, 2H); ^{13}C NMR (151 MHz, Methanol-d4): δ (ppm) = 160.66 (d, J = 238.1 Hz), 155.15, 136.12 (d, J = 2.8 Hz), 132.68, 130.23, 129.71, 123.67, 119.34 (d, J = 24.4 Hz), 117.30 (d, J = 22.7 Hz), 96.67, 85.08, 69.13, 54.53, 47.67, 46.85, 25.38, 21.08, 17.83; HPLC (254 nm, System A): t_R = 17.53 min, purity: 95 %, HPLC (254 nm, System C): t_R = 16.65 min, purity: 95 %; ESI-MS: 365.17 $[M+H]^+$; HR-ESI-MS: calculated 365.1660, found 365.1655 $[M+H]^+$.

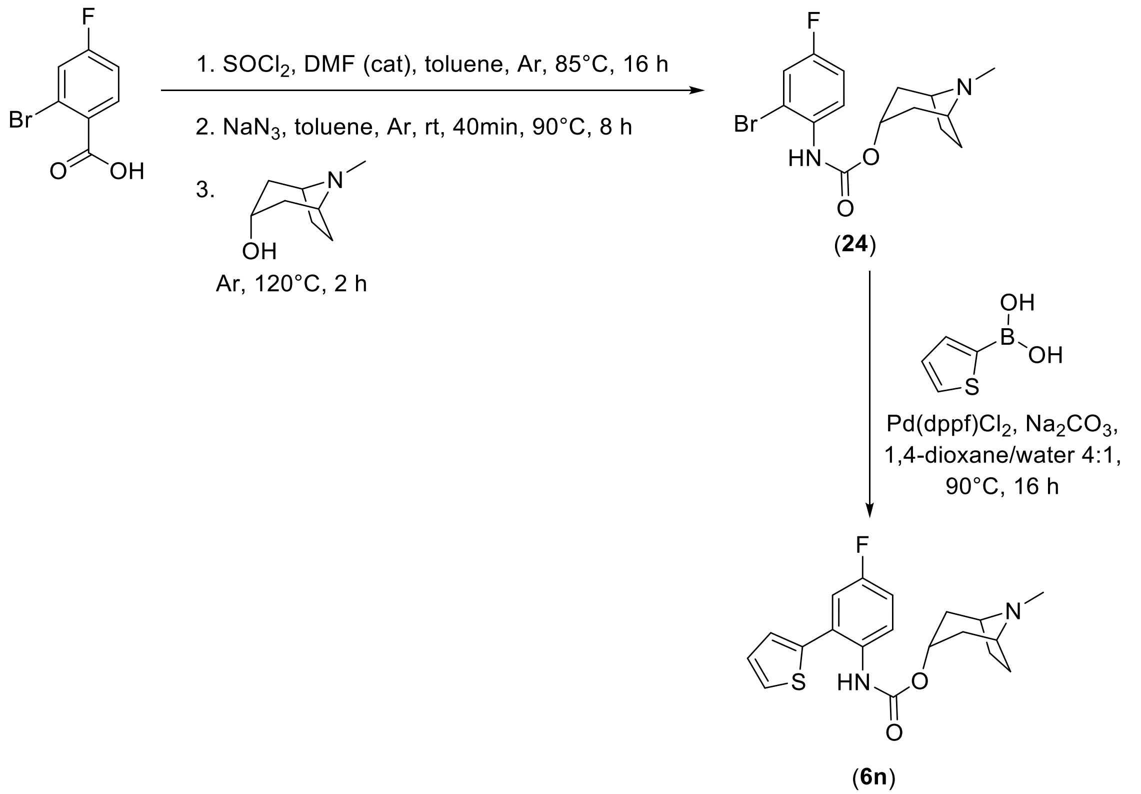

(1R,3R,5S)-8-Methyl-8-azabicyclo[3.2.1]octan-3-yl (2-bromo-4-fluorophenyl)carbamate **(24)**

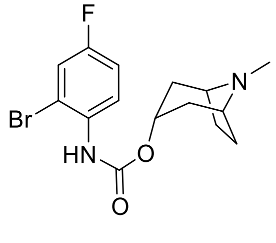

(1*R*,3*R*,5*S*)-8-Methyl-8-azabicyclo[3.2.1]octan-3-yl (2-bromo-4-fluorophenyl)carbamate **(24)** was prepared according to the protocol of **23**, using a solution of 2-bromo-4-fluorobenzoic acid (1000 mg, 4.57 mmol) in dry toluene (10 mL), DMF (10 µL), $SOCl_2$ (0.67 mL, 9.13 mmol), NaN_3 (742 mg, 11.42 mmol) and tropine (967 mg, 6.85

mmol). Purification by preparative HPLC (column 3, eluent: CH_3OH / H_2O + 0.1% HCO_2H (0 - 10 min, 10 - 90 % CH_3OH, 10 - 12 min, 90 – 90 % CH_3OH)) afforded the formate salt of **24** (729 mg, 44 %) as a brown oil. IR (NaCl): 3415, 3311, 3156, 2928, 2852, 2803, 2539, 2161, 2017, 1713, 1595, 1524, 1485, 1220, 1189, 1051, 856, 793, 662 cm^{-1}; ^{1}H-NMR (400 MHz, DMSO-d6): δ (ppm) = 9.14 (s, 1H), 7.65 (dd, J = 8.3, 2.9 Hz, 1H), 7.49 (dd, J = 8.9, 5.7 Hz, 1H), 7.28 (td, J = 8.5, 2.9 Hz, 1H), 4.83 (t, J = 4.9 Hz, 1H), 3.69 (s, 2H), 2.58 (s, 3H), 2.38 – 2.28 (m, 2H), 2.28 – 1.94 (m, 4H), 1.94 – 1.78 (m, 2H); ^{13}C-NMR (151 MHz, DMSO-d6): δ (ppm) = 159.55 (d, J = 249.4 Hz), 153.47, 133.01 (d, J = 2.9 Hz), 129.62, 119.53 (d, J = 25.5 Hz), 115.14 (d, J = 22.1 Hz), 65.17, 60.53 (2C), 37.67, 33.94 (2C), 23.95 (2C); HPLC (254 nm, System A): t_R = 14.34 min, HPLC (254 nm, System C): t_R = 13.78 min; ESI-MS: 359.00 $[M+H]^+$; HR-ESI-MS: calculated 357.0608, found 357.0606 $[M+H]^+$.

(1R,3R,5S)-8-Methyl-8-azabicyclo[3.2.1]octan-3-yl [4-fluoro-2-(thiophen-2-yl)phenyl] carbamate **(6n)**

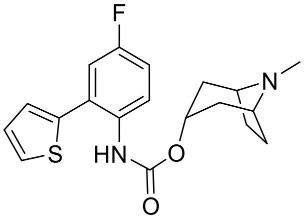

(1*R*,3*R*,5*S*)-8-Methyl-8-azabicyclo[3.2.1]octan-3-yl [4-fluoro-2-(thiophen-2-yl)phenyl] carbamate **(6n)** was prepared according to the protocol of **6h**, using a solution of compound **24** (100 mg, 280 µmol) in 1,4-dioxane (4 mL), thiophene-2-boronic acid (72 mg, 560 µmol), 1′1[bis(diphenylphosphino) ferrocene]dichloropalladium(II) (41 mg, 56 µmol), Na_2CO_3 (89 mg, 840 µmol) and H_2O (1 mL). Purification by preparative HPLC (column 3, eluent: CH_3OH / H_2O + 0.1% HCO_2H (0 - 10 min, 10 - 90 % CH_3OH, 10 - 12 min, 90 – 90 % CH_3OH)) obtained the formate salt of **6n** (101 mg, 86 %) as a yellow oil. IR (NaCl): 3406, 3312, 3196, 3053, 2961, 2925, 2851, 2831, 2715, 2550, 1704, 1593, 1520, 1441, 1355, 1226, 1172, 1041, 738, 703, 611 cm^{-1}; ^{1}H-NMR (400 MHz, DMSO-d6): δ (ppm) = 9.00 (s, 1H), 7.65 (dd, J = 5.1, 1.0 Hz, 1H), 7.61 – 7.45 (m, 2H), 7.34 (dd, J = 8.7, 5.7 Hz, 1H), 7.19 – 7.11 (m, 2H), 4.75 (s, 1H), 3.55 – 3.12 (m, 2H), 2.39 (s, 3H), 2.28 – 1.95 (m, 4H), 1.95 – 1.27 (m, 4H); ^{13}C-NMR (151 MHz, DMSO-d6): δ (ppm) = 160.29 (d, J = 241.7 Hz), 154.20, 138.41, 130.93, 130.13, 127.63, 127.24, 127.02, 114.77, 114.47 (d, J = 22.3 Hz), 65.81, 59.69 (2C), 38.32, 34.62 (2C), 24.54 (2C); HPLC (254 nm, System A): t_R = 15.59 min, purity: 97 %, HPLC (254 nm, System C): t_R = 14.64 min, purity: 96 %; ESI-MS: 361.10 $[M+H]^+$; HR-ESI-MS: calculated 361.1381, found 361.1378 $[M+H]^+$.

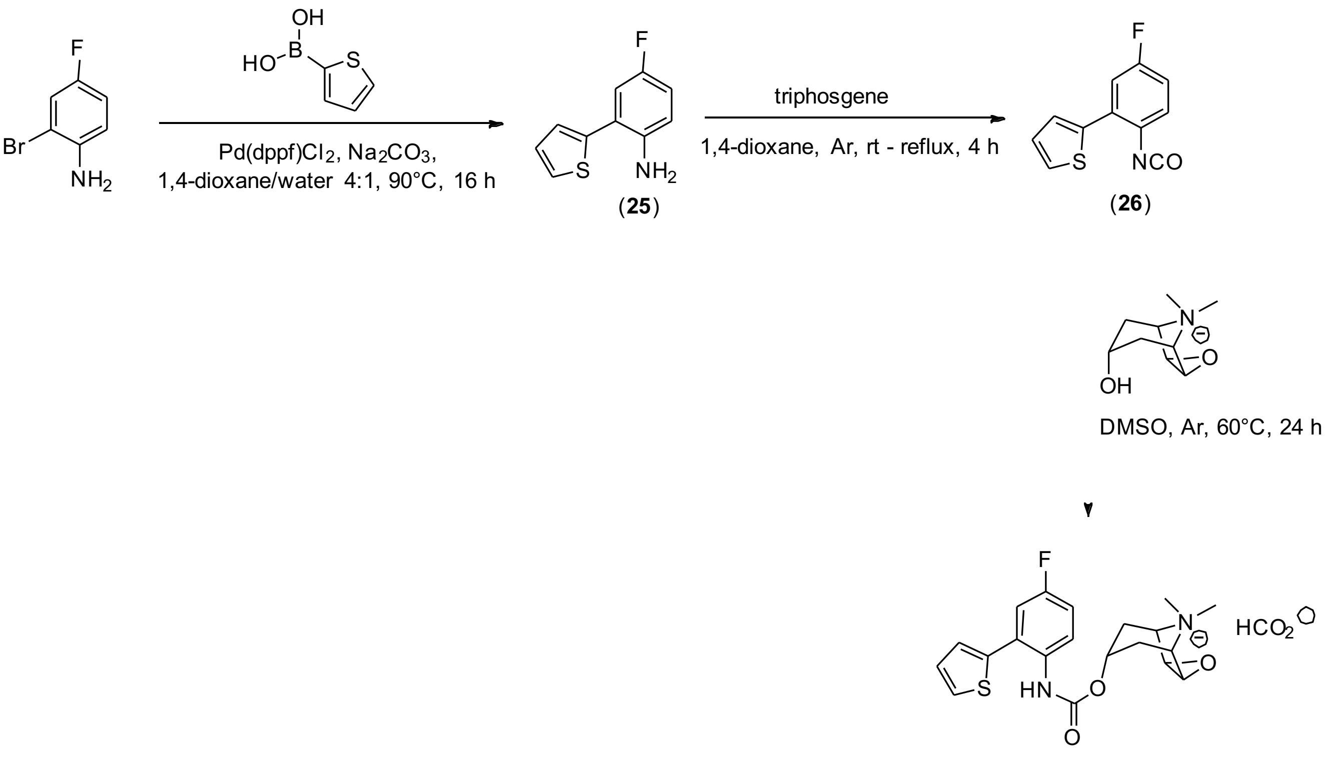

(**6o, BS46**)

4-Fluoro-2-(thiophen-2-yl)-aniline **(25)**

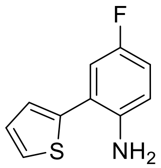

4-Fluoro-2-(thiophen-2-yl)aniline **(25)** was prepared according to the procedure of **6h** using a solution of 2-bromo-4-fluoroaniline (1000 mg, 5.26 mmol) in 1,4-dioxane (10 mL), thiophene-2-boronic acid (2 eq), 1´1[bis(diphenylphosphino)ferrocene] dichloropalladium(II) (0.2 eq), K_2CO_3 (2.5 eq) and H_2O (2.5 mL). Purification by column chromatography on silica gel (hexane/ethyl acetate 9:1) afforded **25** (709 mg, 70 %) as a red oil. ^{1}H-NMR (400 MHz, Chloroform-d): δ (ppm) = 7.37 (dd, *J* = 5.2, 1.2 Hz, 1H), 7.22 (dd, *J* = 3.5, 1.2 Hz, 1H), 7.12 (dd, *J* = 5.1, 3.5 Hz, 1H), 7.02 (dd, *J* = 9.3, 2.9 Hz, 1H), 6.87 (ddd, *J* = 8.8, 8.0, 3.0 Hz, 1H), 6.70 (dd, *J* = 8.8, 4.9 Hz, 1H), 3.87 (s, 2H); ^{13}C NMR (101 MHz, Chloroform-d): δ (ppm) = 155.95 (d, J = 236.4 Hz), 140.07 (d, J = 2.1 Hz), 139.93 (d, J = 2.0 Hz), 127.64 , 126.21 , 125.71 , 120.92 (d, J = 7.7 Hz), 116.90 (d, J = 23.0 Hz), 116.80 (d, J = 7.9 Hz), 115.50 (d, J = 22.3 Hz). HPLC (254 nm, System A): t_R = 19.03 min; HPLC (254 nm, System C): t_R = 14.42 min; ESI-MS: m/z = 193.84 [M+H]$^+$.

4-Fluoro-2-(thiophen-2-yl)-phenylisocyanate **(26)**

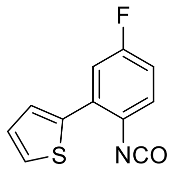

A solution of compound **25** (400 mg, 2.07 mmol) in dry 1,4-dioxane (1 mL) was added dropwise over 30 min at room temperature and argon atmosphere to a solution of triphosgene (0.4 eq) in dry 1,4-dioxane (4 mL). The reaction mixture was stirred under reflux for 4 h. The solvent was removed under reduced pressure and the residue was distilled using vacuo distillation to obtain compound **26** (202 mg, 45%) as a pale yellow oil. IR (NaCl): 3109, 3076, 2275, 1534, 1516, 1266, 837, 704; ^{1}H NMR (400 MHz, Chloroform-*d*): δ (ppm) = 7.44 (dd, *J* = 5.1, 1.2 Hz, 1H), 7.37 (dd, *J* = 3.6, 1.2 Hz, 1H), 7.28 – 7.23 (m, 1H), 7.19 – 7.12 (m, 2H), 6.98 (ddd, *J* = 8.8, 7.6, 2.9 Hz, 1H); ^{13}C NMR (101 MHz, Chloroform-*d*): δ (ppm) = 160.03 (d, *J* = 246.4 Hz), 137.99 (d, *J* = 2.1 Hz), 131.14 (d, *J* = 8.7 Hz), 127.75 (d, *J* = 8.8 Hz), 127.51 , 127.25 , 127.08 , 126.54 , 124.67 , 116.41 (d, *J* = 23.9 Hz), 115.47 (d, *J* = 23.1 Hz); HPLC (254 nm, System A): t_R = 18.76 min; HPLC (254 nm, System C): t_R = 18.04 min; ESI-MS: m/z = 219.79 [M+H]$^+$.

(1R,2R,4S,5S,7S)-7-Hydroxy-9,9-dimethyl-3-oxa-9-azatricyclo[3.3.1.0^{2,4}]nonan-9-ium bromide
(*N*-methylscopinium bromide)

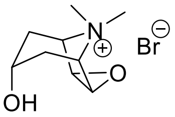

To a suspension of methylscopolamine bromide (1000 mg, 2.51 mmol) in dry methanol (3.7 mL) was added a solution of sodium methoxide (0.07 eq, 1.3 mL, 0.65 mmol, 0.5M in MeOH) under argon atmosphere and the mixture was heated to 40°C. The resulting colorless solution was stirred at 40°C for 8 h. A solution of acetic acid (17 µL in 170 µL isopropyl alcohol) was added and stirred for 10 minutes. Subsequently IPAC (13 mL) was added to give a suspension that was stirred at 40°C for 30 minutes. The reaction was allowed to cool to room temperature and was stirred overnight. The mixture was left to settle and was filtered. The solid was washed with IPAC and dried in vacuo(19) to give *N*-methylscopinium bromide (538 mg, 86%) as white solid. IR (NaCl): 3360, 3307, 3000, 2922, 1660, 1633, 1469, 1086, 1035, 803, 704; ^{1}H NMR (400 MHz, DMSO-d6): δ (ppm) = 5.09 (s, 1H), 4.13 (d, J = 3.3 Hz, 2H), 4.04 (s, 2H), 3.95 (t, J = 5.3 Hz, 1H), 3.27 (s, 3H), 2.99 (s, 3H), 2.50 – 2.40 (m, 2H), 1.80 (d, J = 16.6 Hz, 2H); ^{13}C NMR (151 MHz, DMSO-d6): δ (ppm) = 65.60 (2C), 56.89, 55.24 (2C), 54.21, 46.94, 31.47 (2C); HPLC (230 nm, System A): t_R = 3.34 min; HPLC (230 nm, System C): t_R = 2.95 min; ESI-MS: m/z = 169.81 [M]$^+$.

(1R,2R,4S,5S,7S)-7-[({4-Fluoro-2-(thiophen-2-yl)phenyl}carbamoyl)oxy]-9,9-dimethyl-3-oxa-9-azatricyclo[3.3.1.0^{2,4}]nonan-9-ium formate **(6o, BS46)**

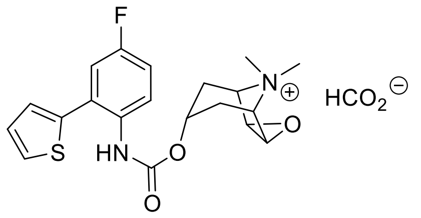

To a solution of compound **26** (100 mg, 456.13 µmol) in dry DMSO (3.5 mL) was added *N*-methylscopinium bromide (228 mg, 912 µmol) under argon atmosphere and the mixture was stirred at 60°C for 24 h. The solvent was removed under reduced pressure and the residue was purified by preparative HPLC (column 1, eluent: CH_3CN / H2O + 0.1% HCO_2H (0 – 12.5 min, 10 – 60 % CH_3CN, 12.5 – 14 min, 60 – 90 % CH_3CN, 14 – 17 min, 90 – 90%) to yield **6o (BS46)** as a pale yellow lyophilisate (72.7 mg, 37 %). IR (NaCl): 3360, 3210, 3000, 2921, 2851, 1717, 1634, 1521, 1470, 1353, 1260, 1234, 1203, 1076, 1049, 858, 803, 703 cm^{-1}; ^{1}H-NMR (400 MHz, DMSO-d6): δ (ppm) = 9.21 (s, 1H), 7.67 (d, J = 5.0 Hz, 1H), 7.64 – 7.45 (m, 2H), 7.45 – 7.31 (m, 1H), 7.30 – 7.05 (m, 2H), 4.91 (s, 1H), 4.43 – 3.92 (m, 4H), 3.29 (s, 3H), 3.02 (s, 3H), 2.78 – 2.62 (m, 2H), 2.05 – 1.58 (m, 2H); ^{13}C-NMR (151 MHz, DMSO-d6): δ (ppm) = 170.89, 163.10 (d, J = 254.2 Hz), 153.53, 138.21, 136.42, 133.11 (d, J = 9.6 Hz), 130.52, 129.79, 128.37, 127.33, 127.17, 115.13 (d, J = 21.5 Hz), 114.51 (d, J = 22.5 Hz), 64.92 (2C), 61.51, 55.58 (2C), 52.59, 47.02, 28.83 (2C); HPLC (254 nm, System A): t_R = 15.47 min, purity: 96 %, HPLC (254 nm, System C): t_R = 14.83 min, purity: 95 %; ESI-MS: 388.95 [M]$^+$; HR-ESI-MS: calculated 389.1330, found 389.1326 [M]$^+$.

Supp. Figure S1. Structural modifications of muscarinic antagonists.

Radioligand binding curves and Ki values of muscarinic ligands at M2R (blue) and M3R (red). Enlargement of the upward-directed aryl moiety of methyl-, dimethyl-, benztiotropium and the QNB-based analogs **1a-1e** did not lead to substantial M3R selectivity over M2R. The biphenyl derivative **2** (structural intermediate on the design of M3 selective antagonists of type **4**, **5** and **6,** see **Fig. S5**) showed also poor M3 selectivity. The carbamate **3** (structural intermediate on the design of M3 selective antagonists of type **4** and **6,** see **Fig. S5**) revealed a 13-fold selectivity for M3R over M2R. Graphs represent mean curves (±SEM) from 4-8 individual experiments each done in triplicate.

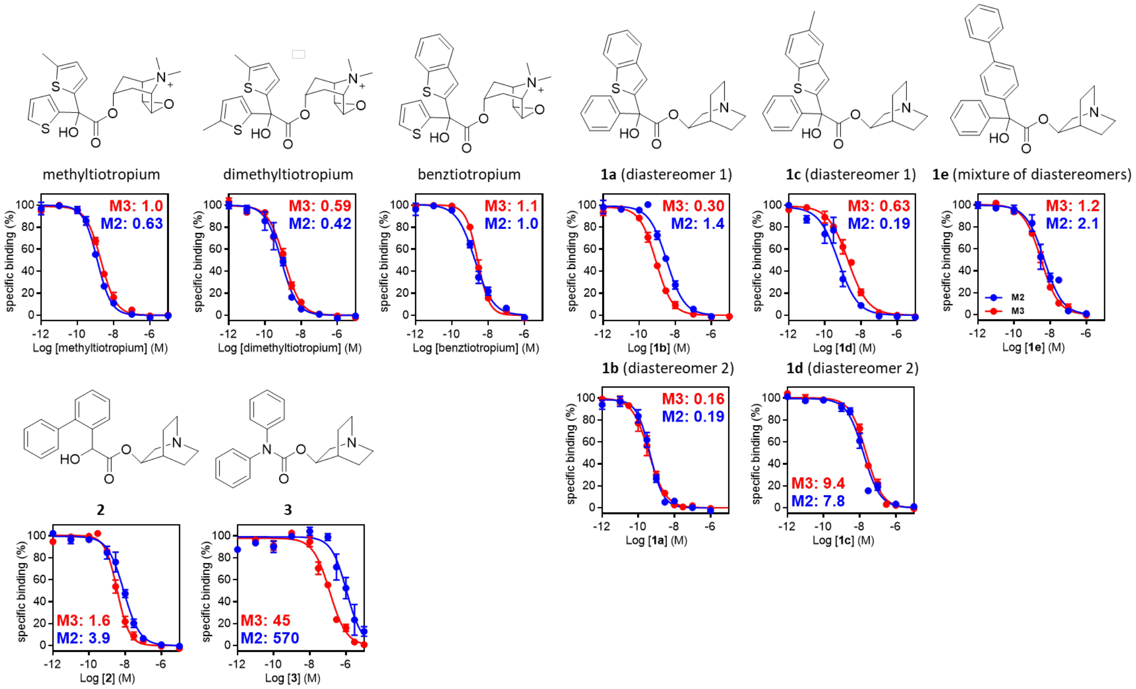

Supp. Figure S2. Docked binding poses of compounds 1c, *(R)*2, *(S)*2, 3, 4, 5a and 5b. M2R is represented in green and M3R is represented in magenta; Asn6.52, Asp3.32 and Phe181/Leu225 residues are represented as sticks.

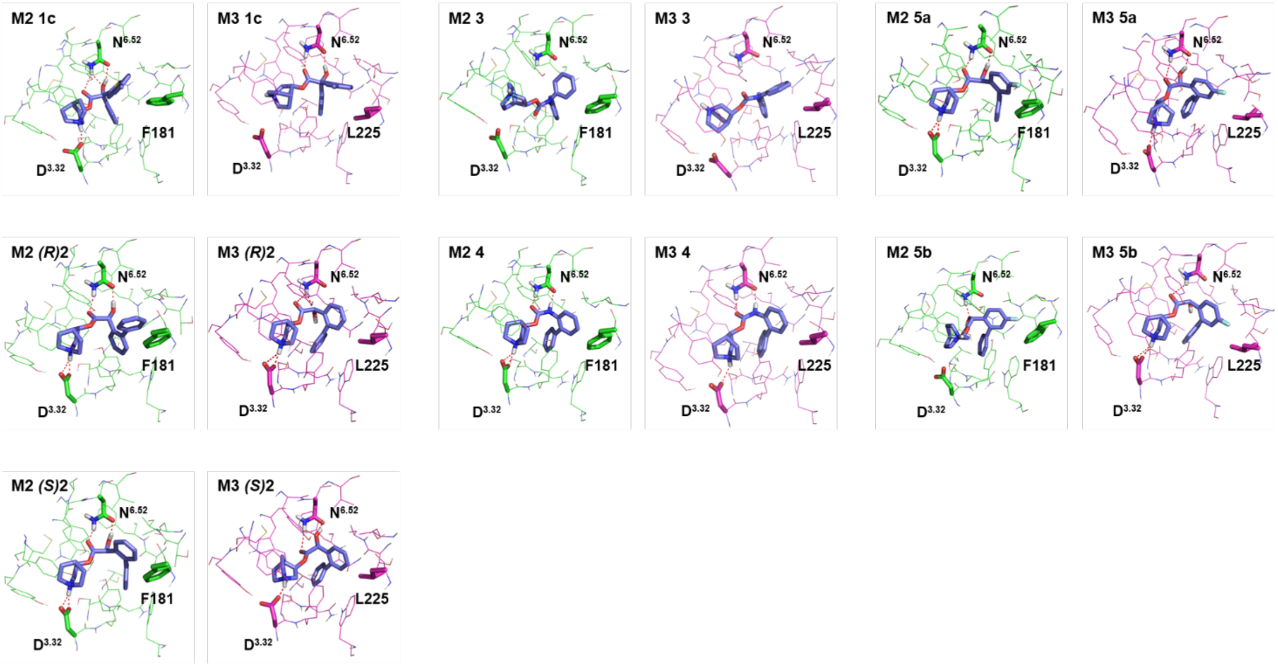

Supp. Figure S3. Docked binding poses of compounds 6a-g.

M2R is represented in green and M3R is represented in magenta; Asn6.52, Asp3.32 and Phe181/Leu225 residues are represented as sticks. In the docked pose of compound **6b**, the compound's fluorine moiety is juxtaposed against Phe181/Leu225, the only point of difference between the M3 and M2 orthosteric binding sites.

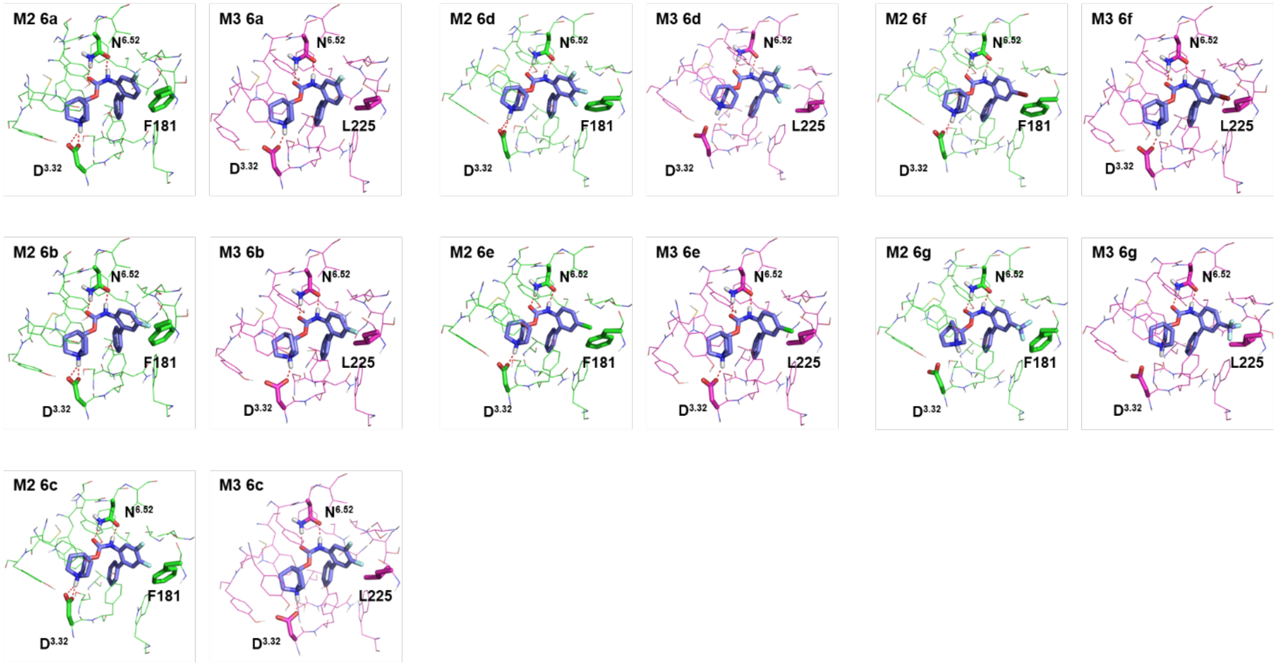

Supp. Figure S4. Hydrophobic packing of ring A of tiotropium in M3R crystal structure.

The crystal structure of M3R in complex with tiotropium (PDB entry 4U15) shows a tight packing of ring **A** of tiotropium by the hydrophobic residues $Tyr^{3.33}$, $Val^{3.40}$, $Trp^{4.57}$, $Ala^{5.46}$ and $Trp^{6.48}$ of the receptor.

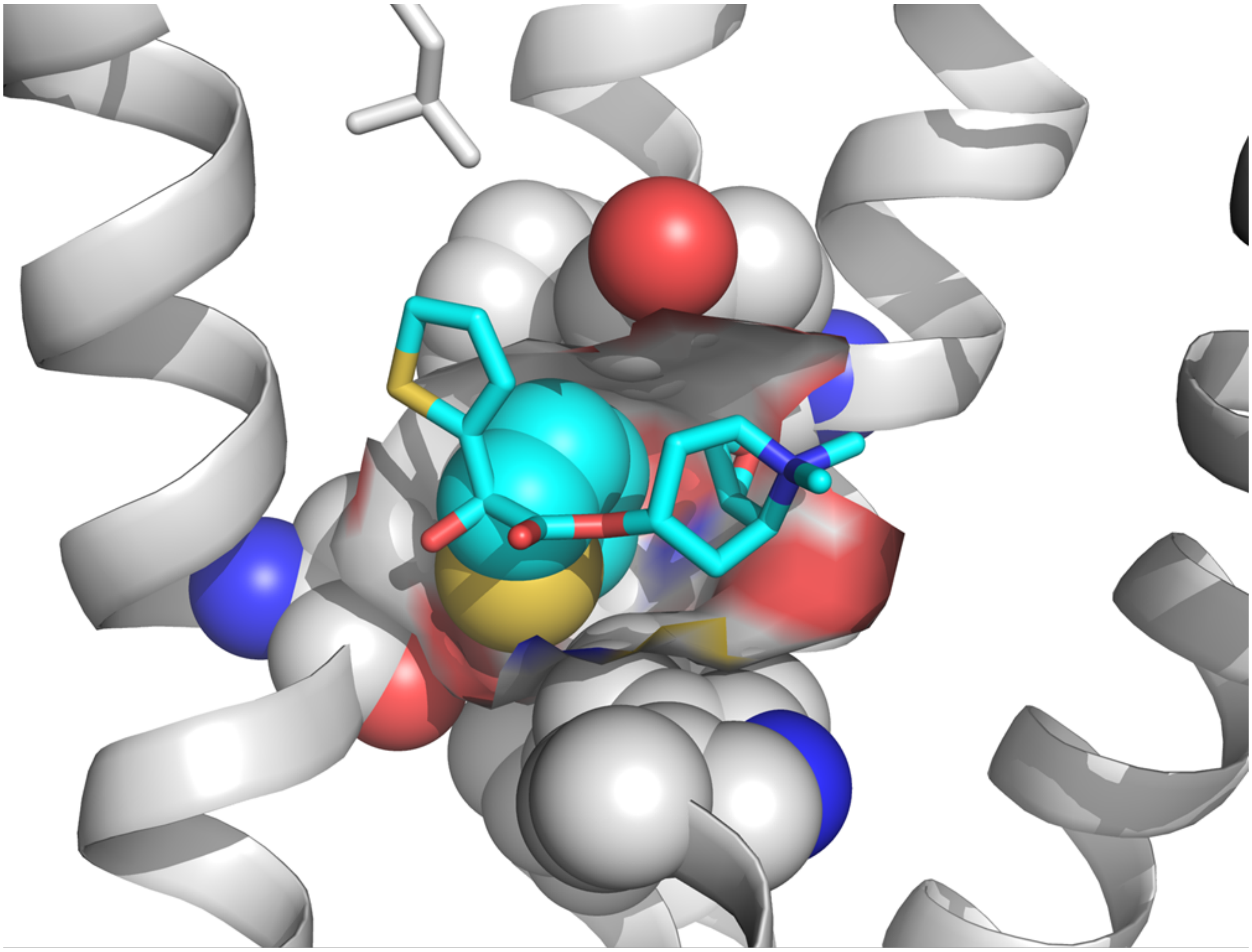

M3R – tiotropium

Supp. Figure S5. Structure-activity relationships on compounds of type 4, 5 and 6. Radioligand displacement curves (% of specific binding) for M2R (blue) and M3R (red) and Ki values for 18 compounds varying in ring **A** (orange frames), ring **B** (red frames), the core entity (blue frames) and the amine moiety (green frames) of the parent compound **4** are displayed. Modification of the substitution pattern of the upward-directed ring **B** gave high M2R affinity and selectivity, in particular when fluorine is bound to the para-position. Variation of ring **A** indicated that the introduction of a thiophene moiety was beneficial. Modification of the amine unit showed a similar impact of quinuclidine and tropine groups on M3R affinity and selectivity (**6k-6n**). Introduction of a scopinium group led to superior binding affinity (**6o, BS46)**). Mean curves (±SEM) are derived from three to eight individual experiments each done in triplicate.

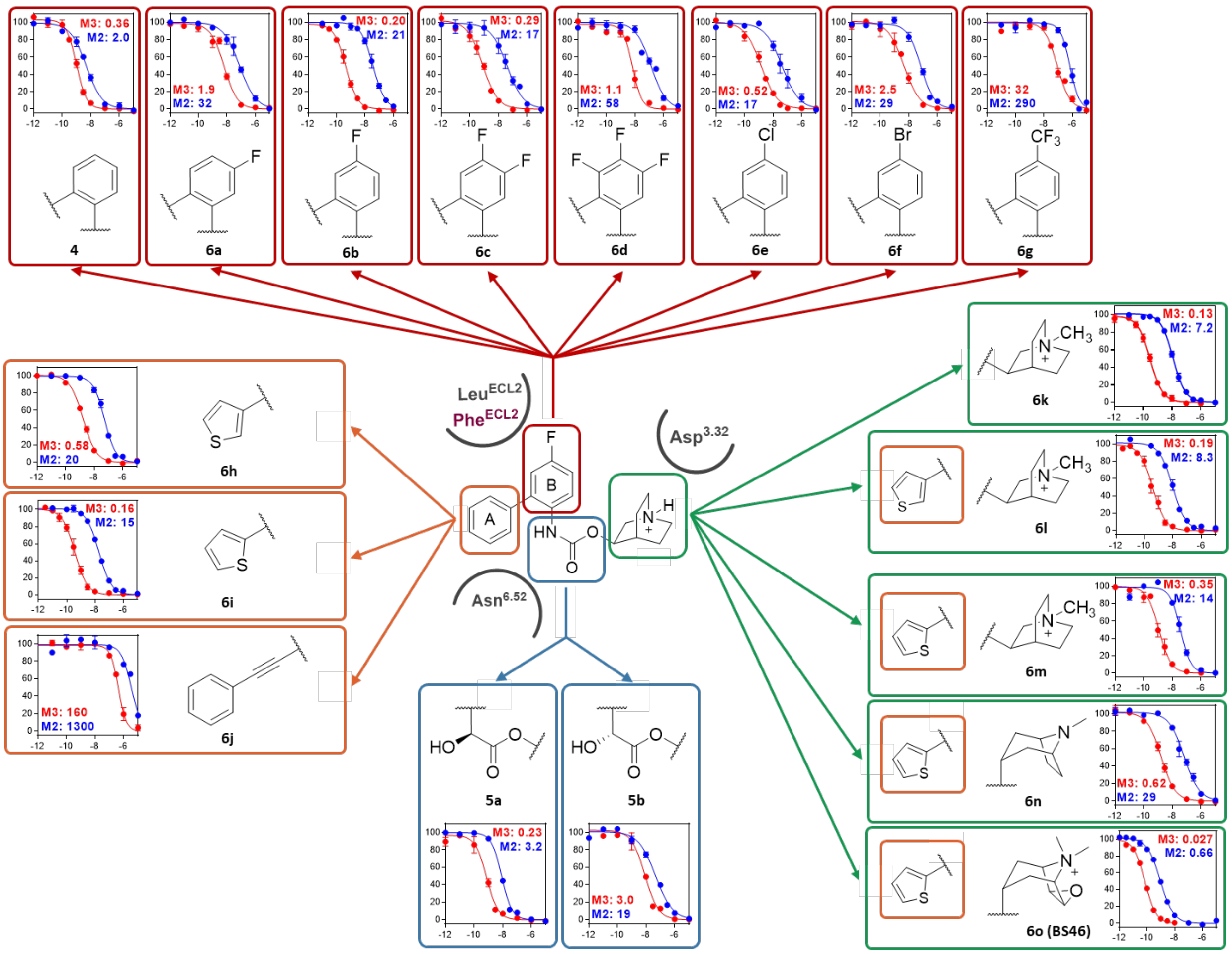

Supp. Figure S6. Functional antagonism of compound 6b and 6o (BS46).

In vitro functional properties of compounds **6b** (blue), **6o (BS46)** (red) and tiotropium (black) at the M3 receptor measured with an IP-accumulation assay (**a**) (IP-One® assay (Cisbio), M3R and the hybrid-protein G_{qi5}) and a β-arrestin recruitment assay (**b**) (Path-Hunter® assay (DiscoverX), M3R tagged with the ProLink fragment PK1). While **6b**, **6o (BS46)** and tiotropium showed no agonist properties (data not shown), a clear inhibiting effect of **6o (BS46)** on a fixed concentration of carbachol (0.1 µM for IP, 50 µM for arrestin) could be observed in both systems. This antagonism is similar to that of tiotropium and shows a stronger potency for **6o (BS46)** than for **6b**. Graphs represent mean curves (±SEM) from 8-10 single experiments each done in duplicate.

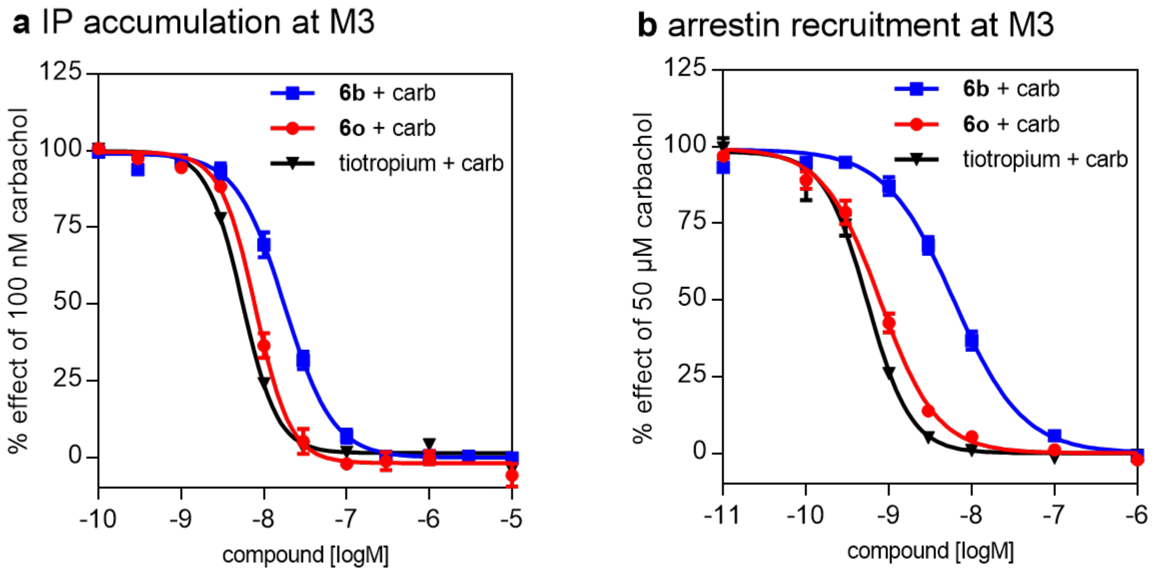

Supp. Figure S7. Selectivity over 21 aminergic and peptidergic GPCRs.

a, Receptor selectivity of compound **6o (BS46)** over representative class A GPCRs including adrenergic (blue bar), dopaminergic (red bar), serotonergic (green bar), opioid receptors (brown bar) and neurotensin receptors (NTS1, NTS2) (magenta bar) determined by radioligand binding at 10 µM of **6o (BS46)** and in a functional assay (IP accumulation) with the orexin receptors (OXR1, OXR2) and the protease-activated receptor (PAR2) (striped bar) measuring the inhibitory effect of **6o (BS46)** against the EC_{80} concentration of the standard agonist (*). Bars represent the mean displacement of reference (%±SEM) from 4-6 individual experiments each done in triplicate. Receptors showing displacement >15% (**) were investigated in a concentration-dependent manner. **b-f,** Radioligand binding curves and Ki values for **6o (BS46)** (blue curve) and tiotropium (red curve) at selected receptors. Mean curves from four experiments each done in triplicate and the data from **a** indicate the selectivity of **6o (BS46)** for M3R over all tested GPCRs of >100,000.

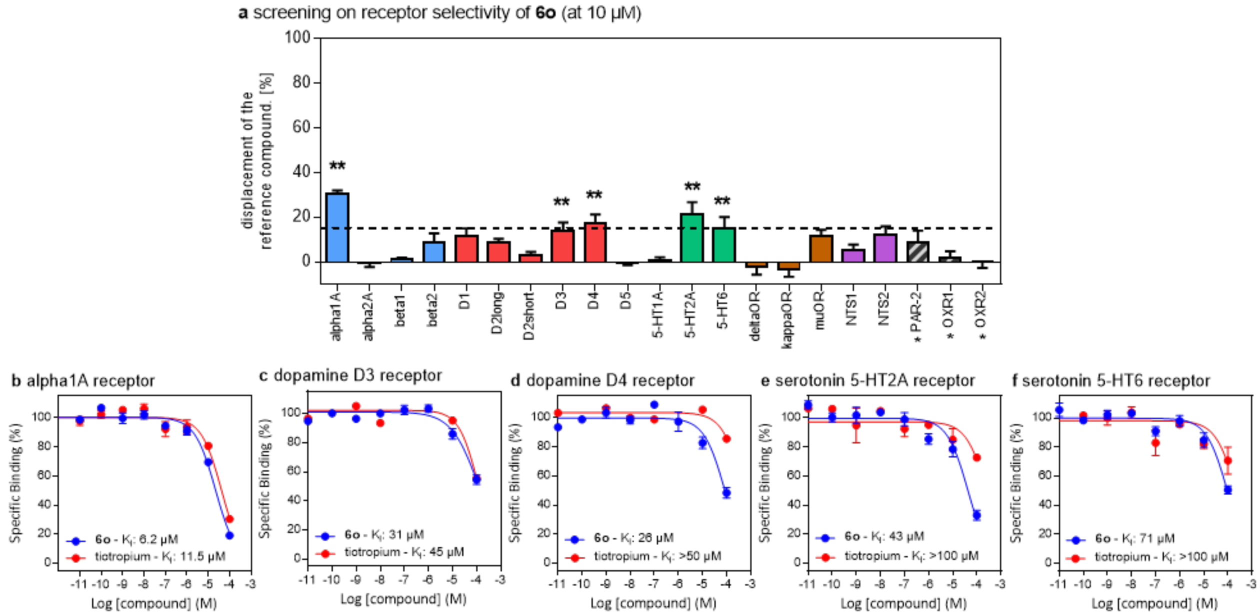

Supp. Figure S8. Dissociation/association kinetics of Compound 6o (BS46) and tiotropium at M2R and M3R.

a, Association of [^{3}H]-NMS in the absence or presence of 0.2 or 0.6 nM tiotropium in M2R membranes. **b,** Association of [^{3}H]-NMS in the absence or presence of 0.6 or 2.0 nM Compound **6o (BS46)** in M2R membranes. **c,** Association of [^{3}H]-NMS in the absence or presence of varying tiotropium concentrations (0.1, 0.4 and 1 nM) in M3R membranes. **d,** Association of [^{3}H]-NMS in the absence or presence of varying Compound **6o (BS46)** concentrations (0, 0.06, 0.2, 0.6 and 2 nM) in M3R membranes.

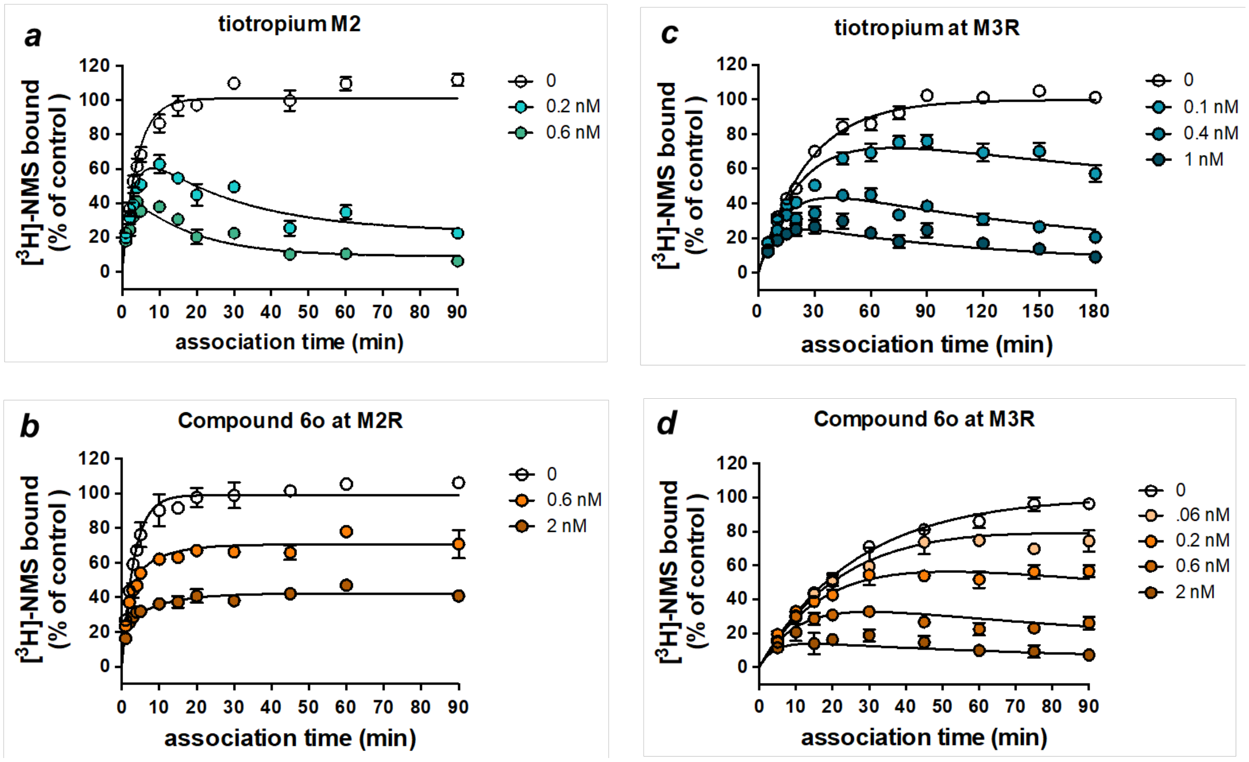

Supp. Figure S9. Mutational Analysis.

The role of Leu225^{ECL2} in M3R for receptor selectivity of the compounds **6i**, **6k**, **6l**, **6n** and **6o (BS46)** in comparison to the reference QNB was examined determining binding affinity at the mutant M3R L225F carrying the bulkier Phe at position 225 and its inverse mutant M2R F181L. While QNB binding shows no sensitivity to the mutations, compounds revealed a 7- to 48-fold reduction of binding at the M3R L225F mutant (magenta curve) compared to wild-type (red curve). A 4- to 29-fold increase of affinity was observed for M2R F181L (light blue) relative to M2 wild-type (dark blue). Graphs represent mean curves (±SEM) from three to five single experiments each done in triplicate.

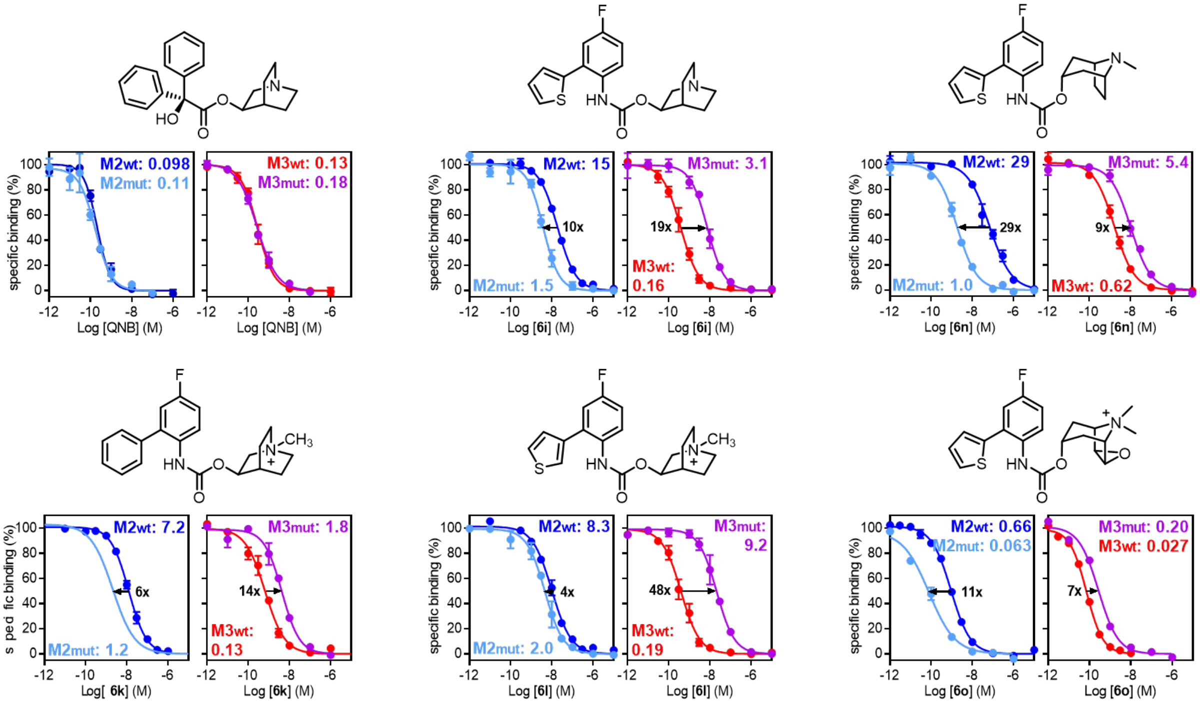

Supp. Figure S10. The F_o-F_c simulated annealing omit map of selective antagonist 6o (BS46) binding site in M3R.

a, **b**, Side and top views, respectively, of the selective antagonist **6o (BS46)** binding site in the M3R revealed in F_o-F_c simulated annealing omit map, contoured at 2.5σ (green).

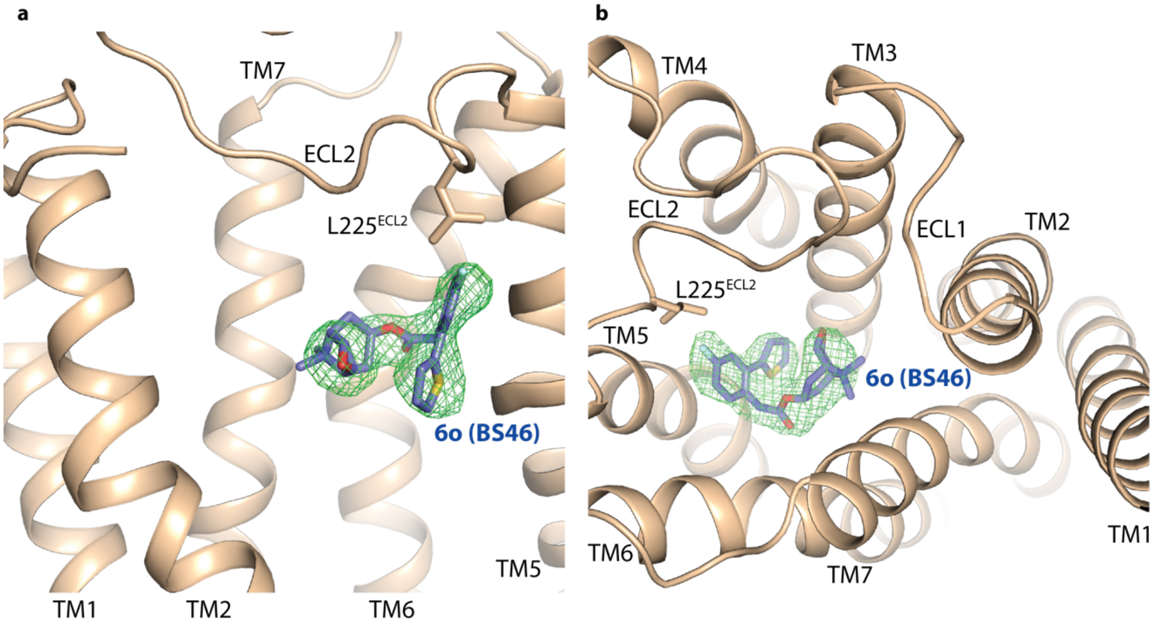

Supp. Figure S11. Comparison of 6o (BS46) binding site in X-ray structure and docking model.

a, b, The side view and top view, respectively, of **6o (BS46)** binding site in X-ray structure and docking model. X-ray structure is colored in magenta and docking model in gray. Compound **6o (BS46)** in the X-ray structure is shown as blue sticks and in the docking model as yellow sticks.

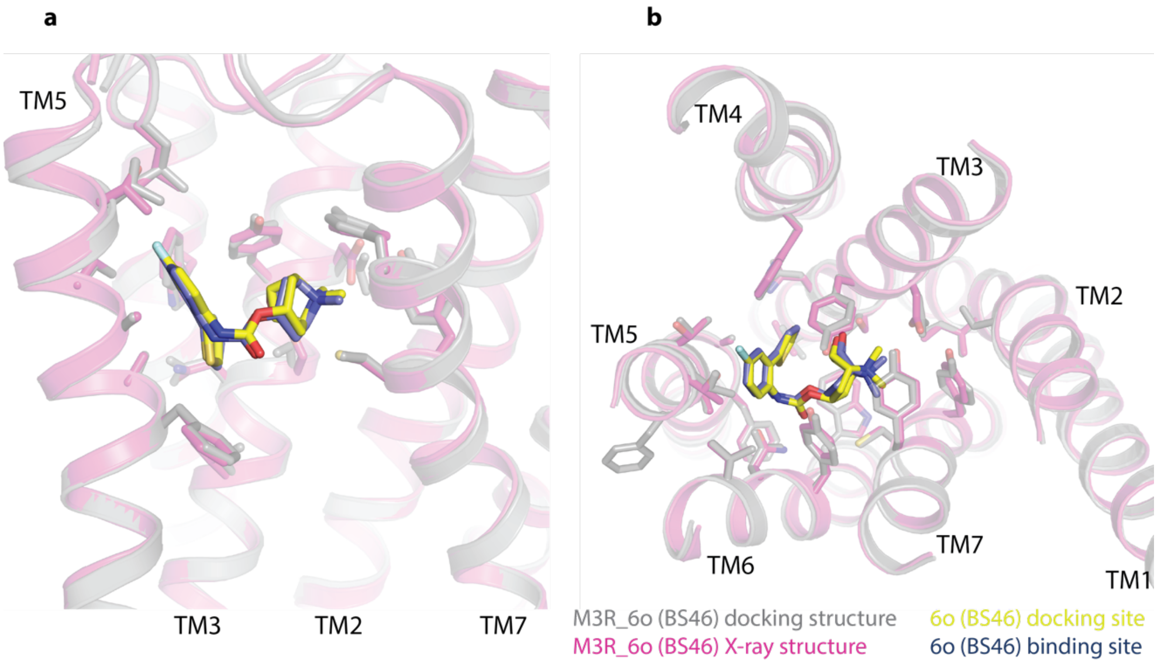

Supp. Table S1.

Binding kinetics of compound **6o (BS46)** compared with other muscarinic receptor antagonists.

	K3 (assoc constant) ($min^{-1}\cdot M^{-1}$)			K4 (dissoc constant) (min^{-1})			half-life (min)			Kd (nM)†			Ki (nM)		
**M3R***	**mean**	**SEM**	**n**	**mean**	**S.E.M.**	**n**	**mean**	**SEM**	**n**	**mean**	**SEM**	**n**	**mean**	**SEM**	**n**
**Compound 6o**	1.1 $\times 10^8$	7.1 $\times 10^6$	5	0.0013	0.0005	5	890	230	5	0.012	0.004	5	0.027	0.003	4
**tiotropium**	1.2 $\times 10^8$	1.1 $\times 10^7$	6	0.0019	0.0006	6	1300	600	6	0.017	0.006	6			
**darifenacin**	9.6 $\times 10^6$	4.5 $\times 10^5$	5	0.0052	0.0006	4	140	20	4	0.52	0.05	4	0.25	0.09	3
**[^{3}H]-NMS**	3.0 $\times 10^8$	6.2 $\times 10^7$	3	0.011	0.001	3	63	5	3						
**M2R****															
**Compound 6o**	3.0 $\times 10^8$	3.1 $\times 10^7$	3	0.12	0.01	3	6.1	0.1	3	0.41	0.04	3	0.66	0.03	3
**tiotropium**	6.5 $\times 10^8$	2.9 $\times 10^7$	3	0.017	0.001	3	42	4	3	0.026	0.003	3			
**darifenacin**	2.2 $\times 10^7$	4.5 $\times 10^6$	3	0.21	0.01	3	3.3	0.1	3	13	2	3	19	3	3
**[^{3}H]-NMS**	1.1 $\times 10^9$	1.4 $\times 10^8$	4	0.084	0.002	5	8.2	0.1	5						

**measured in 20 mM HEPES, pH 7.4, 100 mM NaCl*
***measured in 10 mM HEPES, pH 7.4, 10 mM NaCl, 0.5 mM $MgCl_2$*
† calculated from K4/K3

Supp. Table S2.

Binding affinity of antagonists for all muscarinic receptor subtypes.

Compound	M1R Ki (nM)	M2R Ki (nM)	M3R Ki (nM)	M4R Ki (nM)	M5R Ki (nM)	M2R Ki/ M3R Ki
[^{3}H]-NMS	0.042 ± 0.010	0.22 ± 0.02	0.068 ± 0.012	0.021 ± 0.003	0.089 ± 0.004	3.2
QNB	0.040 ± 0.010	0.098 ± 0.025	0.13 ± 0.02	0.033 ± 0.007	0.12 ± 0.02	0.75
Darifenacin	2.8 ± 0.2	19 ± 3	0.25 ± 0.09	2.4 ± 0.2	0.70 ± 0.09	76
6b	0.11 ± 0.01	21 ± 4	0.20 ± 0.03	0.12 ± 0.02	0.13 ± 0.02	105
6i	0.047 ± 0.005	15 ± 2	0.16 ± 0.02	0.062 ± 0.002	0.070 ± 0.006	94
6k	0.093 ± 0.003	7.2 ± 1.0	0.13 ± 0.01	0.080 ± 0.006	0.063 ± 0.001	55
6l	0.079 ± 0.003	8.3 ± 2.2	0.19 ± 0.06	0.060 ± 0.002	0.053 ± 0.003	44
6o	0.011±0.002	0.66±0.31	0.027±0.003	0.009±0.002	0.047±0.011	24

Ki was measured by competition with 0.05-0.2 nM [^{3}H]-NMS, depending on the receptor subtype, in 10 mM HEPES, pH 7.4, 10 mM NaCl, and 0.5 mM $MgCl_2$ for 2 hr (M2R) or 3 hr (M3R) at room temp

Ki values are the mean ± S.E.M. from at least three separate assays.

Supp. Table S3.

Crystallography data collection and refinement statistics

Data Collection‡	
Number of crystals	93
Space group	P2
Cell dimensions	
a, b, c (Å)	122.1, 53.1, 124.7
α,β,γ (°)	90.0, 98.7, 90.0
Number of reflections	245517
Number of unique reflections	29106
Resolution (Å)	50 – 3.1 (3.2-3.1)
R_{merge}	0.261 (1.278)
$CC_{1/2}$ (%)	99.1 (53.6)
$\langle I/\sigma I \rangle$	6.63 (1.04)
Completeness (%)	99.3 (97.0)
Redundancy	8.4 (4.3)
Refinement	
Resolution (Å)	20-3.1
Number of reflections (test set)	26494 (2177)
R_{work}/R_{free}	0.228/0.266
Number of atoms	
Receptor	4348
mT4L Lysozyme	1854
**6o (BS46)**	98
Others (Lipids, ions, waters)	95
Average B factors (Å^2)	
Receptor	86.5
mT4L Lysozyme	74.0
**6o (BS46)**	81.1
Others (Lipids, ions, waters)	95.5
RMS deviation from ideality	
Bond length (Å)	0.002
Bond angles (°)	0.493
Ramachandran statistics§	
Favored region (%)	98.85
Allowed region (%)	1.15
Outliers (%)	0

‡Highest resolution shell statistics are shown in parentheses.

§As defined by MolProbity.

Supp. Table S4.

GPCR targets and experimental conditions for the investigation of receptor selectivity of compound **6o (BS46)** in radioligand binding experiments.

GPCR[a]	radioligand		homogenate		
	radioligand[b]	conc RL [nM]	Kd [nM]	Bmax [fmol/mg]	protein [µg/well]
alpha1A	[^{3}H]prazosin	0.20	0.080	1400	6
alpha2A	[^{3}H]RX821002	0.50	0.45	2100	6
beta1	[^{3}H]CGP12177	0.20	0.10	2500	2
beta2	[^{3}H]CGP12177	0.20	0.050	2500	3
D1	[^{3}H]SCH23390	0.30	0.23	3500	2
$D2_{long}$[c]	[^{3}H]spiperone	0.20	0.15	1700	4
$D2_{short}$	[^{3}H]spiperone	0.20	0.090	1700	3
D3	[^{3}H]spiperone	0.20	0.090	2000	4
D4	[^{3}H]spiperone	0.30	0.15	1000	6
D5	[^{3}H]SCH23390	0.50	0.50	1500	10
5-HT1A	[^{3}H]WAY100635	0.20	0.10	3400	2
5-HT2A	[^{3}H]ketanserin	0.30	0.18	950	10
5-HT6	[^{3}H]LSD	0.75	2.1	2900	6
δOR	[^{3}H]diprenorphine	0.30	0.15	2500	6
κOR	[^{3}H]diprenorphine	0.20	0.080	2000	5
µOR	[^{3}H]diprenorphine	0.20	0.080	3200	2
NTS1	[^{3}H]NT8-13[d]	0.50	0.62	5000	4
NTS2	[^{3}H]NT8-13[d]	0.50	1.3	550	14

[a] Membranes were derived from HEK-293T cells transiently transfected with the cDNA of the appropriate receptor. [b] All radioligands were purchased by standard supplier for radiochemicals. [c] Membranes from CHO cells stably transfected with the long isoform of the D2 receptor (20). [d] [^{3}H]NT8-13 was available from custom synthesis of [leucine-^{3}H]NT(8-13) (GE Healthcare, Freiburg, Germany).

Supp. Table S5.

GPCR targets and experimental conditions for the investigation of receptor selectivity of compound **6o (BS46)** functional experiments (IP accumulation).

GPCR	gene	agonist	EC_{80} conc [nM]
PAR-2	humanPAR-2	f-LIGRLO-NH_2	100
OXR1	HCRTR1	orexin A	300
OXR2	HCRTR2	orexin A	300

References

1. Mysinger MM & Shoichet BK (2010) Rapid context-dependent ligand desolvation in molecular docking. J. Chem. Inf. Model. 50(9):1561-1573.
2. Case DA, Babin V, Berryman JT, Betz RM, Cai Q, Cerutti DS, Cheatham TE, Darden TA, Duke RE, H. Gohlke H, A.W. Goetz AW, S. Gusarov S, N. Homeyer N, P. Janowski P, J. Kaus J, I. Kolossváry I, A. Kovalenko A, Lee TS, LeGrand S, Luchko T, Luo R, Madej B, Merz KM, Paesani F, Roe DR, Roitberg A, Sagui C, Salomon-Ferrer R, Seabra G, Simmerling CL, Smith W, Swails J, Walker RC, Wang J, Wolf RM, Wu X and Kollman PA (2014), AMBER 14, University of California, San Francisco.
3. Wang J, Wang W, Kollman PA, & Case DA (2006) Automatic atom type and bond type perception in molecular mechanical calculations. J. Mol. Graph. Model. 25(2):247-260.
4. Mobley DL, et al. (2007) Predicting absolute ligand binding free energies to a simple model site. J. Mol. Biol. 371(4):1118-1134.
5. Carlsson J, et al. (2010) Structure-based discovery of A2A adenosine receptor ligands. J. Med. Chem. 53(9):3748-3755.
6. Caffrey M & Cherezov V (2009) Crystallizing membrane proteins using lipidic mesophases. Nat. Protoc. 4:706-731.
7. Kabsch W (2010) Xds. Acta Crystallographica Section D Biological Crystallography 66(2):125-132.
8. Liu H & Naismith JH (2008) An efficient one-step site-directed deletion, insertion, single and multiple-site plasmid mutagenesis protocol. BMC Biotechnol. 8(1):91.
9. Cheng Y-C & Prusoff WH (1973) Relationship between the inhibition constant (KI) and the concentration of inhibitor which causes 50 per cent inhibition (I50) of an enzymatic reaction. Biochem. Pharmacol. 22(23):3099-3108.
10. Möller D, et al. (2017) Discovery of G Protein-Biased Dopaminergics with a Pyrazolo[1,5-a]pyridine Substructure. J. Med. Chem. 60(7):2908-2929.
11. Guo D, et al. (2012) Dual-Point Competition Association Assay: A Fast and High-Throughput Kinetic Screening Method for Assessing Ligand-Receptor Binding Kinetics. J. Biomol. Screening 18(3):309-320.

12. Volgyesi GA, Tremblay LN, Webster P, Zamel N, & Slutsky AS (2000) A new ventilator for monitoring lung mechanics in small animals. J. Appl. Physiol. 89(2):413-421.
13. Fisher JT, Vincent SG, Gomeza J, Yamada M, & Wess J (2004) Loss of vagally mediated bradycardia and bronchoconstriction in mice lacking M2 or M3 muscarinic acetylcholine receptors. FASEB J. 18(6):711-713.
14. Scarpitta F, Busolli J, Diulgheroff N, Volonte R, & Pontiroli A (2008) WO2008008376A2.
15. Infante R, Nieto J, & Andres C (2012) Highly homogeneous stereocontrolled construction of quaternary hydroxyesters by addition of dimethylzinc to alpha-ketoesters promoted by chiral perhydrobenzoxazines and B(OEt)3. Chemistry 18(14):4375-4379.
16. Schuetz RD & Nilles GP (1971) Chemistry of Dithienyl Diketones .1. Synthetic Explorations. J. Org. Chem. 36(17):2486-2489.
17. Newman SG, Aureggi V, Bryan CS, & Lautens M (2009) Intramolecular cross-coupling of gem-dibromoolefins: a mild approach to 2-bromo benzofused heterocycles. Chem Commun (Camb) (35):5236-5238.
18. Chiba S, Zhang L, Ang GY, & Hui BW (2010) Generation of iminyl copper species from alpha-azido carbonyl compounds and their catalytic C-C bond cleavage under an oxygen atmosphere. Org. Lett. 12(9):2052-2055.
19. Latli B, et al. (2016) Buscopan labeled with carbon-14 and deuterium. J. Labelled Compd. Radiopharm. 59(13):557-564.
20. Hayes G, Biden TJ, Selbie LA, & Shine J (1992) Structural subtypes of the dopamine D2 receptor are functionally distinct: expression of the cloned D2A and D2B subtypes in a heterologous cell line. Mol. Endocrinol. 6(6):920-926.
